# Potential molecular mechanism of ACE gene at different time points in STEMI patients based on genome-wide microarray dataset

**DOI:** 10.1186/s12944-019-1131-3

**Published:** 2019-10-23

**Authors:** Yao-Zong Guan, Rui-Xing Yin, Peng-Fei Zheng, Guo-Xiong Deng, Chun-Xiao Liu, Bi-Liu Wei

**Affiliations:** 1 Department of Cardiology, Institute of Cardiovascular Diseases, the First Affiliated Hospital, Guangxi Medical University, 22 Shuangyong Road, Nanning, 530021 Guangxi People’s Republic of China; 2 Guangxi Key Laboratory Base of Precision Medicine in Cardio-cerebrovascular Disease Control and Prevention, Nanning, 530021 Guangxi People’s Republic of China; 3 Guangxi Clinical Research Center for Cardio-cerebrovascular Diseases, Nanning, 530021 Guangxi People’s Republic of China

**Keywords:** Co-expression genes, ACE, Gene ontology annotation, Kyoto encyclopedia of genes and genomes (KEGG) pathway, ST-segment elevation myocardial infarction

## Abstract

**Background:**

This study aimed to investigate the angiotensin converting enzyme (ACE) co-expression genes and their pathways involved in ST-segment elevation myocardial infarction (STEMI) at different time points.

**Methods:**

The array data set of GSE59867 was examined for the ACE co-expression genes in peripheral blood samples from 111 patients with STEMI at four time points (admission, discharge, and 1 and 6 months after MI). Kyoto Encyclopedia of Genes and Genomes (KEGG) pathway enrichment, Gene Ontology (GO) annotation and protein-protein interaction (PPI) of the co-expression genes were determined using online analytical tools. The Cytoscape software was used to create modules and hub genes.

**Results:**

The number of biological processes (BP), cellular components (CC) and molecular functions (MF) was 43, 22 and 24 at admission; 18, 19 and 11 at discharge; 30, 37 and 21 at 1 month after MI; and 12, 19 and 14 at 6 months after MI; respectively. There were 6 BP, 8 CC and 4 MF enriched at every time point. The co-expression genes were substantially enriched in 12, 5, 6 and 14 KEGG pathways at the four time points, respectively, but no KEGG pathway was found to be common in all time points. We identified 132 intersectional co-expression genes (90 positive and 42 negative) from the four time points and 17 BP, 13 CC, 11 MF and 7 KEGG pathways were enriched. In addition, the PPI network contained 129 nodes and 570 edges, and only 1 module was identified to be significantly enriched in just 1 BP (chromatin-mediated maintenance of transcription).

**Conclusions:**

The results of the present study showed that the ACE co-expression genes and their pathways involved in STEMI were significantly different at four different time points. These findings may be helpful for further understanding the functions and roles of ACE in different stages of STEMI, and providing reference for the treatment of STEMI.

## Introduction

Coronary artery disease (CAD) is one of the leading causes of mortality among cardiovascular and cerebrovascular diseases, responsible for approximately 700,000 deaths in China [1, 2]. ST-segment elevation myocardial infarction (STEMI) is the most common cause of mortality in patients with CAD. Although with the establishment of coronary care units, improvements in medical therapy, and widespread use of early reperfusion therapy by primary percutaneous coronary intervention (PCI), the in-hospital mortality after STEMI has dramatically decreased to ≈ 5%, cardiac deaths after discharge cannot be ignored [3].

Widely accepted risk factors for CAD include age, gender, hypertension, diabetes, smoking, dyslipidemia, family history, and genetic variation [4–6]. Current indicators of diagnosis and prognosis of myocardial infarction (MI) include electrocardiogram, troponin, myocardial enzyme, and left ventricular ejection fraction [7–9]. But the molecular mechanisms responsible for the development and progression of STEMI remain unclear. The angiotensin converting enzyme (ACE) is associated with vasoconstriction, inflammation, vascular remodeling, thrombosis, apoptosis, and eventual plaque rupture [10]. The present study aimed to utilize bioinformatics to identify genes co-expressed with *ACE* genes and pathways associated with STEMI at different time points and to provide stage specific therapy for patients.

In the present study, the GSE59867 microarray expression dataset was extracted from the Gene Expression Omnibus (GEO) database, a global free-access repository of next-generation sequence functional genomic data sets and high-throughput microarray [11]. A genome-wide co-expression screening was performed by cor function in the R platform. All intersectional co-expression genes at the four time points were determined and depicted with Venn Diagrams. Gene Ontology (GO) and Kyoto Encyclopedia of Genes and Genomes (KEGG) analyses were used to define biological functions of the intersectional co-expression genes and the co-expressing genes at different time points. The GO and KEGG pathways were compared to separate the common genes as well as unique genes throughout the whole SETMI process and at specific time points. Finally, the Search Tool for the Retrieval of Interacting Genes (STRING) [12] was used to construct protein-protein interaction (PPI) network and to detect the hub genes.

## Materials and methods

### Affymetrix microarray data

The GSE59867 gene expression dataset was retrieved from GPL6244 Affymetrix Human Gene 1.0 ST Array platform from the GEO database (https://www.ncbi.nlm.nih.gov/geo/query/acc.cgi?acc=GSE59867). The GSE59867 dataset contained 436 samples, out of which 390 samples from patients (*n* = 111) with STEMI at four time points (admission, discharge, 1 month after MI, and 6 months after MI) and 46 samples from patients (*n* = 46) with stable CAD and without a history of MI were included in the study [13]. The data extracted was normalized by limma package (http://www.bioconductor.org/packages/release/bioc/html/limma.html) in the R platform, which is a software package providing powerful facilities for reading, normalizing and exploring microarray data [14, 15].

### Identification of ACE co-expression genes

A genome-wide co-expression gene screening for ACE in patients with STEMI was performed by cor function in the R platform. The screening criteria were as follows: *P* < 0.05, and | Pearson correlation coefficient | ≥ 0.2. Then the online analytical tool Draw Venn Diagram (http://bioinformatics.psb.ugent.be/webtools/Venn/) was used to determine the intersectional co-expression genes at each time point.

### GO and KEGG pathway enrichment analyses

GO and KEGG enrichment analyses of the co-expression genes were conducted separately on the Database for Annotation, Visualization and Integrated Discovery (DAVID) (version 6.8). Statistical significance was set at *P*-value < 0.05. The results were visualized by the R-ggplot2 package (version 3.5.3).

### Integration of the PPI network

Interactions among the co-expression genes were evaluated using the STRING (version 10.5) database; a combined score of > 0.15 was considered statistically significant interaction. In addition, the Cytoscape plugin cytoHubba (version 0.1), a package common used to identified the hub objects and sub-networks from complex interaction [14, 16], with the ranking methods of maximal clique centrality (MCC) was used to identify the top 10 hub genes.

## Results

### Identification of ACE co-expression genes

**Fig. 1:**
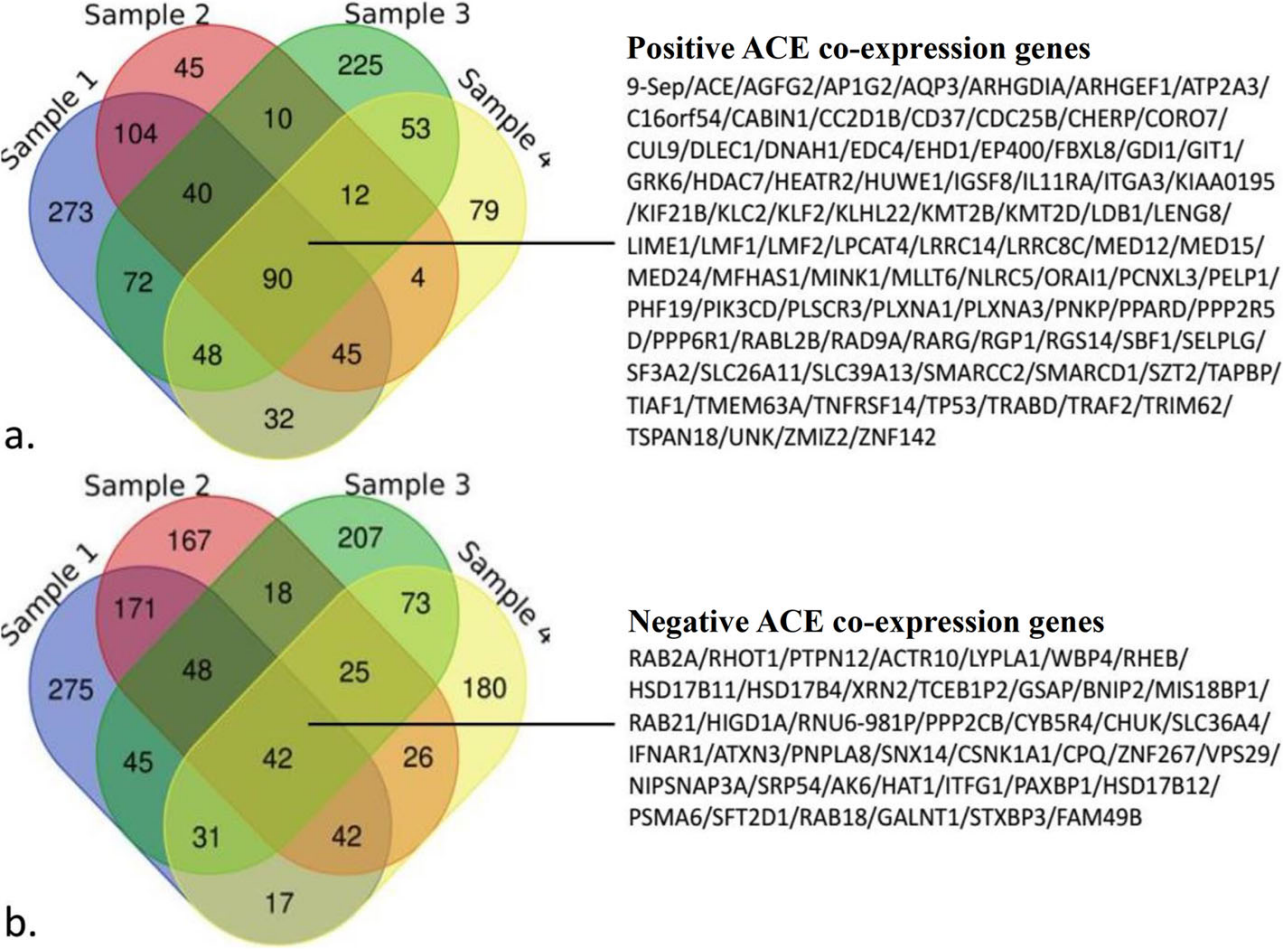
Venn diagram of co-expressing genes. **a.** Venn diagram of the positively ACE co-expressing genes. **b.** Venn diagram of the ACE negatively co-expressing genes. Sample 1, ACE co-expressing genes identified at the time of admission. Sample 2, ACE co-expressing genes identified at the time of discharge. Sample 3, ACE co-expressing genes identified at 1 month after ST-segment elevation myocardial infarction (STEMI). Sample 4, ACE co-expressing genes identified at 6 months post STEMI

A total of 704 positively and 671 negatively co-expressed genes at admission, 350 positively and 489 negatively co-expressed genes at discharge, 550 positively and 539 negatively co-expressed genes at 1 month after MI, and 363 positively and 436 negatively co-expressed genes 6 months after MI were identified in this study. Figure 1 depicts a Venn diagram showing the 90 positively and 42 negatively co-expressed genes common across the time points.

### Functional analysis of GO and KEGG pathways enrichment of co-expression genes

**Fig. 2:**
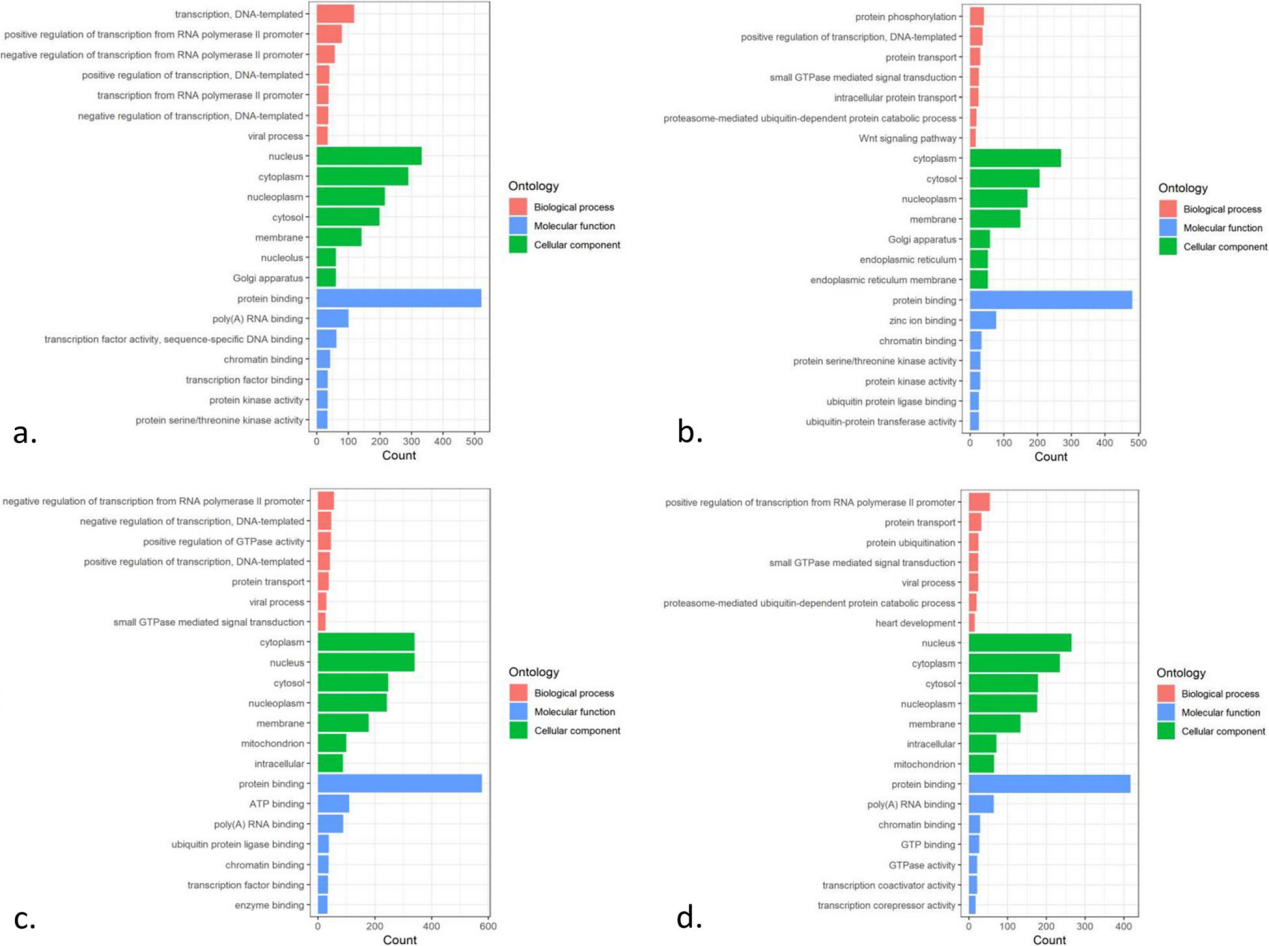
GO analysis for ACE co-expression genes at four time points of ST-segment elevation myocardial infarction (STEMI). **a.** GO analysis for ACE co-expression genes at admission of STEMI. **b.** GO analysis for ACE co-expression genes at discharge. **c.** GO analysis for ACE co-expression genes at 1 month after STEMI. **d.** GO analysis for ACE co-expression genes at 6 months after STEMI. Red column, blue column, and green column separately represents the Biological process (BP), Molecular function (MF), and Cellular component (CC), and the length of column represents the count

**Fig. 3:**
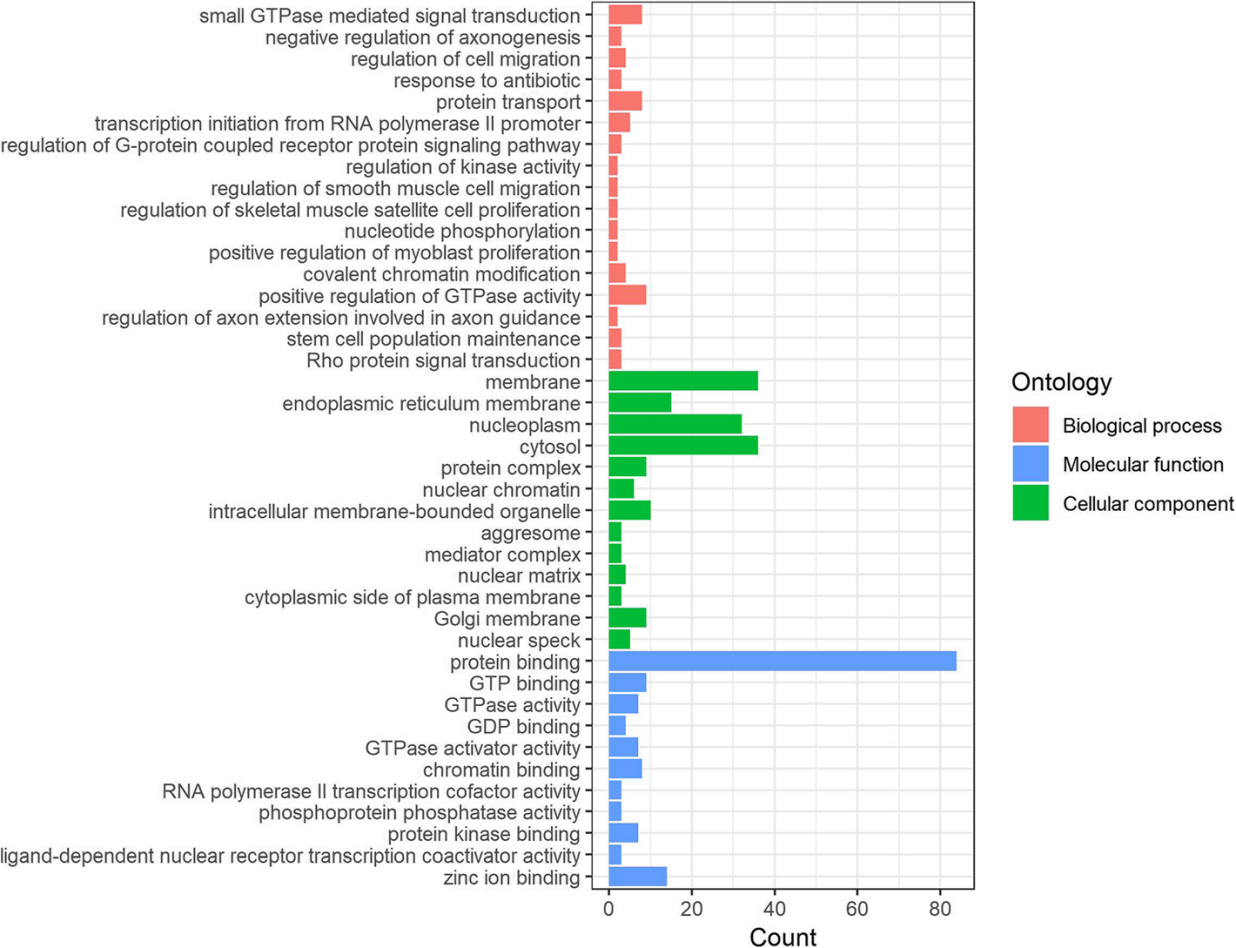
GO analysis for intersectional ACE co-expression genes. Red column, blue column, and green column separately represents the Biological process (BP), Molecular function (MF), and Cellular component (CC), and the length of column represents the count

GO function clustering revealed 43 biological processes (BP), 22 cellular components (CC), and 24 molecular functions (MF) at the time of admission; 18 BP, 19 CC, and 11 MF were identified at the time of discharge; 30 BP, 37 CC, and 21 MF were identified at the time of 1 month after MI; and 12 BP, 19 CC, and 14 MF were identified at the time of 6 month after MI (Additional file 1: Tables S1-S4). We selected the top seven BP, CC, and MF in descending order of count with *P* < 0.01 at different time points for visualization (Fig. 2). Six BP (intracellular protein transport, positive regulation of transcription/DNA-templated, proteasome-mediated ubiquitin-dependent protein catabolic process, protein transport, small GTPase mediated signal transduction, Wnt signaling pathway), 4 MF (chromatin binding, GTPase activator activity, GDP binding, protein binding) and 8 CC (cytoplasm, cytosol, intracellular, nucleus, catalytic step 2 spliceosome, Golgi apparatus, membrane, nucleoplasm) were shared at all the time points. The analysis further identified 17 BP, 13 CC and 11 MF as intersectional co-expression genes (Table 1 and Fig. 3).

**Table 1:** GO analysis for intersectional ACE co-expression genes

Category	ID	Description	Count	*P*-Value	Gene ID
BP	GO:0007264	small GTPase mediated signal transduction	8/200	1.77E-03	RAB2A/GDI1/RAB18/MFHAS1/RHOT1/RHEB/RAB21/RABL2B
BP	GO:0050771	negative regulation of axonogenesis	3/200	7.74E-03	GDI1/ARHGEF1/ARHGDIA
BP	GO:0030334	regulation of cell migration	4/200	1.53E-02	PLXNA3/PLXNA1/LDB1/MINK1
BP	GO:0046677	response to antibiotic	3/200	2.12E-02	CYB5R4/PPP2CB/TP53
BP	GO:0015031	protein transport	8/200	2.15E-02	SFT2D1/RAB2A/VPS29/GDI1/RAB18/SNX14/CORO7/RAB21
BP	GO:0006367	transcription initiation from RNA polymerase II promoter	5/200	2.24E-02	PPARD/RARG/MED15/MED12/MED24
BP	GO:0008277	regulation of G-protein coupled receptor protein signaling pathway	3/200	3.06E-02	GIT1/GRK6/RGS14
BP	GO:0043549	regulation of kinase activity	2/200	3.46E-02	NLRC5/LDB1
BP	GO:0014910	regulation of smooth muscle cell migration	2/200	3.46E-02	ACE/PLXNA1
BP	GO:0014842	regulation of skeletal muscle satellite cell proliferation	2/200	4.14E-02	PPARD/PAXBP1
BP	GO:0046939	nucleotide phosphorylation	2/200	4.14E-02	PNKP/AK6
BP	GO:2000288	positive regulation of myoblast proliferation	2/200	4.14E-02	PPARD/PAXBP1
BP	GO:0016569	covalent chromatin modification	4/200	4.54E-02	PHF19/SMARCC2/SMARCD1/CABIN1
BP	GO:0043547	positive regulation of GTPase activity	9/200	4.58E-02	GIT1/GDI1/ARHGEF1/SBF1/BNIP2/AGFG2/RGP1/ARHGDIA/RGS14
BP	GO:0048841	regulation of axon extension involved in axon guidance	2/200	4.82E-02	PLXNA3/PLXNA1
BP	GO:0019827	stem cell population maintenance	3/200	4.82E-02	PHF19/MED12/MED24
BP	GO:0007266	Rho protein signal transduction	3/200	4.82E-02	ARHGEF1/ARHGDIA/CHUK
CC	GO:0016020	membrane	36/200	6.08E-07	ORAI1/PLXNA3/GALNT1/CHERP/AP1G2/AGFG2/LRRC8C/LMF2/EDC4/RGP1/KLC2/TAPBP/SFT2D1/ACE/FAM49B/PELP1/HSD17B4/EHD1/SELPLG/CSNK1A1/GIT1/MED12/CORO7/AK6/IL11RA/LPCAT4/PNKP/PNPLA8/IGSF8/CD37/MED15/HUWE1/GRK6/RHOT1/RHEB/XRN2
CC	GO:0005789	endoplasmic reticulum membrane	15/200	1.88E-03	RAB2A/CYB5R4/GALNT1/LMF2/LRRC8C/HSD17B12/LMF1/LPCAT4/TAPBP/PNPLA8/ATXN3/RAB18/ATP2A3/RHEB/RAB21
CC	GO:0005654	nucleoplasm	32/200	2.42E-03	KMT2D/PPARD/PPP2R5D/KMT2B/HAT1/MED24/EDC4/WBP4/SLC26A11/PELP1/CC2D1B/CHUK/RARG/TP53/MED12/RAD9A/SF3A2/AK6/CDC25B/PNKP/ATXN3/PHF19/PSMA6/MED15/HUWE1/ZMIZ2/SMARCC2/CABIN1/MIS18BP1/EP400/XRN2/HDAC7
CC	GO:0005829	cytosol	36/200	2.94E-03	VPS29/HSD17B11/TRAF2/PPP2R5D/PPP6R1/NIPSNAP3A/EDC4/RGP1/KLC2/PAXBP1/NLRC5/PPP2CB/CHUK/ARHGDIA/CSNK1A1/GIT1/GDI1/SRP54/ARHGEF1/PIK3CD/TP53/MINK1/CORO7/STXBP3/LYPLA1/TRIM62/PTPN12/CDC25B/ATXN3/RAB18/PSMA6/HUWE1/BNIP2/FBXL8/RHOT1/RHEB
CC	GO:0043234	protein complex	9/200	6.55E-03	GDI1/ORAI1/LDB1/SMARCC2/TP53/HAT1/RGP1/KLC2/HIGD1A
CC	GO:0000790	nuclear chromatin	6/200	9.67E-03	PPARD/RARG/LDB1/SMARCC2/TP53/HAT1
CC	GO:0043231	intracellular membrane-bounded organelle	10/200	1.27E-02	HSD17B11/VPS29/PLXNA3/BNIP2/HAT1/EDC4/CC2D1B/HSD17B4/SLC26A11/CHUK
CC	GO:0016235	aggresome	3/200	2.05E-02	GIT1/CABIN1/XRN2
CC	GO:0016592	mediator complex	3/200	2.29E-02	MED15/MED12/MED24
CC	GO:0016363	nuclear matrix	4/200	2.74E-02	ATXN3/PSMA6/TP53/HAT1
CC	GO:0009898	cytoplasmic side of plasma membrane	3/200	3.22E-02	TRAF2/RAB21/CHUK
CC	GO:0000139	Golgi membrane	9/200	4.51E-02	RAB2A/PNPLA8/GALNT1/AP1G2/PPP6R1/RHEB/CORO7/RGP1/TAPBP
CC	GO:0016607	nuclear speck	5/200	4.62E-02	CSNK1A1/SRP54/SF3A2/WBP4/EP400
MF	GO:0005515	protein binding	84/200	2.44E-06	PPARD/PLXNA3/CHERP/AP1G2/PPP2R5D/LRRC8C/NIPSNAP3A/EDC4/MED24/RGP1/WBP4/TAPBP/NLRC5/FAM49B/MFHAS1/CUL9/SMARCD1/KLHL22/CHUK/RAB21/RARG/ARHGEF1/LDB1/PIK3CD/TP53/MED12/MINK1/CORO7/TNFRSF14/STXBP3/RAD9A/LENG8/IFNAR1/PNKP/CD37/IGSF8/PSMA6/HUWE1/MED15/RAB18/FBXL8/BNIP2/ZMIZ2/EP400/XRN2/VPS29/TRAF2/KMT2D/ORAI1/SLC39A13/LMF2/KMT2B/HSD17B12/PPP6R1/HAT1/KLC2/PELP1/PPP2CB/CC2D1B/MLLT6/EHD1/SELPLG/ARHGDIA/CSNK1A1/GIT1/RAB2A/GDI1/ SRP54/ITGA3/SF3A2/AK6/PTPN12/RGS14/CDC25B/ATXN3/PHF19/SMARCC2/PLSCR3/RHOT1/GRK6/MIS18BP1/KLF2/HDAC7/ACTR10
MF	GO:0005525	GTP binding	9/200	4.29E-03	RAB2A/SRP54/RAB18/MFHAS1/RHOT1/RHEB/EHD1/RAB21/RABL2B
MF	GO:0003924	GTPase activity	7/200	4.91E-03	RAB2A/SRP54/RAB18/RHOT1/RHEB/RAB21/RABL2B
MF	GO:0019003	GDP binding	4/200	5.65E-03	RAB2A/SRP54/RAB18/RAB21
MF	GO:0005096	GTPase activator activity	7/200	1.12E-02	GIT1/GDI1/ARHGEF1/BNIP2/AGFG2/ARHGDIA/RGS14
MF	GO:0003682	chromatin binding	8/200	1.61E-02	PELP1/LDB1/SMARCC2/SMARCD1/TP53/MED12/ EP400/HDAC7
MF	GO:0001104	RNA polymerase II transcription cofactor activity,	3/200	2.41E-02	MED15/MED12/MED24
MF	GO:0004721	phosphoprotein phosphatase activity	3/200	3.65E-02	PPP2CB/PTPN12/CDC25B
MF	GO:0019901	protein kinase binding	7/200	4.07E-02	TRAF2/TP53/RHEB/RAD9A/RGS14/HDAC7/CDC25B

**Fig. 4:**
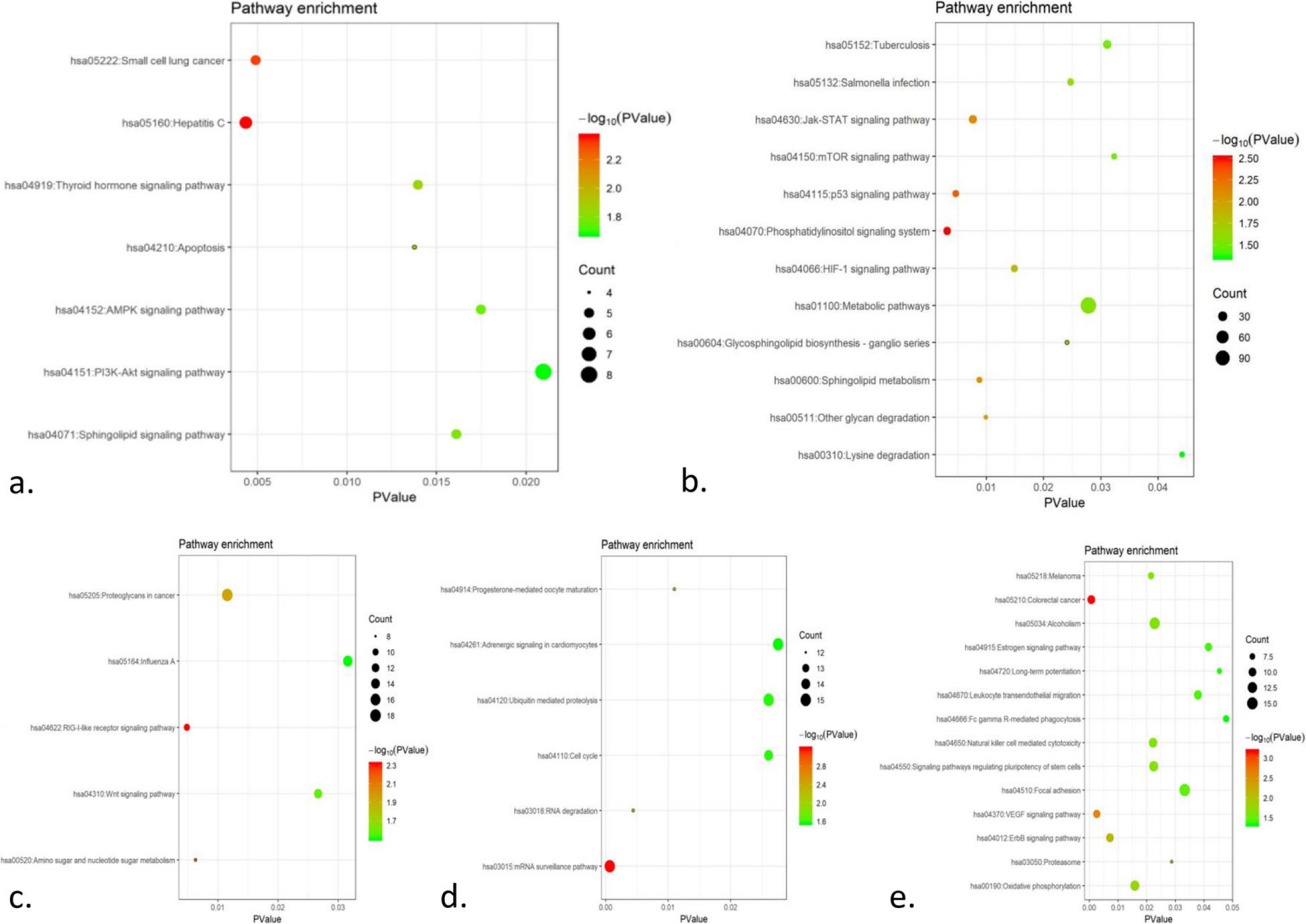
KEGG pathway enrichment analyses for ACE co-expression genes at four time points of ST-segment elevation myocardial infarction (STEMI). **a.** KEGG pathway based on the intersectional ACE co-expression genes at all the time points. **b.** KEGG pathway based on the ACE co-expression genes at admission of STEMI. **c.** KEGG pathway based on the ACE co-expression genes at discharge of STEMI. **d.** KEGG pathway based on the ACE co-expression genes at 1 month after STEMI. **e.** KEGG pathway based on the ACE co-expression genes at 6 months after STEMI. The sequence of bubble colors is red -green from high score to low score of –log_10_ (*P*-value), and the size of bubble represents the count

The KEGG pathway analysis showed that genes co-expressing with ACE genes were substantially enriched in 12, 5, 6 and 14 pathways at each time points, respectively (Additional file 1: Tables S5-S8) The intersectional co-expression genes mainly clustered into 7 pathways, including hepatitis C, small cell lung cancer, apoptosis, thyroid hormone signaling pathway, sphingolipid signaling pathway, AMPK signaling pathway, and PI3K-Akt signaling. However, no KEGG pathway was found to be common across the four time points (Fig. 4).

### PPI network construction and hub gene identification

**Fig. 5:**
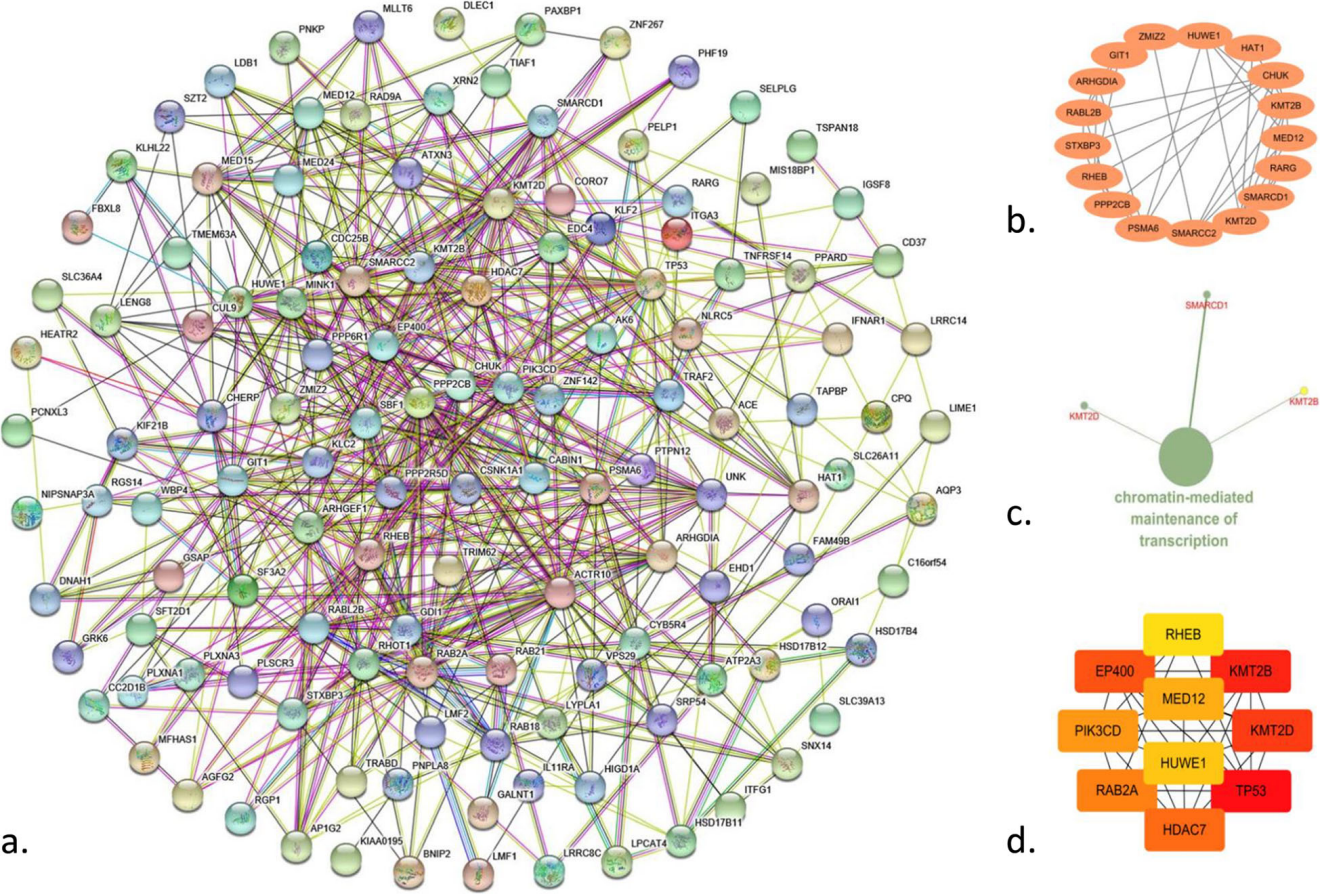
The protein-protein interaction (PPI) analysis of intersectional ACE co-expression genes of ST-segment elevation myocardial infarction (STEMI). **a.** PPI network based on the intersectional ACE co-expression genes of four time points. Nodes represent the genes and edges represent the co-expressive relation. **b.** Module enriched base on the PPI network with a cutoff criterion of MCODE score > 4. Nodes represent the genes and edges represent the co-expressive relation. **c.** Biological process of the genes of the module identified from the PPI network. The larger node represents the biological process and the others represent the enriched genes. **d.** Top 10 hub genes of the PPI network based on the intersectional ACE co-expression genes of four time points. The color depth represents the ranking of hub genes. The sequence of colors is red-orange-yellow from high ranking to low ranking

A PPI network with 129 nodes and 570 edges was constructed to detect the interactions among the co-expressing genes with a combined score > 0.15. With a cutoff criterion of MCODE score > 4, only 1 module was identified, which was significantly enriched in just 1 biological process chromatin-mediated maintenance of transcription with co-expressing genes *KMT2D*, *KMT2B* and *SMARCD1* (Fig. 5).

## Discussion

Over 80 million people have cardiovascular disease (CVD) in the United States, resulting in over 7 million revascularization procedures each year. Among them, STEMI accounts for a large proportion [17]. Not only the risk of death at the time of admission, but also the complications like dysfunction or rupture of papillary muscle, rupture of the heart, coronary stent thrombosis, malignant arrhythmia, post-infarction syndrome and heart failure give rise to the mortality [10, 18–20]. The biological and regulatory mechanisms in the early or post-myocardial infarction stages remain understudied. The rapid development of microarray expression data and bioinformatics has offered improved methods and the tools to better understand complex diseases, like CVD, diabetes, and cancer. It is widely accepted that renin-angiotensin-aldosterone system (RAAS) is activated in myocardial ischemia and heart failure [21]. The ACE localized mainly in the endothelium and smooth muscle, promotes the conversion of angiotensin II (Ang II, a potent vasoconstrictor and growth factor) from angiotensin I in the renin-angiotensin system (RAS), and degrades bradykinin. Ang II activates its receptor AT1, which in turn activates fibroblasts, promoting myocardial fibrosis and scar formation. Furthermore, Ang II destabilizes Kv4.3 messenger RNA, resulting in decrease of outward potassium and prolongation of action potential duration, finally induces arrhythmias and heart failure [22, 23]. Interestingly, previous studies suggested that ACE can regulate the immune-related cytokines, IL-12, tumor necrosis factor-α (TNF-α) and nitric oxide (NO), but finally proved the effect was due to the activation of AT1 by Ang II [24, 25]. A previous report by Tham et al showed that Ang II could regulate inflammation by down-regulated the PPAR receptors [25]. Thus, ACE involves in many mechanisms of physiology and pathophysiology with its central role in production of Ang II, chronic over-expression of tissue ACE causes over-production of Ang II. At the same time, decrease in bradykinin reduces the vasodilatory, profibrinolytic, antioxidant and antiapoptotic effects [26]. In the past decades, ACE inhibitors have been well received clinically with remarkable success based on their powerful effect on decreasing the production of Ang II [27]. In the present study, we identified genes co-expressed with *ACE*, and their GO enrichments and KEGG pathways at four time points of the STEMI. The analysis also identified the intersectional co-expression genes in all the time points to determine the GO enrichments and KEGG pathways throughout the stages of MI and the recovery process, and the unique GO enrichments and KEGG pathways at different time points of STEMI.

Identification of co-expressed genes is the cornerstone of BP, CC, MF and enrichment pathway analysis. Using the Venn diagram, we identified 90 positively and 42 negatively co-expressing genes of the intersection of the four time points. The gene *SELPLG*, also called *PSGL-1* was reported to be expressed at a high level in CD4+ T-Cells from patients with plaque rupture or intracoronary thrombus. It was also implicated in plaque instability in acute coronary syndrome (ACS) [28–30]. The *KLF2* gene is a vascular homeostasis-associated molecular marker, which regulates the expression of a wide range of anti-inflammatory, antioxidant, and antithrombotic genes in endothelial cells. Compared with patients with stable angina pectoris or normal controls, *KLF2* expression in dendritic cells in patients with ACS was found to be reduced [31, 32]. Some previous study reported that FBW7 tumor suppressor induces endothelial differentiation by modulating the NF1/RAS axis. The SCF^FBW7-RBX2/SAG^ (an ubiquitin ligase) activates RAS by promoting the degradation of NF1, and KLF2 acts as physiological substrate of FBW7. These results suggest that expression of KLF2 may indirectly affect the activity of ACE [33]. P53, the known tumor suppressor with characteristic of promoting apoptosis, was also reported to be associated with CAD [34–36]. What’s more, the TNF signals can activate both renin and p53 by inhibiting phosphoinositide 3-kinases (PI3Ks) via JNK pathway [37, 38]. This may be one of the reasons for the positive correlation between the expression of *ACE* and *P53*. In our study, *PSGL-1, KLF2* and *P53* were among the positively co-expression genes of *ACE* at every time points of STEMI, that is to say, high-expressed ACE is accompanied by high-expressed *PSGL-1*, *KLF2* and *P53*. ACE mainly mediates the production of Ang II, promoting the activation of inflammation and apoptosis, co-expressed of it, *PSGL-1* and *p53* may promote the inflammation and apoptosis, while *KLF2* plays the opposite role involved in the progression of STEMI. Whether *ACE* positively regulates these genes directly or through a feedback way is still the direction of our next step would focus on.

On the other hand, the *PNPLA1-PNPLA9* are members of the *PNPLA* family, playing different biological functions. Among them, *PNPLA2* is a key enzyme in the hydrolysis of stored triglycerides (TG), while *PNPLA8* is a myocardial phospholipase, maintaining mitochondrial integrity [39]. The *GALNT* family with lipase and transacylase properties appeared to play major roles in the regulation of lipid metabolism [40]. In addition, data from Pulido et al [41] supported that *RAB18* is a common mediator of lipolysis and lipogenesis, and suggested that the endoplasmic reticulum is the link that connected *RAB18* action on these two processes. In the current study, we showed that the *PNPLA, RAB18* and *GALNT1* were the negatively co-expressed genes of ACE at all the time points, in other words, increased ACE expression was accompanied by decreased expression of these genes. In the development of STEMI, decreased expression of *PNPLA2* may decrease hydrolysis of stored TG to reduce the serum TG levels, while decreased expression of *PNPLA8* may weaken mitochondrial integrity and reduce energy supply. In context of *RAB18*, its decrease expression may be partly responsible for the changes in serum lipid levels. Passos-Silva et al showed that Ang (1–7) regulated the metabolism by increasing glucose uptake and lipolysis, and decreasing insulin resistance and dyslipidemia [42]. The expression of ACE may affect the expression of Ang (1–7), in turn affects the metabolism. Therefore, the expression of these genes may be negatively regulated by ACE or negative feedback from ACE, but it needs further researches to confirm.

GO and KEGG pathways enrichment analysis revealed several shared pathways across the time points. The Wnt signaling plays dominant roles in the pathology of CVDs including inflammation, fibrosis, intracellular cholesterol accumulation and heart failure following MI, mobilization and proliferation of cells in the endothelium and epicardium in an infarcted heart [43–46]. The endothelium is directly involved in heart disease, peripheral vascular disease, diabetes, insulin resistance, stroke, venous thrombosis, chronic kidney failure, metastasis, tumor growth, and severe viral infectious diseases directly [47]. After injury, migration, proliferation of endothelial cells, and reendothelialization of the vessel is essential in the restoring of blood vessel health. Many of these processes are regulated, and are dependent on small GTPases [17, 48]. Thus, regulation of the Wnt signaling pathway or promotion of endothelialization might attenuate cardiovascular events after STEMI and that studying the regulation of the small GTPase could be an interesting starting point.

Using the MCODE, we identified only 1 module enriched and just 1 biological process- chromatin-mediated maintenance of transcription from the PPI network. In addition, 10 hub genes were identified using CytoHubba with a high level of connectivity - *TP53* (*p53*), *KMT2B* (*MLL4*), *KMT2D, EP400* (*p400*), *HDAC7, RAB2A, PIK3CD* (*PI3K*), *MED12* (*OPA1*), *HUWE1,* and *RHEB* (*mTORC1*). In the context of cellular immunity and apoptosis, Rahnamoun et al. [49] reported a novel mechanism in which the mutant *TP53* and *KMT2B* cooperated to regulate aberrant enhancer activity and tumor-promoting gene expression in response to chronic immune signaling. The EP400 E1A-associated protein, which mediates H2A.Z incorporation at specific promoters, plays a major role in cell fate decisions; it promotes cell cycle progression and inhibits apoptosis or senescence [50]. Decrease in *MED12*, which is important for maintaining normal cristae structure and function, resulted in increased apoptosis and mitochondrial fragmentation, and thereby reduction in energy supplement [51]. Li et al. [52] reported that, compared to non - CAD controls, *HDAC7* mRNA expression level was markedly lower in monocytes of CAD patients. That means these genes either participate in or regulate immune response and apoptosis. In our study, *p53*, *KMT2B*, *KMT2D*, *EP400*, and *HDAC7* were positively co-expressed with ACE, and increase in their expression may inhibit apoptosis and decrease mitochondrial energy metabolism. As both immune cells and apoptosis play important roles in the development of CAD, these genes could be promising targets for intervention for the treatment of CAD.

For blood glucose and lipid metabolism, previous studies give us the point that *RAB2A* knockdown inhibited glucose-stimulated insulin secretion, what’s more, it also regulated both initiation and termination of autophagy in mammalian cells [53, 54]. Silencing the ligases, *HUWE1* and *NEDD4–1*, increased the cholesterol export from cells [55]. In the context of thrombosis and endothelial injury, the p110δ plays a partial role in aggregation and spreading of platelet, and PI3K-Akt is cardio-protective in ischemic pre-conditioning [56, 57]. The mechanistic target of rapamycin complex 1 (*mTORC1*), one of the major pathways associated with cellular energy sensing, mediates vascular endothelial function through modulation of ROS signaling [58]. These findings indicated that the hub genes *RAB2A*, *PI3K*, *HUWE1* and *mTORC1* are the regulators of glucose metabolism, lipid metabolism, platelet and endothelial function. Our results suggested that these genes were negatively co-expressed with ACE, and decrease in their expression may reduce insulin secretion, increases serum cholesterol levels or result in vascular endothelial dysfunction and are closely related to the occurrence and development of CAD. Although to date clear evidence that ACE directly or indirectly regulates their expression is still lack, in depth understanding of the regulation of these genes may provide effective strategies for the recovery of patients with STEMI.

Our study aims to explore the mechanism of STEMI at different times from the perspective of positively or negatively co-expressed genes with ACE, which is different from the previous approach of analysis based on up-regulation and down-regulation of genes. However, there are some limitations of our research. Firstly, the results of our study cannot definitively confirm whether ACE could directly or indirectly regulates these genes. Secondly, the data from GSE59867 does not provide the factors associated with CAD like age, gender and blood lipid levels for correction. Finally, some potential factors that we have not clear may interfere with the end results.

## Conclusions

In this study, genome-wide co-expressing genes based on the GSE59867 dataset were used to identify the functions and mechanism of ACE gene at different time points of STEMI. We found that the ACE co-expression genes and their pathways involved in STEMI were significantly different at four different time points. These findings may help to better understand the functions and roles of ACE and co-expression genes in STEMI, and provide reference for future treatment strategies. However, further studies are required to validate the role of these co-expressing genes and pathways involved in different stages of STEMI.

## Supplementary information

**Additional file 1: Table S1.** GO enrichment at admission of STMEI. **Table S2.** GO enrichment at discharge of STMEI. **Table S3.** GO enrichment at 1 month after STEMI. **Table S4.** GO enrichment at 6 months after STEMI. **Table S5.** KEGG pathway at admission of STMEI. **Table S6.** KEGG pathway at discharge of STMEI. **Table S7.** KEGG pathway at 1 month after STEMI. **Table S8.** KEGG pathway at 6 months after STEMI.

## Data Availability

The datasets used and/or analyzed during the current study are available from the Gene Expression Omnibus repository (https://www.ncbi.nlm.nih.gov/geo/query/acc.cgi?acc=GSE59867).

## Abbreviations

ACE: Angiotensin converting enzyme
BP: Biological processes
CAD: Coronary artery disease
CC: Cellular components
CVD: Cardiovascular disease
ER: Endoplasmic reticulum
GEO: Gene Expression Omnibus
GO: Gene Ontology annotation
KEGG: Kyoto Encyclopedia of Genes and Genomes pathway enrichment analyses
MCODE: Molecular Complex Detection
MF: Molecular functions
PCI: percutaneous coronary intervention
PPI: Protein-protein interaction
RAS: Renin-angiotensin system
STEMI: ST-segment elevation myocardial infarction
TG: triglycerides
