# Supplementary material for: Potential molecular mechanism of ACE gene at different time points in STEMI patients based on genome-wide microarray dataset
Source: Lipids Health Dis. 2019 Oct 23;18:184. doi: 10.1186/s12944-019-1131-3 (PMC6813054; doi:10.1186/s12944-019-1131-3)
Supplement: Supplementary file 1 — Additional file 1: Table S1. GO enrichment at admission of STMEI. Table S2. GO enrichment at discharge of STMEI. Table S3. GO enrichment at 1 month after STEMI. Table S4. GO enrichment at 6 months after STEMI. Table S5. KEGG pathway at admission of STMEI. Table S6. KEGG pathway at discharge of STMEI. Table S7. KEGG pathway at 1 month after STEMI. Table S8. KEGG pathway at 6 months after STEMI. [file 12944_2019_1131_MOESM1_ESM.docx]

Supplementary Table 1 GO enrichment and DEGs at admission

| Category | Term | Count | PValue | Genes |
| --- | --- | --- | --- | --- |
| BP | GO:0045944/positive regulation of transcription from RNA polymerase II promoter | 79 | 8.40E-08 | RAI1/FOSL2/E2F4/ARID4A/ELF4/ARID4B/MED25/SPI1/NFKB2/WBP2/CITED2/MEN1/GABPB1/HSF1/CREB3L2/RARA/BRD4/EXOSC9/RARG/FOXJ2/LDB1/RELA/TP53/MED12/HMGA1/HES1/PRKD2/SPAG8/EP300/ZMIZ2/ZMIZ1/KDM6B/KMT2D/CRTC2/HMGB1/CAMTA2/TNF/FIGLA/ELK1/HCFC1/ELK3/SRF/SUMO2/NIPBL/SMARCB1/POU2F2/AGO1/PKD1/AATF/PPP3CA/RUNX1/PIK3R2/PLAGL2/NFATC1/ZNF564/SREBF1/ASXL2/IKZF4/TAF4/CCPG1/MAML1/CREBBP/SMAD3/ABHD14B/SKI/SREBF2/RLF/DOT1L/MEF2D/MNAT1/MAPK14/THRAP3/PPRC1/IRF2/TBL1X/NFIC/ENG/RERE/ATAD2B |
| BP | GO:0016032/viral process | 34 | 6.57E-07 | USP7/XPO1/CRTC2/RAD23A/RBM15B/WASF2/NUP188/TBP/ABI1/DAXX/SUMO1/NUP214/PSMB1/CENPA/RBCK1/IL2RG/BRD4/ZYX/POM121C/KAT2A/TAF4/SP100/CREBBP/TP53/ATP6V1H/HNRNPA1/AAAS/EP300/EIF4A2/ATF7/TSC2/TCEB1/CARM1/SRCAP |
| BP | GO:0043488/regulation of mRNA stability | 17 | 7.62E-06 | ZFP36/XPO1/EXOSC9/ELAVL1/ZFP36L1/PSMF1/NUP214/PSMC6/PSMD14/PSMD12/PSMB1/SERBP1/PSMC1/HNRNPD/ANP32A/PSMD7/SAMD4B |
| BP | GO:0000122/negative regulation of transcription from RNA polymerase II promoter | 57 | 1.16E-05 | XPO1/AEBP1/PPARD/WFS1/SPI1/CBX4/MED25/NFKB2/CBX6/CITED2/MEN1/HSF1/RARA/ZFP36/SP100/RARG/RELA/TP53/HDAC10/ARID1A/HES1/EP300/RFC1/TXN/MNT/HMGB1/EID1/MTDH/TNF/HCFC1/CIC/NPAS1/UBE2D3/SORBS3/NIPBL/ATN1/GATAD2A/MEPCE/ZNF564/SREBF1/ZBTB7A/TAF3/PTPN2/CREBBP/SMAD3/SKI/PHF12/SREBF2/ATF7/SMARCC2/ZBTB4/IRF2/NFIC/TBL1X/ENG/RERE/HDAC7 |
| BP | GO:0071108/protein K48-linked deubiquitination | 8 | 4.72E-05 | USP19/OTUB1/BAP1/FAM188A/USP25/FAM63B/USP33/VCPIP1 |
| BP | GO:0006351/transcription, DNA-templated | 118 | 1.02E-04 | PRR13/RBM15B/CNOT3/TCEAL6/CBX4/TCEAL8/CBX6/CITED2/GABPB1/MIER2/ATF6B/RARA/SAMD4B/RARG/ZNF100/HDAC10/MTA1/ARID1A/HES1/PA2G4/RFC1/SCYL1/PIAS3/DNTTIP2/CRTC2/EID1/SETD1B/ZNF76/TRRAP/RRAGC/ATN1/SMARCB1/HNRNPD/THAP11/ASXL2/IKZF4/DDX1/SMAD3/SKI/ZBTB45/SREBF2/PHF2/PHF1/ATF7/SMARCC2/PPRC1/SCMH1/ZNF385A/HDAC7/RERE/HDAC6/POU6F1/PPARD/AEBP1/E2F4/FOSL2/ARID4B/TERF2IP/DAXX/MEN1/HSF1/ANP32A/SPIB/BRD4/ELMSAN1/SERTAD2/SP100/LDB1/TP53/ZNF687/ZNF335/ZMIZ2/NAB2/ZMIZ1/NCOA5/TXN/ZNF277/USP21/COMMD1/RBM39/CARM1/SRCAP/COMMD6/DPF2/KMT2D/PTOV1/ZNF552/KMT2B/CIC/SEC14L2/NPAS1/ZSCAN22/POU2F2/GATAD2A/POU2F1/AGO1/MLXIP/TCEA1/BAZ2A/VPS36/ZNF564/ZBTB7A/BRD2/NACC1/DTX1/PPP1R10/PHF12/RLF/MEF2D/ZNF672/BCORL1/HNRNPUL1/MAPK14/ZBTB4/ZNF764/TBL1X/NFIC/KDM4D |
| BP | GO:0006886/intracellular protein transport | 24 | 2.14E-04 | XPO1/STX5/ARFGAP3/AP4E1/IPO13/RABGAP1L/VPS41/AP4M1/CLTC/CD74/TSNARE1/TBC1D3B/TBC1D15/ACD/COPG1/USO1/SLU7/GGA1/KDELR1/VPS26A/VPS39/GGA3/AP3B1/HDAC6 |
| BP | GO:0035855/megakaryocyte development | 6 | 4.21E-04 | EP300/WASF2/ABI1/SRF/ZNF385A/PTPN11 |
| BP | GO:0098609/cell-cell adhesion | 25 | 6.46E-04 | VAPB/EIF5/WASF2/HCFC1/ABI1/KLC2/RAB1A/LARP1/ATXN2L/SLK/BAG3/PAK4/ARHGAP1/FASN/RAB11B/NDRG1/STX5/CKAP5/CBL/LYPLA2/EIF4G2/SCYL1/SERBP1/USO1/MAPRE1 |
| BP | GO:0045893/positive regulation of transcription, DNA-templated | 39 | 8.26E-04 | RAI1/PPARD/FGF7/TNF/ELF4/SPI1/ELK1/MED24/TBP/ELK3/SEC14L2/CITED2/HNRNPD/CREB3L2/RARA/RUNX1/NFATC1/SERTAD2/DVL3/TAF4/SP100/MAP2K1/FOXJ2/MAP2K2/RELA/CREBBP/TP53/MED12/SMAD3/ARID1A/HMGA1/RLF/RFC1/TAF15/DYRK1B/SMARCC2/THRAP3/USP21/TBL1X |
| BP | GO:0030433/ER-associated ubiquitin-dependent protein catabolic process | 10 | 9.69E-04 | USP19/PSMC6/SYVN1/KIAA0368/WFS1/FOXRED2/PSMC1/CCDC47/UBXN4/SEL1L |
| BP | GO:0001889/liver development | 11 | 1.19E-03 | HES1/UPF2/KRAS/PHF2/RELA/HNRNPD/PKD1/SMAD3/RARA/SEC63/CITED2 |
| BP | GO:0043161/proteasome-mediated ubiquitin-dependent protein catabolic process | 20 | 1.20E-03 | RAD23A/TP53/MTA1/PCNP/PSMF1/PSMC6/UBE2D3/RNF44/PSMD14/RNF115/PSMD12/PSMB1/GSK3A/PPP2CB/PSMC1/RBCK1/SIAH1/FAF1/TBL1X/PSMD7 |
| BP | GO:1900034/regulation of cellular response to heat | 11 | 1.32E-03 | NUP214/AKT1S1/EP300/AAAS/HSF1/BAG3/CREBBP/DNAJC7/NUP188/MLST8/POM121C |
| BP | GO:0006357/regulation of transcription from RNA polymerase II promoter | 34 | 1.40E-03 | HMGN1/HMGB1/CAMTA2/PPARD/FOSL2/ARID4A/ZNF76/ARID4B/TCEAL6/MED24/TCEAL8/NFKB2/NPAS1/GABPB1/SMARCB1/ATF6B/SMARCD1/CTDSP1/SPIB/TCEA1/ELMSAN1/SREBF1/KAT2A/BRD2/TAF4/ZMYM3/SMAD3/ARID1A/ANXA4/MNAT1/MAPK14/SMARCC2/TCEB1/SRCAP |
| BP | GO:0000398/mRNA splicing, via spliceosome | 21 | 1.44E-03 | BCAS2/TRA2B/PTBP1/ELAVL1/SF3A2/HNRNPA1/SF3A1/SF3B4/SRSF3/HNRNPA3/HNRNPM/SRRT/HNRNPH3/DDX46/HNRNPUL1/CPSF7/ISY1/SYF2/HNRNPD/SLU7/SNRPE |
| BP | GO:0038061/NIK/NF-kappaB signaling | 10 | 1.93E-03 | PSMF1/PSMD14/PSMC6/PSMD12/PSMB1/PSMC1/RIPK3/NFKB2/MAP3K14/PSMD7 |
| BP | GO:0050821/protein stabilization | 15 | 2.12E-03 | USP7/DVL3/SYVN1/WFS1/HCFC1/SMAD3/USP19/SUMO1/BAG6/EP300/BAG3/TBRG1/USP33/WIZ/SEL1L |
| BP | GO:0016192/vesicle-mediated transport | 16 | 2.27E-03 | STX5/ARFGAP3/AP4E1/VPS41/AP4M1/CLTC/TSNARE1/RAB1A/COPG1/TSC2/GGA1/ARAP3/GOLGA4/VPS39/GGA3/AP3B1 |
| BP | GO:0000389/mRNA 3'-splice site recognition | 4 | 2.41E-03 | ISY1/SLU7/SF3A2/SF3A1 |
| BP | GO:1903146/regulation of mitophagy | 7 | 2.50E-03 | KAT2A/SREBF1/GSK3A/USP36/ATP13A2/SREBF2/HDAC6 |
| BP | GO:0006890/retrograde vesicle-mediated transport, Golgi to ER | 11 | 2.60E-03 | KIF2C/ARFGAP3/COPG1/SCYL1/KIFAP3/RAB1B/ARF5/KLC2/KDELR1/RAB1A/TAPBP |
| BP | GO:0006366/transcription from RNA polymerase II promoter | 37 | 2.64E-03 | CAMTA2/E2F4/ARID4A/ELF4/SPI1/FIGLA/HCFC1/ELK1/TBP/NFKB2/ELK3/SRF/TMF1/POU2F2/CREB3L2/MLLT1/TCEA1/RUNX1/PLAGL2/NFATC1/KAT2A/TAF4/TAF3/TAF6/FOXJ2/DTX1/LDB1/RELA/TP53/HMGA1/MNAT1/MEF2D/EP300/MNT/IRF2/TCEB1/NFIC |
| BP | GO:0045892/negative regulation of transcription, DNA-templated | 36 | 3.06E-03 | EID1/PPARD/ZNF552/TNF/ARID4A/RBM15B/SPI1/CBX4/PRAMEF20/ELK3/DAXX/CITED2/MEN1/SUMO1/NIPBL/POU2F1/GATAD2A/RARA/RUNX1/IKZF4/ZBTB7A/NACC1/SP100/LDB1/RELA/TP53/HDAC10/SIRT6/PHF12/HMGA1/HES1/PA2G4/SMARCC2/ZBTB4/SCMH1/HDAC6 |
| BP | GO:0006606/protein import into nucleus | 9 | 3.16E-03 | NUP214/TMCO6/IPO13/TSC2/PPP1R10/NUP188/PPP3CA/POM121C/KPNA3 |
| BP | GO:0016569/covalent chromatin modification | 13 | 3.32E-03 | UTP3/BRD2/BAG6/PHF1/BCORL1/SMARCB1/SMARCC2/SMARCD1/CBX4/ARID1A/BRD4/DAXX/CBX6 |
| BP | GO:0015031/protein transport | 30 | 3.50E-03 | WASH1/RAB5B/SNX14/VPS54/PTPN23/VPS52/RAB1B/SNX4/ARF5/HOOK3/TBC1D17/EXOC4/NECAP1/GPR89A/PIK3R2/RAB2A/GDI1/PGAP2/PRAF2/RSG1/ATG9A/SCAMP2/CORO7/AP4M1/PITPNM1/AAAS/CCDC53/DENND4C/RAP1A/COMMD1 |
| BP | GO:0006338/chromatin remodeling | 11 | 3.69E-03 | KAT2A/SMARCB1/SMARCC2/SMARCD1/NUDT5/ARID1A/BRD4/SCMH1/DAXX/BAZ2A/RERE |
| BP | GO:0006913/nucleocytoplasmic transport | 6 | 3.72E-03 | ANKRD54/AAAS/RBM15B/ANP32A/NSRP1/RGS14 |
| BP | GO:0006888/ER to Golgi vesicle-mediated transport | 16 | 3.72E-03 | RAB2A/STX5/ARFGAP3/DYNC1LI2/VAPB/RAB1B/RAB1A/COPG1/TRAPPC8/TRAPPC6B/USO1/CREB3L2/KDELR1/TEX261/SPAST/GOLGB1 |
| BP | GO:0007249/I-kappaB kinase/NF-kappaB signaling | 9 | 3.92E-03 | TNF/ROCK1/ROCK2/TICAM1/RIPK3/RBCK1/NFKB2/MAP3K14/NKIRAS2 |
| BP | GO:0016925/protein sumoylation | 13 | 4.41E-03 | SP100/CBX4/TP53/MTA1/NUP188/SUMO2/SUMO1/NUP214/AAAS/PIAS3/POM121C/SCMH1/HDAC7 |
| BP | GO:0006468/protein phosphorylation | 33 | 4.43E-03 | CDK18/STK36/MKNK2/MAP4K2/RPS6KB2/VRK1/HSF1/CLK3/COL4A3BP/RARA/CSK/CDK16/SIK3/PDK2/ROCK1/LIMK1/ROCK2/MAML1/MINK1/RPS6KC1/CDC25B/PRKD2/MNAT1/C19ORF35/ULK1/GSK3A/SCYL2/DYRK1B/NEK8/JAK3/CDK20/MAP3K12/MAP3K11 |
| BP | GO:0000165/MAPK cascade | 22 | 4.52E-03 | ZFP36/TNF/FGF7/MAP2K1/MAP2K2/MEN1/ZFP36L1/MEF2D/PSMF1/PSMD14/PSMC6/KRAS/PSMD12/PSMB1/ARAF/PSMC1/IL2RG/JAK3/MAP3K14/RASA4/PSMD7/DUSP7 |
| BP | GO:0007254/JNK cascade | 8 | 4.63E-03 | IRAK4/SH2D3C/TNF/PTGER4/MAP4K2/MINK1/MAP3K12/MAP3K11 |
| BP | GO:0006337/nucleosome disassembly | 5 | 5.10E-03 | SMARCB1/SMARCC2/SMARCD1/ARID1A/HMGA1 |
| BP | GO:0000381/regulation of alternative mRNA splicing, via spliceosome | 7 | 5.20E-03 | TRA2B/RBM15B/THRAP3/PTBP1/NSRP1/SF3A1/UHMK1 |
| BP | GO:0006511/ubiquitin-dependent protein catabolic process | 17 | 5.23E-03 | USP7/USP1/BAP1/USP19/PSMF1/PSMC6/UBE2D3/PSMD14/BAG6/USP21/USP36/SIAH1/TCEB1/USP25/USP42/USP33/FBXO11 |
| BP | GO:0007005/mitochondrion organization | 10 | 5.56E-03 | GABPB1/ALAS1/OPA1/VPS13C/AGTPBP1/ATP5B/PPRC1/HCFC1/YME1L1/CLUH |
| BP | GO:0045616/regulation of keratinocyte differentiation | 4 | 7.51E-03 | ZFP36/ZFP36L1/ROCK1/ROCK2 |
| BP | GO:0060674/placenta blood vessel development | 4 | 7.51E-03 | MAP2K1/PKD1/SPINT1/ARID1A |
| BP | GO:0006396/RNA processing | 11 | 8.63E-03 | HNRNPH3/EXOSC9/CHERP/HNRNPUL1/PNPT1/HNRNPD/DHX34/RBM39/SF3A1/XRN2/RTCA |
| BP | GO:0007264/small GTPase mediated signal transduction | 20 | 9.82E-03 | RAB2A/PLD2/GDI1/RSG1/RAB5B/RAB1B/ARF5/RAB1A/RRAGC/SH2D3C/ARL5A/KRAS/ARHGAP1/RAB11B/RHOBTB2/RAP1A/RIT1/RAP1B/NKIRAS2/RAB27A |
| BP | GO:0060534/trachea cartilage development | 3 | 1.04E-02 | RARG/RARA/SRF |
| BP | GO:1903347/negative regulation of bicellular tight junction assembly | 3 | 1.04E-02 | TNF/ROCK1/ROCK2 |
| BP | GO:0006461/protein complex assembly | 12 | 1.11E-02 | HES1/MNAT1/CD3E/KIFAP3/CREBBP/TP53/EPRS/KPNA3/HMGA1/WAS/CD74/TAPBP |
| BP | GO:0050852/T cell receptor signaling pathway | 14 | 1.12E-02 | CD3E/RELA/WAS/PRKD2/PSMF1/PSMC6/PSMD14/PSMD12/PSMB1/PSMC1/RBCK1/CSK/PSMD7/PIK3R2 |
| BP | GO:0008285/negative regulation of cell proliferation | 28 | 1.22E-02 | B4GALT1/CHERP/RARG/TAF6/MAP2K1/PTPN2/TP53/BAP1/ABI1/SIRT6/SKI/HMGA1/SRF/FTH1/MEN1/DDR1/RASSF5/TMEM127/HSF1/SMARCB1/KIFAP3/TSC2/TBRG1/MNT/RARA/NDRG1/CSK/TOB2 |
| BP | GO:0033209/tumor necrosis factor-mediated signaling pathway | 12 | 1.25E-02 | PSMF1/PSMD14/PSMC6/TNF/PSMD12/PSMB1/CDIP1/PSMC1/TNFRSF14/MAP3K14/PSMD7/LTA |
| BP | GO:0000209/protein polyubiquitination | 16 | 1.30E-02 | PPIL2/UBR2/PSMF1/PSMC6/UBE2D3/PSMD14/RNF115/PSMD12/PSMB1/FBXL8/PSMC1/RBCK1/SIAH1/PSMD7/TRIP12/HDAC6 |
| BP | GO:0016575/histone deacetylation | 7 | 1.32E-02 | ARID4A/ARID4B/HDAC10/MTA1/TBL1X/BAZ2A/HDAC6 |
| BP | GO:0006367/transcription initiation from RNA polymerase II promoter | 14 | 1.38E-02 | TAF4/PPARD/TAF3/RARG/TAF6/MAML1/CREBBP/MED25/MED12/MED24/TBP/MNAT1/THRAP3/RARA |
| BP | GO:0002223/stimulatory C-type lectin receptor signaling pathway | 11 | 1.46E-02 | PSMF1/PSMD14/PSMC6/KRAS/EP300/PSMD12/PSMB1/RELA/PSMC1/CREBBP/PSMD7 |
| BP | GO:0032092/positive regulation of protein binding | 8 | 1.51E-02 | MEN1/ACE/MFNG/SPAG8/EP300/TICAM1/PKD1/USP33 |
| BP | GO:0046777/protein autophosphorylation | 15 | 1.62E-02 | FLT4/MKNK2/MINK1/ATP13A2/UHMK1/PRKD2/DDR1/VRK1/CLK3/SLK/ULK1/RIPK3/CSK/MAP3K12/MAP3K11 |
| BP | GO:0051492/regulation of stress fiber assembly | 4 | 1.63E-02 | ROCK1/PTGER4/ROCK2/SYNPO |
| BP | GO:0000956/nuclear-transcribed mRNA catabolic process | 4 | 1.63E-02 | EXOSC9/THRAP3/DHX34/AGO1 |
| BP | GO:0045598/regulation of fat cell differentiation | 4 | 1.63E-02 | HES1/PPARD/ATAT1/HDAC6 |
| BP | GO:0035509/negative regulation of myosin-light-chain-phosphatase activity | 3 | 1.68E-02 | TNF/ROCK1/ROCK2 |
| BP | GO:0060071/Wnt signaling pathway, planar cell polarity pathway | 10 | 1.70E-02 | DVL3/PSMF1/PSMD14/PSMC6/PSMD12/PSMB1/PSMC1/MED12/CLTC/PSMD7 |
| BP | GO:0001843/neural tube closure | 9 | 1.71E-02 | KAT2A/RARG/TSC2/MED12/SPINT1/ARID1A/RARA/SKI/CITED2 |
| BP | GO:0002479/antigen processing and presentation of exogenous peptide antigen via MHC class I, TAP-dependent | 8 | 1.79E-02 | PSMF1/PSMD14/PSMC6/PSMD12/CD207/PSMB1/PSMC1/PSMD7 |
| BP | GO:0010506/regulation of autophagy | 7 | 1.94E-02 | HMGB1/EP300/ULK1/USP33/RRAGC/PIK3R2/HDAC6 |
| BP | GO:0032743/positive regulation of interleukin-2 production | 4 | 2.01E-02 | PRKD2/ANXA1/RUNX1/SASH3 |
| BP | GO:1901224/positive regulation of NIK/NF-kappaB signaling | 4 | 2.01E-02 | HAVCR2/HMGB1/TNF/AGO1 |
| BP | GO:1990126/retrograde transport, endosome to plasma membrane | 4 | 2.01E-02 | WASH1/RAB11B/VPS26A/VPS39 |
| BP | GO:0000380/alternative mRNA splicing, via spliceosome | 4 | 2.01E-02 | HNRNPM/PTBP1/SLU7/RBM17 |
| BP | GO:0000375/RNA splicing, via transesterification reactions | 5 | 2.07E-02 | BCAS2/TRA2B/SLU7/LSM1/SF3B4 |
| BP | GO:0006521/regulation of cellular amino acid metabolic process | 7 | 2.13E-02 | PSMF1/PSMD14/PSMC6/PSMD12/PSMB1/PSMC1/PSMD7 |
| BP | GO:0043123/positive regulation of I-kappaB kinase/NF-kappaB signaling | 14 | 2.13E-02 | TNF/SLC44A2/MTDH/RELA/UNC5CL/TERF2IP/IRAK4/ZDHHC17/TICAM1/RBCK1/PELI2/BRD4/MAP3K14/GPR89A |
| BP | GO:0006397/mRNA processing | 15 | 2.22E-02 | BCAS2/PDCD11/RBM15B/PTBP1/SF3A2/NSRP1/SF3A1/SF3B4/HNRNPA1/ZFP36L1/THRAP3/RBM39/LSM1/XRN2/KIAA1429 |
| BP | GO:0050790/regulation of catalytic activity | 8 | 2.25E-02 | BAG3/GPSM3/PCP2/SHOC2/DPM2/ATP6V1H/WAS/ARL2BP |
| BP | GO:0016579/protein deubiquitination | 10 | 2.62E-02 | USP7/USP19/OTUB1/USP1/USP21/BAP1/USP36/USP25/USP42/USP33 |
| BP | GO:0007507/heart development | 15 | 2.62E-02 | POU6F1/SMG9/PPARD/MAP2K1/MED12/SRF/CITED2/PTPN11/ZFP36L1/EIF4G2/EP300/TSC2/GYS1/NEK8/PKD1 |
| BP | GO:0006914/autophagy | 12 | 2.79E-02 | HMGB1/CLEC16A/ATG9A/TBC1D17/ATG5/MAP1S/ARSA/RAB1B/VPS41/VPS36/VPS39/RAB1A |
| BP | GO:0042147/retrograde transport, endosome to Golgi | 8 | 2.80E-02 | STX5/TBC1D17/WASH1/VPS54/VPS52/RGP1/CLTC/VPS26A |
| BP | GO:0042593/glucose homeostasis | 10 | 2.93E-02 | CRTC2/PDK2/PTPN2/WFS1/NCOA5/PLSCR3/SLC37A4/SIRT6/PFKM/PTPN11 |
| BP | GO:0031647/regulation of protein stability | 8 | 3.00E-02 | KAT2A/SREBF1/USP19/SUMO1/KRAS/DPM2/SUGT1/HDAC6 |
| BP | GO:0032007/negative regulation of TOR signaling | 5 | 3.03E-02 | TMEM127/AKT1S1/GSK3A/TSC2/UBR2 |
| BP | GO:0038066/p38MAPK cascade | 3 | 3.34E-02 | ZFP36/ZFP36L1/MAPK14 |
| BP | GO:1903140/regulation of establishment of endothelial barrier | 3 | 3.34E-02 | TNF/ROCK1/ROCK2 |
| BP | GO:0046627/negative regulation of insulin receptor signaling pathway | 5 | 3.40E-02 | PTPN2/GSK3A/RELA/NCOA5/TSC2 |
| BP | GO:0030033/microvillus assembly | 4 | 3.41E-02 | MINK1/RAP1A/RAP1B/FXYD5 |
| BP | GO:0051893/regulation of focal adhesion assembly | 4 | 3.41E-02 | ROCK1/SLK/ROCK2/LDB1 |
| BP | GO:0048010/vascular endothelial growth factor receptor signaling pathway | 8 | 3.43E-02 | PRKD2/ROCK1/ROCK2/MAPK14/FLT4/WASF2/ABI1/PIK3R2 |
| BP | GO:0007420/brain development | 15 | 3.45E-02 | UTP3/POU6F1/SMG9/STK36/STXBP3/ZNF335/PTPN11/MEN1/SLC17A7/PITPNM1/BAG6/NIPBL/MAP1S/BAG3/CSK |
| BP | GO:0070301/cellular response to hydrogen peroxide | 7 | 3.46E-02 | IL18BP/HSF1/RELA/ANXA1/ZNF277/KDM6B/HDAC6 |
| BP | GO:0030155/regulation of cell adhesion | 6 | 3.59E-02 | ROCK1/ROCK2/PKD1/FAF1/ENG/SRF |
| BP | GO:0006464/cellular protein modification process | 10 | 3.64E-02 | UBE2D3/ST8SIA4/ARAF/RIPK3/BAP1/DUSP12/USP25/FBXO11/TTLL3/RABGGTA |
| BP | GO:0006283/transcription-coupled nucleotide-excision repair | 8 | 3.90E-02 | HMGN1/USP7/MNAT1/EP300/RFC1/ISY1/COPS7B/TCEA1 |
| BP | GO:0007004/telomere maintenance via telomerase | 4 | 3.96E-02 | ACD/RFC1/SMG7/TERF2IP |
| BP | GO:0031293/membrane protein intracellular domain proteolysis | 4 | 3.96E-02 | HM13/RELA/SPPL2B/SPPL2A |
| BP | GO:0060324/face development | 4 | 3.96E-02 | RARG/MAP2K1/RARA/SRF |
| BP | GO:0051726/regulation of cell cycle | 11 | 4.06E-02 | DOT1L/NUP214/EP300/CDK18/CCNI/TSC2/CCNF/MNT/BAP1/CDK16/CDK20 |
| BP | GO:0034976/response to endoplasmic reticulum stress | 8 | 4.15E-02 | USP19/TMX1/WFS1/COL4A3BP/PPP2CB/CREB3L2/TMEM259/PIK3R2 |
| BP | GO:0048147/negative regulation of fibroblast proliferation | 5 | 4.21E-02 | TP53/MED25/SKI/FTH1/LTA |
| BP | GO:0042771/intrinsic apoptotic signaling pathway in response to DNA damage by p53 class mediator | 5 | 4.21E-02 | PGAP2/BAG6/EP300/CDIP1/TP53 |
| BP | GO:0030968/endoplasmic reticulum unfolded protein response | 6 | 4.25E-02 | SYVN1/VAPB/WFS1/ATF6B/CREB3L2/FGF21 |
| BP | GO:2000774/positive regulation of cellular senescence | 3 | 4.33E-02 | KRAS/YPEL3/HMGA1 |
| BP | GO:0000288/nuclear-transcribed mRNA catabolic process, deadenylation-dependent decay | 3 | 4.33E-02 | ZFP36/ZFP36L1/CNOT3 |
| BP | GO:0046825/regulation of protein export from nucleus | 3 | 4.33E-02 | XPO1/UHMK1/PTPN11 |
| BP | GO:0030220/platelet formation | 4 | 4.56E-02 | EP300/SRF/ZNF385A/PTPN11 |
| BP | GO:0008286/insulin receptor signaling pathway | 8 | 4.96E-02 | SREBF1/PDK2/EIF4EBP2/PTPN2/GSK3A/ATP6V1H/ATP6V1D/PIK3R2 |
| BP | GO:0045600/positive regulation of fat cell differentiation | 6 | 4.98E-02 | ZFP36/ASXL2/ZFP36L1/PPARD/CARM1/ZNF385A |
| CC | GO:0005654/nucleoplasm | 216 | 1.13E-21 | HMGN1/PTPN23/MED25/MED24/INTS1/CLK3/COL4A3BP/CREB3L2/RARA/RPP25/CLNS1A/RARG/OPA1/PTBP1/MED12/UBR2/HES1/PPP1CA/AAAS/RFC1/PIAS3/YME1L1/CRTC2/HNRNPA3/VRK1/ACD/ATN1/POLM/ISY1/SLC4A1AP/ASXL2/IKZF4/MAML1/CREBBP/DDX1/SMAD3/SKI/HNRNPA1/SRSF3/RNF7/PPRC1/ZNF385A/ENG/PPARD/ELF4/SHOC2/TERF2IP/NFKB2/DAXX/C19ORF47/BAG6/HSF1/ANKRD12/ANP32A/BRD4/GOLGA4/TOMM34/SYMPK/STX5/EXOSC9/MBD6/TP53/PAPD7/TAF15/TXN/USP21/RBM39/KIAA1429/USP7/MKNK2/SF3B4/SRRT/SUMO2/NUP214/SUMO1/GATAD2A/AGO1/MLLT1/TCEA1/PPP3CA/USP33/BAZ2A/TRIP12/BCAS2/PDK2/PPP1R10/ELAVL1/PHF12/SF3A2/SF3A1/DOT1L/MNAT1/MEF2D/PSMD14/HNRNPUL2/PSMD12/BCORL1/HNRNPUL1/MAPK14/TCEB1/DUSP7/XPO1/RBM15B/CBX4/RPS6KB2/TBP/CBX6/GABPB1/DNAJC7/CTDSP1/MLST8/SCAMP2/RELA/USP1/HDAC10/MTA1/ARID1A/PA2G4/FAM222B/EP300/SLU7/SNRPE/XRN2/GPN1/EID1/HMGB1/SYVN1/SETD1B/PPIL2/ELK1/COPS7B/TRRAP/ELK3/SRF/HNRNPM/UBE2D3/NIPBL/AKT1S1/PSMB1/SMARCB1/HNRNPD/TMEM192/RUNX1/THAP11/SREBF1/TAF4/TAF3/TAF6/PTPN2/ZMYM3/GINS3/SPPL2B/SREBF2/HNRNPH3/PSMC6/PHF2/PHF1/ATF7/SMARCC2/PSMC1/YIPF3/SCMH1/HDAC7/RAI1/E2F4/FOSL2/ARID4A/ARID4B/BAP1/FAM63B/LARP1/MEN1/ALAS1/PSMD7/ELMSAN1/SP100/LIMK1/HMGA1/PRKD2/ALDH1B1/ZMIZ2/ZMIZ1/CPSF7/COMMD1/CARM1/KPNA3/KDM6B/UTP3/DPF2/KMT2D/XIAP/TRA2B/KMT2B/HCFC1/PSMF1/CENPA/POU2F2/POU2F1/RTCA/NFATC1/KAT2A/PDCD11/RSG1/ANXA1/SAMHD1/FAM188A/SIRT6/SMC2/CDC25B/SMC4/RNF44/THRAP3/IRF2/TBL1X/GGA1/KDM4D |
| CC | GO:0005634/nucleus | 332 | 1.27E-17 | HMGN1/PRR13/PTPN23/MED24/NSRP1/CITED2/CLK3/DHX34/TIGD2/CREB3L2/STAG3/RARA/FAM103A1/LSM1/CLNS1A/RARG/ZNF100/ROCK2/PTBP1/MED12/HES1/PPP1CA/SPAG8/AAAS/SCYL1/RFC1/SERBP1/TBRG1/MNT/CRTC2/RAD23A/ZNF76/FIGLA/PRRC2A/DUSP12/UHMK1/MANF/DTD1/HNRNPA3/VRK1/ACD/ATN1/POLM/SLC4A1AP/OLFM2/IQSEC1/PLAGL2/FRA10AC1/IKZF4/SRP54/UFSP2/MAP2K1/MAP2K2/MAML1/FAM111A/CREBBP/DDX1/SMAD3/PCNP/SKI/ZBTB45/HNRNPA1/VDAC3/PTPN11/RNF7/PPRC1/RERE/SASH3/ATAD2B/RANBP10/PPARD/KIAA0368/ELF4/ATP5B/SHOC2/MLH3/NFKB2/TERF2IP/DAXX/ARL2BP/BAG6/HSF1/ANP32A/SPIB/BRD4/DXO/SERTAD2/TOMM34/ZFP36/PGAP2/TMCO6/EXOSC9/CCNF/MBD6/PAPD7/TP53/ZNF687/ZNF335/TAF15/NAB2/TXN/PFDN4/ZNF277/MGEA5/SIAH1/RBM39/USP25/SRCAP/PARVA/TOP3B/USP7/RBM34/PTOV1/MTDH/ZNF552/MKNK2/BCCIP/ABI1/CIC/SEC14L2/ZFP36L1/SUMO2/SORBS3/SUMO1/NUP214/ZSCAN22/PPP2CB/REXO1/GATAD2A/AGO1/MLLT1/PKD1/AATF/TCEA1/USP36/PPP3CA/MLLT6/BAZ2A/TRIP12/ZNF564/NEMF/RAB2A/BCAS2/BRD2/UPF2/RRP15/CS/ELAVL1/PPP1R10/PHF12/SF3A2/NAE1/DOT1L/RLF/MNAT1/MEF2D/PSMD14/RASSF5/ZNF672/HNRNPUL2/HNRNPUL1/MAPK14/SYF2/ZBTB4/ZNF764/APBB3/TOB2/XPO1/PLXNA3/STK36/EIF5/CNOT3/TCEAL6/CBX4/RPS6KB2/TBP/TCEAL8/CBX6/GABPB1/ANKRD54/MIER2/ATF6B/CTDSP1/IFRD2/SAMD4B/RELA/CHTF8/USP1/NUDT5/HDAC10/MTA1/ARID1A/PA2G4/EP300/SLU7/DNTTIP2/SNRPE/XRN2/FBXO11/ZC3H3/EID1/HMGB1/CAMTA2/SSH3/SETD1B/PPIL2/ELK1/RABGAP1L/ELK3/TRRAP/SRF/FTH1/RRAGC/IRAK4/NIPBL/DDX46/PSMB1/SMARCB1/RPL9/TMA16/HNRNPD/TMEM192/NDRG1/SSX2IP/RUNX1/ARHGDIA/SREBF1/TAF3/TAF6/PTPN2/MICU2/GINS3/SMG7/ABHD14B/TSN/SUGT1/SREBF2/ATE1/HNRNPH3/PSMC6/PHF2/R3HDM2/PHF1/OTUB1/DYRK1B/ATF7/TSC2/PSMC1/SCMH1/HDAC7/HDAC6/HINT3/POU6F1/RAI1/AEBP1/FOSL2/E2F4/ARID4A/AGTPBP1/LYAR/ARID4B/BAP1/TMF1/MEN1/KIF2C/CDIP1/ZYX/PSMD7/ELMSAN1/SP100/CPTP/SDR39U1/FOXJ2/NOL8/LDB1/MID1IP1/HMGA1/NLRP1/PRKD2/TRNAU1AP/FAM120A/ZMIZ2/NCOA5/CPSF7/COMMD1/CARM1/KPNA3/COMMD6/SPAST/KDM6B/EPN1/WIZ/UTP3/KMT2D/XIAP/TRA2B/KMT2B/HCFC1/NPAS1/TSC22D2/CENPA/POU2F2/POU2F1/MLXIP/RSL24D1/VPS36/RTCA/PIK3R2/NFATC1/SYNPO/ALKBH6/KAT2A/ZBTB7A/NACC1/PDCD11/DTX1/FLT4/CBL/ANXA1/SAMHD1/SIRT6/SMC2/ANXA4/RGS14/SMC4/MAP1S/THRAP3/FAF1/NFIC/TBL1X/GGNBP2/CDK20 |
| CC | GO:0005737/cytoplasm | 290 | 3.21E-09 | KIFC2/HMGN1/VAPB/SRP68/PTPN23/HOOK3/CITED2/VPS13C/RARA/LSM1/CLNS1A/OPA1/UNC5CL/UBR2/HES1/PITPNM1/PPP1CA/SPAG8/AAAS/SCYL1/NAV1/RFC1/PIAS3/SERBP1/NEK8/CRTC2/OSTF1/DUSP12/PRRC2A/ARF5/NAA35/DTD1/TPGS2/HNRNPA3/FAM65A/CDC42EP2/VRK1/KRAS/ACD/TMEM127/ATN1/SPATS2/SLC4A1AP/OLFM2/IQSEC1/GIT1/SRP54/UFSP2/MAP2K1/C15ORF39/MAP2K2/FAM111A/CREBBP/DDX1/EPRS/SMAD3/SKI/HNRNPA1/PTPN11/SRSF3/SYNE4/RNF7/ZNF385A/ENG/SASH3/TTC25/SHOC2/NFKB2/TERF2IP/KCNIP2/DAXX/TTLL3/BAG6/HSF1/ANKRD12/BAG3/ANP32A/SPG20/SPIB/BRD4/DXO/GOLGA4/SIK3/SERTAD2/TOMM34/ZFP36/SYMPK/TMCO6/EXOSC9/PAPD7/TP53/ZNF687/PI4KB/ZDHHC17/TAF15/BNIP2/TXN/PFDN4/USP21/USH1C/SIAH1/USP25/MAP3K14/MAP3K12/MAP3K11/PARVA/MTDH/MKNK2/ABI1/SEC14L2/PFAS/PLCL2/ZFP36L1/SRRT/SUMO1/ATXN2L/MMACHC/PPP2CB/EXOC4/FASN/AGO1/CSDE1/MLLT1/PKD1/AATF/UNK/GK5/PPP3CA/USP33/BAZ2A/TRIP12/FAM89B/GDI1/PDK2/BRD2/UPF2/IPO13/AMACR/ELAVL1/NAE1/MNAT1/MEF2D/RASSF5/RPL23/PSMD12/BCORL1/MAPK14/APBB3/UBXN4/TOB2/XPO1/CHERP/STK36/EIF5/RPS6KB2/TBP/TBC1D15/PACSIN1/ANKRD54/EIF4EBP2/NUDCD2/ATAT1/ATG5/DNAJC7/MLST8/SAMD4B/RELA/HDAC10/MTA1/LYPLA2/RPS6KC1/ANKRD13D/PA2G4/EP300/SLU7/FLAD1/FBXO11/GPN1/EID1/ARFGAP3/TMEM214/SSH3/SETD1B/PPIL2/PNPT1/SNX4/COPS7B/SRF/FTH1/RRAGC/IRAK4/AKT1S1/PSMB1/RPL9/GMPPA/NDRG1/ST13P5/RUNX1/THAP11/ARHGDIA/SREBF1/B4GALT3/TAF4/TAF6/ZMYM3/SMG7/MEA1/ARMC5/ABHD14B/SREBF2/ATE1/PHF2/PHF1/ULK1/OTUB1/SLAIN2/ATF7/TSC2/PSMC1/RAP1A/HDAC7/HDAC6/CYB5R3/HINT3/AEBP1/AGTPBP1/ARID4B/GABBR1/BAP1/LARP1/MEN1/ALAS1/SLK/ARHGAP1/ZYX/CSK/CDK16/VCPIP1/SP100/CPTP/LIMK1/NOL8/MINK1/CLUH/PFKM/TMEM189/PRKD2/EML3/SH2D3C/TRNAU1AP/FAM120A/ZMIZ1/COMMD1/MAPRE1/CARM1/KPNA3/COMMD6/SPAST/NKIRAS2/EPN1/DPF2/XIAP/GPSM3/HCFC1/TSC22D2/POU2F2/NFATC1/ALKBH6/NACC1/RSG1/TMC8/DTX1/FLT4/CBL/ANXA1/SIRT6/SMC2/ANXA4/RGS14/CDC25B/SMC4/SH3BP5/RNF44/MAP1S/IRF2/TMEM86B/ARAP3/SH3BP1/GGNBP2/CDK20 |
| CC | GO:0005829/cytosol | 198 | 2.19E-08 | DYNC1LI2/A2M/VPS54/SRP68/VPS52/RAB1A/HOOK3/VPS13C/BRPF3/KIFAP3/COL4A3BP/PELI2/LSM1/CDCA3/CLNS1A/ROCK1/ROCK2/UBR2/VPS41/AP4M1/PPP1CA/FBXL8/DUSP12/VRK1/CDC42EP2/KRAS/GYS1/GIT1/DVL3/SRP54/MAP2K1/MAP2K2/EPRS/SMAD3/DENND1C/PTPN11/RPE/RANBP10/WASF2/NFKB2/DAXX/ARL2BP/USP19/BAG6/HSF1/TRAPPC6B/BAG3/TICAM1/DPP7/GOLGA4/ZFP36/STX5/EXOSC9/TP53/PI4KB/WAS/BNIP2/EIF4A2/PFDN4/TXN/MGEA5/USH1C/SIAH1/WDR44/MAP3K14/MAP3K12/TOP3B/PARVA/USP7/MIDN/WASH1/ABI1/KLC2/PFAS/ZFP36L1/NUP214/SORBS3/MMACHC/PPP2CB/EXOC4/FASN/AGO1/ABCD3/PPP3CA/RASA4/TRIP12/GDI1/UPF2/ELAVL1/NAE1/PSMD14/RPL23/PSMD12/GSK3A/MAPK14/ARAF/TCEB1/FAM126B/DUSP7/XPO1/EIF5/CNOT3/PACSIN1/TBC1D17/ATAT1/SCLY/ATG5/SPRED2/DNAJC7/RBCK1/MLST8/RELA/NUDT5/CORO7/COPG1/RIPK3/USO1/FLAD1/SNRPE/VPS26A/ARFGAP3/SRM/FTH1/RRAGC/IRAK4/UBE2D3/AKT1S1/PSMB1/RPL9/HNRNPD/RHOBTB2/NDRG1/ARHGDIA/SREBF1/SMG9/CKAP5/SMG7/FN3KRP/ABHD14B/TSN/SUGT1/AK8/SREBF2/PSMC6/ULK1/PSMC1/TSC2/RAP1A/RAP1B/HDAC6/AGTPBP1/RGP1/CLTC/TMF1/MEN1/KIF2C/ARHGAP1/CSK/PSMD7/PLCB2/CPTP/LIMK1/MINK1/ATP6V1H/STXBP3/MID1IP1/PFKM/HMGA1/ATP6V1D/NLRP1/EIF4G2/MAPRE1/KPNA3/CARM1/XIAP/PPM1B/SEC62/SEC63/PSMF1/MTHFR/CENPA/VPS36/NFATC1/PIK3R2/PDCD11/DTX1/CBL/TPMT/SMC2/SMC4/CDC25B/RNF115/MAP1S/DENND4C/IRF2/JAK3/FAF1/ARAP3 |
| CC | GO:0016020/membrane | 141 | 9.08E-08 | XPO1/PLXNA3/DYNC1LI2/CHERP/HM13/VPS52/INTS1/TSNARE1/TAPBP/CLK3/AGPS/ATG5/PGRMC2/DHX34/SPRED2/DNAJC7/CFI/ATG9A/OPA1/PTBP1/MED12/UNC5CL/RPS6KC1/CORO7/VPS41/PITPNM1/SPAG8/IGSF8/CD37/PA2G4/AAAS/LRP10/SCYL1/SERBP1/ACAP2/USO1/SLU7/YME1L1/XRN2/MFNG/ARFGAP3/SYVN1/PNPT1/BROX/PRRC2A/SNX4/FXYD5/CD74/HNRNPM/ACE/CDC42EP2/KRAS/DDX46/RPL9/GYS1/ZMPSTE24/IQSEC1/GIT1/B4GALT1/HPCAL1/CKAP5/SPPL2B/DDX1/EPRS/SPPL2A/ATP13A3/HNRNPA1/SREBF2/PSMC6/PSMC1/TSC2/RAP1A/RIT1/RAP1B/CYB5R3/KIAA0368/RAB5B/ATP5B/NUP188/CHPF2/RGP1/CLTC/CANX/LARP1/KIF2C/PIGK/BAG6/NECAP1/KDELR1/PSMD7/LBR/LTA/AP3B1/TOMM34/STX5/PCYOX1L/LIMK1/CCDC47/SPINT1/ATP6V1D/EIF4G2/DDR1/SH2D3C/FAM120A/CPSF7/MGEA5/SEMA4B/C16ORF58/NKIRAS2/MAP3K12/MAP3K11/GALNT1/TNF/GALNT7/LMF2/SLC37A4/HCFC1/PPM1B/KLC2/SEC62/SEC63/PSMF1/ATXN2L/FASN/EXOC4/ABCD3/IL2RG/PPP3CA/SELPLG/DLGAP4/ELAVL1/CYP20A1/CLPTM1/LSP1/HNRNPUL2/PSMD12/RPL23/TMEM86B/JAK3/GGA1/GOLGB1 |
| CC | GO:0000790/nuclear chromatin | 24 | 5.20E-06 | KAT2A/DPF2/DVL3/TAF4/PPARD/RARG/E2F4/LDB1/RELA/CREBBP/TP53/SPI1/SMAD3/ARID1A/TBP/SRF/WBP2/CITED2/MEN1/SMARCB1/SMARCC2/RARA/ZNF385A/NFATC1 |
| CC | GO:0016604/nuclear body | 9 | 4.58E-05 | USP7/SUMO1/NACC1/MTDH/CREBBP/TP53/CBX4/SKI/RGS14 |
| CC | GO:0005730/nucleolus | 60 | 9.00E-05 | XPO1/LYAR/RBM15B/SRP68/DAXX/AGPS/EXOSC9/SP100/CPTP/FOXJ2/NOL8/PTBP1/TP53/MBD6/MED12/DNAJC21/PA2G4/PPP1CA/NOP2/TAF15/RFC1/LLPH/USO1/NOL10/DNTTIP2/XRN2/FBXO11/UTP3/TMX1/MIDN/RBM34/MTDH/YPEL3/MKNK2/HNRNPM/VRK1/SUMO1/SMARCB1/RPL9/TMA16/MLLT1/AATF/TCEA1/USP36/RSL24D1/BAZ2A/BCAS2/SRP54/PDCD11/NIN/RRP15/ABHD14B/SIRT6/SMC2/PHF2/RPL23/MAP1S/GPATCH2/NFIC/ZNF385A |
| CC | GO:0005794/Golgi apparatus | 60 | 1.07E-04 | FGF7/ELF4/VAPB/VPS54/VPS52/RAB1B/RAB1A/HOOK3/TMF1/ST6GALNAC4/ATAT1/PAK4/KIFAP3/COL4A3BP/ATF6B/DPP7/GOLGA4/RAB27A/AP3B1/STX5/PLD2/SCAMP2/CPTP/MINK1/CORO7/ST6GALNAC1/PRKD2/ZDHHC17/COPG1/SCYL1/SCYL2/BACE2/USO1/MAPRE1/WDR44/SRCAP/ARFGAP3/GALNT1/TMEM214/RABGAP1L/ZDHHC18/ARF5/TRRAP/FASN/CSDE1/AATF/TMEM192/FKRP/USP33/RAB2A/B4GALT1/GDI1/B4GALT3/TMC8/MAP2K1/MAP2K2/TSC2/YIPF3/GGA1/GOLGB1 |
| CC | GO:0005913/cell-cell adherens junction | 29 | 1.91E-04 | VAPB/EIF5/WASF2/HCFC1/ABI1/KLC2/RAB1A/LARP1/ATXN2L/SLK/BAG3/PAK4/ARHGAP1/FASN/RAB11B/NDRG1/ZYX/SSX2IP/STX5/CKAP5/CBL/ANXA1/LYPLA2/EIF4G2/SCYL1/SERBP1/USO1/MAPRE1/PARVA |
| CC | GO:0000139/Golgi membrane | 43 | 4.66E-04 | GALNT1/ARFGAP3/GALNT7/NDST2/VAPB/MAP4K2/RAB1B/CHPF2/RGP1/CD74/RAB1A/TAPBP/TMF1/SLC35A4/B3GNT9/POMGNT1/ST6GALNAC4/TRAPPC6B/PKD1/GXYLT1/KDELR1/GOLGA4/FKRP/GPR89A/RAB2A/B4GALT1/SREBF1/PGAP2/STX5/B4GALT3/ROCK1/SPPL2B/CORO7/PI4KB/RHBDD2/SREBF2/ST6GALNAC1/MAN2A2/ZDHHC17/COPG1/ST8SIA4/USO1/GOLGB1 |
| CC | GO:0000502/proteasome complex | 10 | 7.54E-04 | PSMF1/PSMD14/PSMC6/PSMD12/PSMB1/KIAA0368/WFS1/RAD23A/PSMC1/PSMD7 |
| CC | GO:0005769/early endosome | 21 | 1.37E-03 | HAVCR2/HMGB1/WASH1/KIAA0368/RAB5B/MAP2K1/MAP2K2/WASF2/PTPN23/RABGAP1L/ACKR2/RPS6KC1/VPS41/RAB1A/TMEM127/CCDC53/RAP1A/COMMD1/SIAH1/VPS26A/RIN3 |
| CC | GO:0030660/Golgi-associated vesicle membrane | 6 | 1.40E-03 | ZDHHC17/HM13/SPPL2B/PKD1/SPPL2A/GPR89A |
| CC | GO:0016607/nuclear speck | 19 | 1.77E-03 | SRP54/MAML1/SETD1B/CBX4/SF3A2/NSRP1/SRSF3/SUMO1/DDX46/ATXN2L/CLK3/PIAS3/ZMIZ1/THRAP3/GATAD2A/GPATCH2/SLU7/RBM39/KIAA1429 |
| CC | GO:0016605/PML body | 12 | 2.40E-03 | USP7/SUMO2/SUMO1/SP100/HSF1/ELF4/MKNK2/TP53/SKI/LRCH4/DAXX/RGS14 |
| CC | GO:0030529/intracellular ribonucleoprotein complex | 14 | 4.23E-03 | ZFP36/XPO1/DDX1/EPRS/NSRP1/HNRNPA1/HNRNPA3/ZFP36L1/HNRNPM/PA2G4/HNRNPH3/HNRNPUL1/HNRNPD/AGO1 |
| CC | GO:0010008/endosome membrane | 17 | 4.38E-03 | PRAF2/CPTP/PLEKHM2/SPPL2B/VPS52/VPS41/IRAK4/TMEM175/UBE2D3/SCYL2/TICAM1/ACAP2/COMMD1/GGA1/WDR44/VPS26A/GGA3 |
| CC | GO:0015630/microtubule cytoskeleton | 14 | 4.50E-03 | CKAP5/VPS41/MID1IP1/TTLL3/KIF2C/EML3/CDC42EP2/NUDCD2/KIFAP3/SLAIN2/NDRG1/RBM39/CDK16/SPAST |
| CC | GO:0019013/viral nucleocapsid | 6 | 5.33E-03 | HNRNPA3/HNRNPM/HNRNPH3/HNRNPUL1/HNRNPD/HNRNPA1 |
| CC | GO:0071556/integral component of lumenal side of endoplasmic reticulum membrane | 6 | 6.22E-03 | HM13/SPPL2B/SPPL2A/CANX/CD74/TAPBP |
| CC | GO:0043231/intracellular membrane-bounded organelle | 36 | 9.67E-03 | DPF2/XPO1/PLXNA3/SLC37A4/PRRC2A/ELK3/CLTC/RRAGC/PECR/NUP214/BAG6/AGPS/POU2F1/ABCD3/SLC4A1AP/TMEM192/RUNX1/DPP7/GOLGA4/SCAMP2/MAML1/RPS6KC1/SPPL2A/MTA1/ATP13A3/MEF2D/ZDHHC17/BNIP2/ALDH1B1/ZMIZ1/CCDC53/TBRG1/YIPF3/SLU7/GGA1/VPS26A |
| CC | GO:0048471/perinuclear region of cytoplasm | 39 | 1.08E-02 | PTOV1/GALNT1/MTDH/CHERP/TRA2B/VPS54/VPS52/ARF5/MANF/PSMF1/PACSIN1/SLK/ATN1/COL4A3BP/ARHGAP1/ANP32A/NDRG1/RARA/TMEM192/USP33/UPF2/MAP2K2/CBL/PI4KB/ANXA4/RHBDD2/BNIP2/MAP1S/SCYL2/SERBP1/EIF4A2/TSC2/USO1/RAP1A/FAF1/WDR44/SRCAP/SPAST/HDAC6 |
| CC | GO:0016235/aggresome | 6 | 1.09E-02 | GIT1/EID1/RAB11B/SEC62/XRN2/HDAC6 |
| CC | GO:0005622/intracellular | 73 | 1.14E-02 | XPO1/RAB5B/WASF2/BAP1/RAB1B/CLTC/RAB1A/TBC1D15/ARL5A/NUDCD2/EIF4EBP2/ATF6B/MOB3A/PLCB2/RAB27A/STX5/PLD2/ZNF100/PTGER4/ROCK1/PLXNB1/ROCK2/NUDT5/VPS41/PI4KB/NLRP1/MAST3/SH2D3C/PITPNM1/PRKD2/NKIRAS2/MAP3K12/MAP3K11/OSTF1/ZNF552/PI4KAP2/MAP4K2/PI4KAP1/ABI1/ARF5/SEC14L2/CD74/SEC63/TBC1D3B/PLCL2/IRAK4/KRAS/RHOBTB2/RAB11B/IL2RG/POM121C/RASA4/VPS39/RAB2A/GDI1/DVL3/SMG9/SRP54/IPO13/SMAD3/SAMHD1/RGS14/CDC25B/SH3BP5/RASSF5/MAPK14/ARAF/RAP1A/RIT1/RAP1B/ZNF764/GGA1/FAM126B |
| CC | GO:0071564/npBAF complex | 4 | 1.17E-02 | SMARCB1/SMARCC2/SMARCD1/ARID1A |
| CC | GO:0005770/late endosome | 12 | 1.24E-02 | ANKRD13D/ATG9A/KIAA0368/WASH1/MAP2K1/SNX14/MAP2K2/RAP1A/SPPL2A/TMEM192/ATP13A2/RAB27A |
| CC | GO:0035097/histone methyltransferase complex | 5 | 1.37E-02 | MEN1/KMT2D/SETD1B/KMT2B/ZNF335 |
| CC | GO:0071013/catalytic step 2 spliceosome | 10 | 1.38E-02 | BCAS2/HNRNPA3/HNRNPM/ISY1/SYF2/SLU7/SF3A2/SNRPE/HNRNPA1/SF3A1 |
| CC | GO:0000118/histone deacetylase complex | 6 | 1.56E-02 | HDAC10/TBL1X/RERE/HDAC7/ELMSAN1/HDAC6 |
| CC | GO:0005669/transcription factor TFIID complex | 6 | 1.56E-02 | TAF4/TAF3/TAF6/TP53/TBP/TCEA1 |
| CC | GO:0097165/nuclear stress granule | 3 | 1.57E-02 | SYMPK/SUMO1/HSF1 |
| CC | GO:0005681/spliceosomal complex | 10 | 1.57E-02 | BCAS2/HNRNPM/HNRNPH3/SLU7/SF3A2/SNRPE/SF3B4/HNRNPA1/SF3A1/RBM17 |
| CC | GO:0071565/nBAF complex | 4 | 1.82E-02 | SMARCB1/SMARCC2/SMARCD1/ARID1A |
| CC | GO:0033276/transcription factor TFTC complex | 4 | 1.82E-02 | KAT2A/TAF4/TAF6/TRRAP |
| CC | GO:0016514/SWI/SNF complex | 4 | 2.21E-02 | SMARCB1/SMARCC2/SMARCD1/ARID1A |
| CC | GO:0071458/integral component of cytoplasmic side of endoplasmic reticulum membrane | 3 | 2.30E-02 | HM13/SPPL2B/SPPL2A |
| CC | GO:0071012/catalytic step 1 spliceosome | 3 | 2.30E-02 | BCAS2/ISY1/SYF2 |
| CC | GO:0030131/clathrin adaptor complex | 4 | 2.64E-02 | AP4M1/GGA1/GGA3/AP3B1 |
| CC | GO:0005801/cis-Golgi network | 6 | 2.87E-02 | SCYL1/YIPF6/TRAPPC6B/KDELR1/GOLGB1/HOOK3 |
| CC | GO:0035371/microtubule plus-end | 4 | 3.11E-02 | KIF2C/CKAP5/SLAIN2/MAPRE1 |
| CC | GO:0022624/proteasome accessory complex | 4 | 3.11E-02 | PSMD14/PSMC6/PSMD12/PSMC1 |
| CC | GO:0031931/TORC1 complex | 3 | 3.13E-02 | AKT1S1/MLST8/LARP1 |
| CC | GO:0005874/microtubule | 21 | 3.45E-02 | KIFC2/DYNC1LI2/MAP2K2/MTA1/MID1IP1/KLC2/RGS14/TTLL3/HOOK3/TPGS2/EML3/KIF2C/RASSF5/ATAT1/NAV1/MAP1S/NDRG1/MAPRE1/SPAST/HDAC6/MAP3K11 |
| CC | GO:0005783/endoplasmic reticulum | 46 | 3.80E-02 | CYB5R3/TMX1/PGS1/MTDH/SYVN1/HM13/KIAA0368/TMEM214/VAPB/WFS1/SLC37A4/SRP68/ABI1/SEC62/CANX/SEC63/MANF/TAPBP/TMF1/PSMF1/TRAPPC6B/KIFAP3/ANP32A/POU2F1/CREB3L2/TMEM192/KDELR1/VCPIP1/SREBF1/TMC8/UFSP2/YIPF6/MAP2K1/PTPN2/MAP2K2/TP53/CCDC47/SREBF2/BACE2/USO1/SPCS3/SUMF2/USP25/UBXN4/SPAST/SEL1L |
| CC | GO:0031595/nuclear proteasome complex | 3 | 4.06E-02 | PSMC6/PSMD12/PSMC1 |
| CC | GO:0005686/U2 snRNP | 4 | 4.76E-02 | CCDC97/SF3A2/SNRPE/SF3A1 |
| CC | GO:0005776/autophagosome | 7 | 4.85E-02 | TBC1D17/ATG5/WASH1/ULK1/TICAM1/ATP13A2/USP33 |
| MF | GO:0005515/protein binding | 522 | 1.86E-27 | A2M/PLEKHM2/VPS54/VPS52/MED25/MED24/NSRP1/CITED2/CLK3/BRPF3/LSM1/GNG4/RAB27A/CDCA3/OPA1/ROCK1/ROCK2/PTBP1/MED12/VPS41/PITPNM1/SPAG8/PPP1CA/TRAPPC8/SCYL2/CRTC2/OSTF1/RAD23A/PRRC2A/DUSP12/ARF5/NAA35/CDC42EP2/ATN1/ISY1/IQSEC1/FRA10AC1/GIT1/DVL3/MAP2K1/MAP2K2/FOXRED2/PCNP/SRSF3/SYNE4/RERE/FGF7/TTC25/ELF4/SPI1/PCED1A/DAXX/C19ORF47/USP19/POMGNT1/C19ORF44/HSF1/TICAM1/SPG20/ANP32A/BRD4/SIK3/PGAP2/SYMPK/STX5/TMCO6/EXOSC9/TP53/ZNF687/MAPK1IP1L/LRRC29/PI4KB/WAS/ZNF335/MAST3/TAF15/BNIP2/EIF4A2/NAB2/PFDN4/TXN/USP21/USH1C/USP25/PARVA/MKNK2/ABI1/BCCIP/SF3B4/SRRT/SORBS3/NUP214/ZSCAN22/MMACHC/PPP2CB/CSDE1/EXOC4/RAB11B/PKD1/LIMD2/TCEA1/PPP3CA/USP33/TRIP12/ZNF564/UPF2/BRD2/ELAVL1/PHF12/SF3A2/SF3A1/NAE1/DOT1L/RLF/MEF2D/MNAT1/KIAA1143/RASSF5/RPL23/GSK3A/HNRNPUL1/ARAF/PLSCR3/SYF2/ZBTB4/DPM2/ZNF764/TCEB1/USP42/DUSP7/XPO1/CHERP/RBM15B/STK36/EIF5/CNOT3/TBP/PACSIN1/EIF4EBP2/CHIC2/NUDCD2/ATG5/ATF6B/SMARCD1/PGRMC2/DNAJC7/CTDSP1/MLST8/ATG9A/RELA/NUDT5/HDAC10/MTA1/RPS6KC1/CORO7/ARID1A/PA2G4/EP300/COPG1/THBD/FLAD1/XRN2/FBXO11/HMGB1/EID1/CAMTA2/ARFGAP3/ADAMTSL4/SETD1B/PPIL2/HNRNPM/AKT1S1/NIPBL/SMARCB1/RPL9/TTYH2/GMPPA/TMA16/HNRNPD/ARHGDIA/SMG9/TAF4/TAF3/TAF6/NIN/CCPG1/MICU2/MEA1/SMG7/SPPL2B/SPPL2A/HNRNPH3/OTUB1/DYRK1B/SMARCC2/TSC2/ATF7/HDAC7/HDAC6/SEL1L/NDUFAF5/RAI1/E2F4/FOSL2/LYAR/ARID4B/GABBR1/BAP1/RGP1/TMF1/KIF2C/ALAS1/SUCLA2/LBR/PTGER4/LIMK1/ATP6V1H/STXBP3/MID1IP1/HMGA1/ATP6V1D/TMEM242/SH2D3C/NCOA5/CPSF7/MAPRE1/CARM1/KDM6B/EPN1/WIZ/DPF2/KMT2D/GPSM3/KMT2B/PPM1B/SEC63/CCDC120/SELPLG/VPS36/NFATC1/ALKBH6/RSG1/PDCD11/TMC8/FLT4/CBL/ANXA1/SAMHD1/SMC2/ANXA4/CDC25B/SMC4/CLPTM1/RNF115/FAF1/JAK3/GGA1/TBL1X/GGA3/HM13/PRR14/PRR13/VAPB/SRP68/PTPN23/RAB1B/RAB1A/HOOK3/AGPS/COL4A3BP/KIFAP3/RARA/PELI2/FAM103A1/CFI/CLNS1A/RPP25/PLD2/RARG/CD3E/UBR2/AP4M1/TMEM150A/HES1/CD37/FAM86C1/NOP2/RFC1/FBXL8/PIAS3/SERBP1/TBRG1/NEK8/LRCH4/BLCAP/CD74/UHMK1/HNRNPA3/VRK1/R3HCC1L/KRAS/ACD/GYS1/POLM/SLC4A1AP/OLFM2/SRP54/UFSP2/HPCAL1/MAML1/FAM111A/CREBBP/DDX1/EPRS/SMAD3/SKI/HNRNPA1/ATP13A2/PTPN11/RNF7/RIT1/ENG/PPARD/RAB5B/KIAA0368/ATP5B/WASF2/ANKRD10/SHOC2/MLH3/NFKB2/TERF2IP/KCNIP2/CANX/ARL2BP/WBP2/BAG6/BAG3/TRAPPC6B/GOLGA4/TOMM34/ZFP36/CCNF/DNAJC21/LENG8/ZDHHC17/SIAH1/RBM39/MAP3K14/SRCAP/MAP3K12/TOP3B/MAP3K11/RIN3/USP7/MTDH/WASH1/KLC2/CIC/ZFP36L1/SUMO2/SUMO1/ATXN2L/AGO1/FASN/GATAD2A/ABCD3/MLLT1/AATF/MLLT6/BAZ2A/RAB2A/BCAS2/GDI1/IPO13/DLGAP4/C12ORF43/ANKRD40/PSMD14/MAPK14/ARSA/APBB3/UBXN4/TOB2/FAM126B/RBM17/GOLGB1/PLXNA3/WFS1/FAM189B/CBX4/GUCD1/CBX6/TAPBP/TBC1D15/GABPB1/ANKRD54/TBC1D17/SCLY/SPRED2/RBCK1/IFRD2/SCAMP2/PLXNB1/USP1/TNFRSF14/FGF21/IGSF8/USO1/C1ORF189/RIPK3/SLU7/VPS26A/SNRPE/GPN1/SYVN1/SRM/PNPT1/MAP4K2/ELK1/SNX4/COPS7B/ELK3/TRRAP/SRF/FTH1/RRAGC/IRAK4/UBE2D3/PSMB1/NDRG1/SSX2IP/RUNX1/THAP11/SREBF1/HAVCR2/YIPF6/PTPN2/CKAP5/ABHD14B/TSN/SUGT1/SREBF2/ATE1/AK8/PSMC6/PHF2/PHF1/TMEM8A/ULK1/SLAIN2/PSMC1/YIPF3/RAP1A/RAP1B/GPATCH4/SCMH1/CDK18/HPS3/CLTC/LARP1/MEN1/PIGK/SLK/CCDC43/CDIP1/PAK4/ARHGAP1/ZYX/CDK16/CSK/PSMD7/SP100/FOXJ2/LDB1/NOL8/MINK1/CCDC47/PFKM/NLRP1/EIF4G2/PRKD2/DDR1/ZMIZ2/CCDC53/COMMD1/KPNA3/COMMD6/SPAST/RUSC1-AS1/UTP3/GLTSCR1/TNF/XIAP/LMF2/TRA2B/HCFC1/PPP3R2/PSMF1/CENPA/POU2F1/IL2RG/RSL24D1/POM121C/PIK3R2/SYNPO/NDUFA4/KAT2A/ZBTB7A/DTX1/ATXN7L1/FAM188A/SIRT6/RGS14/SH3BP5/MAP1S/THRAP3/IRF2/TMEM86B/SH3BP1/ARAP3/CDK20 |
| MF | GO:0044822/poly(A) RNA binding | 100 | 9.89E-12 | CHERP/RBM15B/EIF5/SRP68/NSRP1/CLK3/DHX34/FAM103A1/LSM1/SAMD4B/RPP25/CLNS1A/ROCK2/PTBP1/PA2G4/NOP2/SERBP1/LLPH/USO1/DNTTIP2/XRN2/HMGB1/PNPT1/PRRC2A/MANF/HNRNPA3/HNRNPM/DDX46/SPATS2/HNRNPD/ISY1/MEPCE/SRP54/DDX1/HNRNPA1/SRSF3/HNRNPH3/R3HDM2/PSMC1/PPRC1/GPATCH4/ZNF385A/FBRSL1/LYAR/CLTC/CANX/LARP1/FAM133B/ANP32A/ZYX/LBR/ZFP36/EXOSC9/NOL8/CCDC47/DNAJC21/EIF4G2/TRNAU1AP/FAM120A/TAF15/NCOA5/EIF4A2/TXN/CPSF7/NOL10/RBM39/MAPRE1/TOP3B/KIAA1429/UTP3/RBM34/MTDH/TRA2B/BCCIP/SF3B4/SEC63/ZFP36L1/SRRT/SUMO2/SUMO1/ATXN2L/CSDE1/AGO1/FASN/AATF/USP36/UNK/RTCA/PDCD11/CS/ELAVL1/PPP1R10/SF3A2/SF3A1/RPL23/HNRNPUL2/HNRNPUL1/THRAP3/SYF2/GOLGB1 |
| MF | GO:0003682/chromatin binding | 42 | 1.64E-07 | HMGN1/CRTC2/CAMTA2/FOSL2/CBX4/BAP1/HCFC1/ELK1/NFKB2/MLH3/CIC/CITED2/MEN1/NIPBL/CENPA/SMARCD1/HNRNPD/BRD4/KAT2A/SREBF1/BRD2/LDB1/RELA/CREBBP/TP53/MBD6/MED12/DDX1/MTA1/SKI/SIRT6/PHF12/HMGA1/EP300/PHF1/NCOA5/SMARCC2/MNT/KDM6B/RERE/HDAC7/ATAD2B |
| MF | GO:0008134/transcription factor binding | 34 | 2.58E-07 | USP7/HMGB1/CAMTA2/PPARD/E2F4/STK36/MED25/FIGLA/TBP/DAXX/SRF/SORBS3/SUMO1/HNRNPD/RARA/RUNX1/ELMSAN1/KAT2A/TAF4/PDCD11/SP100/RELA/CREBBP/TP53/SMAD3/HMGA1/DOT1L/HES1/EP300/NAB2/ATF7/PPRC1/APBB3/TBL1X |
| MF | GO:0003713/transcription coactivator activity | 29 | 3.67E-06 | PPARD/MTDH/HCFC1/NFKB2/ARL2BP/CITED2/SMARCB1/SMARCD1/RARA/SERTAD2/KAT2A/TAF4/SP100/DTX1/MAML1/CREBBP/MED12/MTA1/ARID1A/EP300/PHF2/DYRK1B/THRAP3/SMARCC2/RIPK3/USP21/MNT/CARM1/SRCAP |
| MF | GO:0004672/protein kinase activity | 34 | 4.12E-05 | CDK18/STK36/MKNK2/MAP4K2/RPS6KB2/IRAK4/SGK223/VRK1/CLK3/COL4A3BP/PAK4/CDK16/SIK3/PDK2/MAP2K1/ROCK1/LIMK1/MAP2K2/RPS6KC1/MINK1/DDR1/PRKD2/C19ORF35/SCYL1/SCYL2/MAPK14/DYRK1B/ARAF/RIPK3/NEK8/MAP3K14/CDK20/MAP3K12/MAP3K11 |
| MF | GO:0004674/protein serine/threonine kinase activity | 33 | 2.25E-04 | CDK18/STK36/MKNK2/MAP4K2/RPS6KB2/UHMK1/IRAK4/VRK1/SLK/CLK3/PAK4/CDK16/SIK3/MAP2K1/ROCK1/LIMK1/ROCK2/MAP2K2/RPS6KC1/MINK1/MAST3/PRKD2/ULK1/GSK3A/MAPK14/DYRK1B/ARAF/RIPK3/NEK8/MAP3K14/CDK20/MAP3K12/MAP3K11 |
| MF | GO:0098641/cadherin binding involved in cell-cell adhesion | 27 | 3.86E-04 | VAPB/EIF5/WASF2/HCFC1/ABI1/KLC2/RAB1A/LARP1/ATXN2L/SLK/BAG3/PAK4/ARHGAP1/FASN/RAB11B/NDRG1/STX5/CKAP5/CBL/ANXA1/LYPLA2/EIF4G2/SCYL1/SERBP1/USO1/MAPRE1/PARVA |
| MF | GO:0004843/thiol-dependent ubiquitin-specific protease activity | 12 | 6.47E-04 | USP7/USP19/OTUB1/USP1/USP21/BAP1/USP36/USP25/USP42/FAM63B/USP33/VCPIP1 |
| MF | GO:0051059/NF-kappaB binding | 7 | 1.58E-03 | PPARD/MTDH/PIAS3/RELA/FAF1/COMMD6/ANXA4 |
| MF | GO:0003700/transcription factor activity, sequence-specific DNA binding | 62 | 1.76E-03 | POU6F1/AEBP1/PPARD/RAI1/FOSL2/E2F4/ARID4A/SPI1/TBP/NFKB2/CITED2/GABPB1/HSF1/ATF6B/CREB3L2/SPIB/RARA/ELMSAN1/RARG/RELA/TP53/MTA1/HMGA1/HES1/PA2G4/MNT/HMGB1/ZNF552/KMT2B/ELK1/HCFC1/ELK3/SRF/ZFP36L1/NPAS1/TSC22D2/ZSCAN22/POU2F2/GATAD2A/POU2F1/MLXIP/AATF/RUNX1/PLAGL2/NFATC1/ZNF564/SREBF1/IKZF4/TAF4/TAF6/CREBBP/CBL/SMAD3/SREBF2/MEF2D/PHF1/ATF7/IRF2/SCMH1/NFIC/RERE/GOLGB1 |
| MF | GO:0044212/transcription regulatory region DNA binding | 20 | 2.38E-03 | ZNF564/IKZF4/KMT2D/TNF/ARID4A/RELA/ARID4B/TP53/CBX4/SMAD3/TBP/ZNF335/SREBF2/MEN1/GABPB1/ATF6B/CREB3L2/TBL1X/CARM1/ELMSAN1 |
| MF | GO:0003714/transcription corepressor activity | 19 | 3.26E-03 | EID1/AEBP1/SP100/LDB1/CBX4/MTA1/SIRT6/SKI/ELK3/DAXX/CITED2/ATN1/NAB2/MNT/RARA/RUNX1/TBL1X/TOB2/HDAC7 |
| MF | GO:0000978/RNA polymerase II core promoter proximal region sequence-specific DNA binding | 28 | 3.45E-03 | DPF2/E2F4/FOSL2/ELF4/SPI1/FIGLA/ELK1/TBP/NFKB2/ELK3/SRF/HSF1/SMARCB1/POU2F2/CREB3L2/AGO1/SREBF1/ZBTB7A/ZNF100/FOXJ2/RELA/SMAD3/SKI/SREBF2/MEF2D/SMARCC2/ATF7/NFIC |
| MF | GO:0000166/nucleotide binding | 27 | 5.05E-03 | HINT3/RBM34/RBM15B/SETD1B/TRA2B/SF3B4/HNRNPA3/SRRT/HNRNPM/R3HCC1L/HNRNPD/DXO/NOL8/PTBP1/ELAVL1/VDAC3/ATP13A2/HNRNPA1/SRSF3/TRNAU1AP/HNRNPH3/TAF15/CPSF7/PPRC1/RBM39/PABPC1L/RBM17 |
| MF | GO:0005096/GTPase activator activity | 23 | 5.14E-03 | GIT1/GDI1/A2M/ARFGAP3/TBC1D3F/PLXNB1/SIPA1L3/RABGAP1L/RGS14/TBC1D3B/TBC1D15/CDC42EP2/TBC1D17/BNIP2/ACAP2/TSC2/ARHGAP1/ARAP3/SH3BP1/RASA4/ARHGDIA/PIK3R2/RIN3 |
| MF | GO:0019903/protein phosphatase binding | 9 | 5.66E-03 | KAT2A/MAPK14/FLT4/TP53/SHOC2/JAK3/CSK/AP3B1/PIK3R2 |
| MF | GO:0070577/lysine-acetylated histone binding | 5 | 6.60E-03 | BRD2/BRD4/CARM1/BAZ2A/ATAD2B |
| MF | GO:0031625/ubiquitin protein ligase binding | 23 | 7.17E-03 | USP7/HM13/WASH1/WFS1/DTX1/RELA/TP53/SMAD3/TNFRSF14/SKI/TMEM189/DAXX/NAE1/SUMO2/USP19/SUMO1/PA2G4/BAG6/OTUB1/SPG20/FAF1/USP25/HDAC6 |
| MF | GO:0003729/mRNA binding | 13 | 8.11E-03 | ZFP36/ZFP36L1/HNRNPA3/TRA2B/ELAVL1/CLUH/SLC4A1AP/TSN/NSRP1/LSM1/DXO/UHMK1/SAMD4B |
| MF | GO:0002039/p53 binding | 9 | 8.20E-03 | USP7/TAF3/EP300/SMARCB1/CREBBP/TP53/BRD4/DAXX/ZNF385A |
| MF | GO:0019003/GDP binding | 8 | 8.42E-03 | RAB2A/SRP54/KRAS/RAB5B/RAB11B/RAP1B/RRAGC/RAB27A |
| MF | GO:0031072/heat shock protein binding | 7 | 9.02E-03 | ZFP36/HSF1/LIMK1/DNAJC7/FAF1/DAXX/TOMM34 |
| MF | GO:0047485/protein N-terminus binding | 11 | 9.30E-03 | MEN1/STX5/MNAT1/NIPBL/MAP2K1/PIAS3/RELA/TP53/EXOC4/KCNIP2/DAXX |
| MF | GO:0003727/single-stranded RNA binding | 7 | 1.01E-02 | ZFP36/HMGB1/ANXA1/AGO1/CBX4/HNRNPA1/CBX6 |
| MF | GO:0035925/mRNA 3'-UTR AU-rich region binding | 4 | 1.03E-02 | ZFP36/ZFP36L1/HNRNPD/ELAVL1 |
| MF | GO:0036459/thiol-dependent ubiquitinyl hydrolase activity | 9 | 1.06E-02 | USP7/USP19/USP1/USP21/FAM188A/USP36/USP25/USP42/USP33 |
| MF | GO:0008565/protein transporter activity | 9 | 1.25E-02 | PGAP2/ARFGAP3/TMCO6/IPO13/USO1/RAP1A/KPNA3/SEC62/VPS26A |
| MF | GO:0008022/protein C-terminus binding | 16 | 1.30E-02 | USP7/MAP2K1/MED12/MID1IP1/PFKM/SREBF2/EP300/NIPBL/PIAS3/PPP2CB/SIAH1/MAPRE1/CSK/TBL1X/KPNA3/VPS36 |
| MF | GO:0042826/histone deacetylase binding | 11 | 1.30E-02 | KAT2A/HES1/MEF2D/CAMTA2/NIPBL/RELA/HNRNPD/HDAC10/RARA/SRF/HDAC6 |
| MF | GO:0019901/protein kinase binding | 27 | 1.33E-02 | PTPN23/CLTC/DAXX/VRK1/HSF1/TICAM1/GYS1/SPRED2/PKD1/SREBF1/ZFP36/MAP2K1/PTPN2/CD3E/RELA/MAML1/TP53/SMAD3/ELAVL1/SKI/WAS/RGS14/CDC25B/ZBTB4/FAF1/HDAC7/MAP3K12 |
| MF | GO:0033613/activating transcription factor binding | 5 | 1.37E-02 | MEF2D/EP300/WFS1/RELA/HDAC7 |
| MF | GO:0008270/zinc ion binding | 67 | 1.63E-02 | AEBP1/PPARD/RAI1/AGTPBP1/MAN2B2/BRPF3/RBCK1/RARA/ZYX/SP100/RARG/LIMK1/TP53/MTA1/MMP17/VPS41/UBR2/DNAJC21/MAN2A2/ZDHHC17/EP300/TAF15/PIAS3/ZMIZ2/ZMIZ1/SLU7/SIAH1/RNF26/FBXO11/DPF2/KMT2D/SYVN1/XIAP/KMT2B/DUSP12/ZDHHC18/RABGGTA/ACE/GATAD2A/LIMD2/TCEA1/MLLT6/THAP11/BAZ2A/USP33/TAF3/ZMYM3/DTX1/CREBBP/CBL/SMAD3/SAMHD1/SIRT6/SKI/PHF12/SF3A2/ATP13A2/RLF/MNAT1/RNF44/PHF2/RNF115/RNF7/PHF1/ZNF385A/RERE/HDAC6 |
| MF | GO:0042500/aspartic endopeptidase activity, intramembrane cleaving | 3 | 1.72E-02 | HM13/SPPL2B/SPPL2A |
| MF | GO:0032403/protein complex binding | 17 | 1.77E-02 | NDUFA4/SREBF1/GIT1/RELA/WASF2/ABI1/TSN/ANKRD54/MTHFR/PPP1CA/KRAS/ULK1/BAG3/RIPK3/RAP1A/RAP1B/TCEB1 |
| MF | GO:0042162/telomeric DNA binding | 5 | 1.87E-02 | UPF2/ACD/SMG7/HNRNPD/TERF2IP |
| MF | GO:0019904/protein domain specific binding | 17 | 1.92E-02 | XPO1/SP100/E2F4/MED12/SKI/NLRP1/PTPN11/HNRNPM/ATN1/RFC1/POU2F2/PKD1/RARA/FAF1/SSX2IP/TBL1X/RAB27A |
| MF | GO:0001205/transcriptional activator activity, RNA polymerase II distal enhancer sequence-specific binding | 5 | 2.15E-02 | RELA/SPI1/HCFC1/HMGA1/NFATC1 |
| MF | GO:0030374/ligand-dependent nuclear receptor transcription coactivator activity | 7 | 2.23E-02 | ZMIZ2/THRAP3/PPRC1/MED12/MED24/CARM1/HMGA1 |
| MF | GO:0042809/vitamin D receptor binding | 4 | 2.50E-02 | THRAP3/MED12/MED24/TOB2 |
| MF | GO:0070087/chromo shadow domain binding | 3 | 2.51E-02 | NIPBL/SP100/LBR |
| MF | GO:0004430/1-phosphatidylinositol 4-kinase activity | 3 | 2.51E-02 | PI4KAP2/PI4KAP1/PI4KB |
| MF | GO:0004702/receptor signaling protein serine/threonine kinase activity | 7 | 2.65E-02 | SLK/MAP2K1/MAP2K2/PAK4/MAP4K2/MINK1/MAP3K14 |
| MF | GO:0019789/SUMO transferase activity | 4 | 2.98E-02 | SUMO2/PIAS3/CBX4/HDAC7 |
| MF | GO:0017137/Rab GTPase binding | 12 | 3.14E-02 | GDI1/TBC1D15/TMEM127/ULK1/ACAP2/VPS52/RABGAP1L/NDRG1/RGP1/TBC1D3B/RIN3/RABGGTA |
| MF | GO:0003712/transcription cofactor activity | 8 | 3.38E-02 | EP300/THRAP3/CREBBP/DDX1/MED12/MED24/TRRAP/TMF1 |
| MF | GO:0017124/SH3 domain binding | 11 | 3.40E-02 | SH3BP5/OSTF1/DTX1/CD3E/WASF2/PLSCR3/ARHGAP1/CBL/ABI1/SH3BP1/WAS |
| MF | GO:0019237/centromeric DNA binding | 3 | 3.41E-02 | KIF2C/CENPA/MLH3 |
| MF | GO:0030695/GTPase regulator activity | 4 | 3.51E-02 | GPSM3/PCP2/WAS/ARL2BP |
| MF | GO:0017091/AU-rich element binding | 4 | 3.51E-02 | ZFP36/ZFP36L1/EXOSC9/ELAVL1 |
| MF | GO:0000979/RNA polymerase II core promoter sequence-specific DNA binding | 7 | 3.63E-02 | EP300/HSF1/SPI1/ZNF277/TBP/ZNF335/WBP2 |
| MF | GO:0031490/chromatin DNA binding | 7 | 3.90E-02 | EP300/RELA/SMAD3/RARA/SRF/KDM4D/WBP2 |
| MF | GO:0016757/transferase activity, transferring glycosyl groups | 7 | 4.81E-02 | B4GALT1/MFNG/GALNT1/B4GALT3/GALNT7/PIGB/DPM2 |
| MF | GO:0003924/GTPase activity | 17 | 4.89E-02 | GPN1/RAB2A/SRP54/OPA1/RAB5B/EIF5/ARF5/RAB1A/RRAGC/KRAS/MTG1/RAB11B/RAP1A/RIT1/RAP1B/NKIRAS2/RAB27A |
| MF | GO:0005524/ATP binding | 79 | 4.95E-02 | KIFC2/DYNC1LI2/CDK18/STK36/ATP5B/RPS6KB2/MLH3/TTLL3/KIF2C/CLK3/SLK/PAK4/DHX34/CSK/CDK16/SUCLA2/SIK3/ROCK1/LIMK1/ROCK2/TP53/MINK1/RPS6KC1/PFKM/PI4KB/NLRP1/MAST3/PRKD2/DDR1/RFC1/SCYL1/SCYL2/EIF4A2/RIPK3/NEK8/YME1L1/MAP3K14/FLAD1/SRCAP/SPAST/MAP3K12/MAP3K11/PGS1/MKNK2/MAP4K2/UHMK1/PFAS/IRAK4/SGK223/VRK1/UBE2D3/KRAS/DDX46/ABCD3/GK5/RUNX1/RTCA/PDK2/MAP2K1/MAP2K2/FLT4/DDX1/EPRS/ATP13A3/ATP13A2/SMC2/SMC4/AK8/PSMC6/GSK3A/ULK1/DYRK1B/MAPK14/ARAF/PSMC1/THRAP3/JAK3/CDK20/ATAD2B |

Supplementary Table 2 GO enrichment and DEGs at discharge

| Category | Term | Count | PValue | Genes |
| --- | --- | --- | --- | --- |
| BP | GO:0006468/protein phosphorylation | 41 | 3.23E-05 | PRKCZ/FASTKD1/ZAK/PDGFB/PHKB/PASK/MAP4K2/MAP4K1/FER/RIOK1/ST3GAL1/IRAK3/SBK1/COL4A3BP/LMTK3/CDK16/CHUK/TEC/CSNK1A1/ADAM10/SGK3/ROCK1/ROCK2/PIK3CD/RPS6KC1/MINK1/RAF1/CCNC/SMAD2/STK3/CDC25B/IKBKE/SCYL2/DYRK1B/ERN1/GRK6/BMP2K/MAPK9/LRRK2/PRKD3/NRBP2 |
| BP | GO:0007264/small GTPase mediated signal transduction | 26 | 1.01E-04 | RAB2A/GDI1/PLD1/VAV3/ARHGEF18/TRIM23/RAB1A/RALGDS/RABL2B/SH2D3C/SH2D3A/RAB18/MFHAS1/RASGRP2/SOS2/ARHGAP1/RHOT1/RHEB/RIT1/ARL8B/LRRK2/AGAP2/RHOF/NKIRAS1/RAB21/RAB27A |
| BP | GO:0006886/intracellular protein transport | 25 | 1.37E-04 | VPS29/STX7/RPGR/XPO6/AP1G2/AP4E1/TBC1D10A/VPS45/STAM2/VPS41/IPO8/NAPB/CD74/EVI5/ANKRD50/IPO4/USO1/SLU7/APPBP2/EHD1/TNPO2/SNX13/SYTL1/VPS26A/AP3B1 |
| BP | GO:0007249/I-kappaB kinase/NF-kappaB signaling | 11 | 2.80E-04 | TRAF2/TNF/NLRC3/ROCK1/ROCK2/TBK1/MAP3K14/TIAF1/NKIRAS1/AZI2/CHUK |
| BP | GO:0006461/protein complex assembly | 15 | 6.20E-04 | TRAF1/TRAF2/KMT2A/CD3E/CAPZA2/TP53/SLC9A3R1/SKAP2/HMGA1/LLGL1/CD74/TAPBP/MDM2/ZW10/APC |
| BP | GO:0016925/protein sumoylation | 14 | 2.05E-03 | XRCC4/TP53/MTA1/SMC6/NUP188/RANGAP1/BRCA1/SENP2/NUP210/MDM2/NSMCE2/NUP54/SCMH1/HDAC7 |
| BP | GO:0034613/cellular protein localization | 8 | 2.63E-03 | SIN3A/NIPBL/TP53/ARMCX3/ZDHHC18/AXIN2/LRRK2/RRAGB |
| BP | GO:0000389/mRNA 3'-splice site recognition | 4 | 2.66E-03 | SF1/ISY1/SLU7/SF3A2 |
| BP | GO:0043161/proteasome-mediated ubiquitin-dependent protein catabolic process | 19 | 4.13E-03 | CSNK1A1/TP53/MTA1/RNF187/RFFL/EDEM3/ATXN3/RNF44/RMND5A/PSMA6/UBXN2B/BTBD1/PPP2CB/PSMD1/SIAH1/SPOPL/PSMD6/UBE2D1/APC |
| BP | GO:0042110/T cell activation | 8 | 4.40E-03 | LAT/NLRC3/CD3E/PIK3CD/SMAD3/FOXP3/AZI2/IFNAR1 |
| BP | GO:0045893/positive regulation of transcription, DNA-templated | 37 | 4.83E-03 | ENY2/PPARD/TNF/FOXK1/PDGFB/KMT2A/CRLF3/PPM1A/FOXO1/RORC/ELK1/MED24/RNF187/TFAM/WNT1/SFR1/NR1D1/GATA3/ASPH/RUNX1/DNAJC2/NFATC1/PCBD2/RELA/TP53/MED12/SMAD3/SMAD2/FOXP3/HMGA1/BRCA1/IFNAR1/BTBD8/DYRK1B/SMARCC2/KLF2/PBX4 |
| BP | GO:0007030/Golgi organization | 10 | 5.32E-03 | CSNK1A1/RAB2A/ATL2/UBXN2B/USO1/VMP1/LRRK2/RAB1A/ZW10/ATP8B4 |
| BP | GO:0009967/positive regulation of signal transduction | 9 | 5.32E-03 | LAT/SH2D3C/VAV3/SH2D3A/GATA3/ARHGAP1/CNTNAP1/SKAP2/PAG1 |
| BP | GO:0015031/protein transport | 30 | 5.59E-03 | VPS29/ENY2/SLC15A2/SNX14/VPS54/KIAA0196/UNC50/PTPN23/SNX4/HOOK3/SFT2D1/DNAJC13/NUP210/KIAA1033/EXOC6/AGAP2/RAB21/ZW10/RAB2A/GDI1/VTA1/CORO7/VPS8/SENP2/SCFD1/PLEKHF2/CHMP1B/RAB18/ATG4C/IPO4 |
| BP | GO:0032715/negative regulation of interleukin-6 production | 6 | 7.13E-03 | IRAK3/TNF/NLRC3/HGF/KLF2/FOXP3 |
| BP | GO:0042787/protein ubiquitination involved in ubiquitin-dependent protein catabolic process | 15 | 8.20E-03 | FZR1/RNF217/RFFL/KLHL8/RMND5A/HUWE1/BTBD1/MDM2/SIAH1/ITCH/RCHY1/SPOPL/UBE2D1/RNF146/TRIP12 |
| BP | GO:0016055/Wnt signaling pathway | 17 | 9.21E-03 | CSNK1A1/VPS29/WNT10A/LZTS2/TCF7/LDB1/PPM1A/SLC9A3R1/SENP2/WNT1/AES/GRK6/NDRG2/AXIN2/VPS26A/RNF146/APC |
| BP | GO:0032480/negative regulation of type I interferon production | 6 | 9.61E-03 | NLRC5/IKBKE/TBK1/IRF3/ITCH/TAX1BP1 |
| BP | GO:0043984/histone H4-K16 acetylation | 5 | 1.06E-02 | JADE2/KMT2A/KANSL3/HCFC1/KANSL1L |
| BP | GO:1903347/negative regulation of bicellular tight junction assembly | 3 | 1.11E-02 | TNF/ROCK1/ROCK2 |
| BP | GO:0043547/positive regulation of GTPase activity | 38 | 1.17E-02 | ALS2/PDGFB/AGFG2/ARHGAP19/RANGAP1/RGP1/LLGL1/RASGRP2/SOS2/ARHGAP1/IL2RG/SHC1/AXIN2/AGAP2/RASA1/ELMOD2/ARHGDIA/FGD4/GIT1/GDI1/VAV3/ARHGEF1/RPGR/MCF2/ARHGEF18/ARHGAP29/DENND1C/RGS14/RALGDS/FNIP1/LAT/SH2D3C/SH2D3A/SBF1/BNIP2/TBCD/LRRK2/SNX13 |
| BP | GO:0007265/Ras protein signal transduction | 9 | 1.21E-02 | LAT/PLD1/MAPK14/RASGRP2/TP53/SDCBP/SHC1/RIT1/RALGDS |
| BP | GO:0046677/response to antibiotic | 6 | 1.26E-02 | CYB5R4/CCR4/PPP2CB/TP53/MDM2/SKIL |
| BP | GO:0032091/negative regulation of protein binding | 8 | 1.26E-02 | SENP2/ACE/AES/TMC8/ROCK1/PDGFB/LRRK2/USP33 |
| BP | GO:1902042/negative regulation of extrinsic apoptotic signaling pathway via death domain receptors | 6 | 1.44E-02 | TRAF2/CFLAR/RAF1/RFFL/HGF/BRCA1 |
| BP | GO:0050852/T cell receptor signaling pathway | 14 | 1.46E-02 | PIK3CB/CD3E/RELA/PIK3CD/FOXP3/LAT/PSMA6/GATA3/PSMD1/LIME1/PSMD6/UBE2D1/CHUK/PAG1 |
| BP | GO:0070207/protein homotrimerization | 5 | 1.49E-02 | TRAF2/SKI/SKIL/SIGMAR1/MGST1 |
| BP | GO:0006914/autophagy | 13 | 1.52E-02 | HMGB1/PIK3CB/STAM2/VTA1/FOXO1/VPS41/RAB1A/ATG4C/RB1CC1/ULK3/VMP1/LRRK2/DRAM1 |
| BP | GO:0070536/protein K63-linked deubiquitination | 5 | 1.74E-02 | ATXN3/USP8/FAM175B/USP20/USP33 |
| BP | GO:0035509/negative regulation of myosin-light-chain-phosphatase activity | 3 | 1.80E-02 | TNF/ROCK1/ROCK2 |
| BP | GO:0071108/protein K48-linked deubiquitination | 5 | 2.01E-02 | ATXN3/USP8/FAM188A/USP20/USP33 |
| BP | GO:0007254/JNK cascade | 7 | 2.06E-02 | SH2D3C/TNF/SH2D3A/MAP2K4/MAP4K2/MINK1/MAPK9 |
| BP | GO:0035556/intracellular signal transduction | 28 | 2.20E-02 | PRKCZ/ZAK/MAP4K2/MAP4K1/FER/PLCH2/SHC1/PLCD1/RASA1/PAG1/TEC/NFATC1/SGK3/MCF2/RAF1/MINK1/SMAD2/ARHGAP29/STK3/RGS14/LAT/MAPK14/PRKAR1A/DGKZ/SDCBP/LRRK2/PRKD3/NRBP2 |
| BP | GO:0051865/protein autoubiquitination | 7 | 2.26E-02 | TRAF2/RNF141/MDM2/RNF187/RCHY1/BRCA1/RNF146 |
| BP | GO:0007266/Rho protein signal transduction | 7 | 2.26E-02 | ARHGEF1/ROCK1/ROCK2/ARHGAP1/ARHGAP29/ARHGDIA/CHUK |
| BP | GO:0060612/adipose tissue development | 5 | 2.31E-02 | PPARD/DYRK1B/SPG20/RORC/ACAT1 |
| BP | GO:0014842/regulation of skeletal muscle satellite cell proliferation | 3 | 2.62E-02 | CFLAR/PPARD/PAXBP1 |
| BP | GO:0035023/regulation of Rho protein signal transduction | 9 | 2.70E-02 | ALS2/VAV3/ARHGEF1/MCF2/ARHGEF18/SOS2/RAF1/ARHGDIA/FGD4 |
| BP | GO:0038095/Fc-epsilon receptor signaling pathway | 15 | 2.74E-02 | VAV3/PIK3CB/RELA/MAP2K4/FER/LAT/PSMA6/PSMD1/MAPK9/SHC1/PSMD6/UBE2D1/CHUK/TEC/NFATC1 |
| BP | GO:0006915/apoptotic process | 36 | 3.05E-02 | ZFAND6/TRAF1/PACS2/PPARD/KMT2A/ADORA2A/TNFRSF25/GPR65/FOXO1/CXCR3/RFFL/PTRH2/PPP2CA/ITCH/DDIAS/CFLAR/AIMP1/MAP2K4/TP53/RAF1/FAM188A/ELMO3/BRCA1/STK3/TAX1BP1/FXR1/RNF130/TRIM35/BNIP2/MAPK14/PLSCR3/IRF3/SIAH1/APAF1/TIAF1/DRAM1 |
| BP | GO:0043982/histone H4-K8 acetylation | 4 | 3.16E-02 | JADE2/KANSL3/HCFC1/KANSL1L |
| BP | GO:0043981/histone H4-K5 acetylation | 4 | 3.16E-02 | JADE2/KANSL3/HCFC1/KANSL1L |
| BP | GO:0006511/ubiquitin-dependent protein catabolic process | 15 | 3.23E-02 | USP8/UBR3/RFFL/ATXN3/PSMA6/CUL9/UCHL5/USP20/USP36/SIAH1/ITCH/UBE2D1/USP15/USP33/FBXO11 |
| BP | GO:0035666/TRIF-dependent toll-like receptor signaling pathway | 5 | 3.37E-02 | IKBKE/TBK1/IRF3/UBE2D1/CHUK |
| BP | GO:1903140/regulation of establishment of endothelial barrier | 3 | 3.56E-02 | TNF/ROCK1/ROCK2 |
| BP | GO:0022900/electron transport chain | 3 | 3.56E-02 | NDUFS4/ETFDH/CYB561 |
| BP | GO:0030252/growth hormone secretion | 3 | 3.56E-02 | LTBP4/CDK16/RAB1A |
| BP | GO:0051056/regulation of small GTPase mediated signal transduction | 12 | 3.62E-02 | GDI1/VAV3/MCF2/ARHGEF18/ARHGAP1/SOS2/ARHGAP19/RHOT1/ARHGAP29/RHOF/ARHGDIA/FGD4 |
| BP | GO:0030033/microvillus assembly | 4 | 3.72E-02 | MINK1/RDX/FXYD5/SLC9A3R1 |
| BP | GO:0090002/establishment of protein localization to plasma membrane | 6 | 3.72E-02 | TNF/ROCK1/ROCK2/RDX/SLC9A3R1/FAM126A |
| BP | GO:0000226/microtubule cytoskeleton organization | 8 | 3.76E-02 | PRKCZ/ATXN3/CUL9/ATXN7/TBCE/FER/MARK3/PPP2R3C |
| BP | GO:0048873/homeostasis of number of cells within a tissue | 5 | 3.78E-02 | KMT2A/VPS54/CSF1/FH/FLT3LG |
| BP | GO:0010803/regulation of tumor necrosis factor-mediated signaling pathway | 5 | 4.22E-02 | TRAF1/TRAF2/TNF/TAX1BP1/CHUK |
| BP | GO:0010628/positive regulation of gene expression | 19 | 4.40E-02 | WNT10A/PPARD/TNF/PDGFB/ROCK2/PIK3CB/CD3E/CSF1/PIK3CD/TP53/SMAD3/HCFC1/ITGA3/RDX/BRCA1/MAPK14/MAPK9/MDM2/RAB27A |
| BP | GO:0071550/death-inducing signaling complex assembly | 3 | 4.61E-02 | TRAF2/TNF/RAF1 |
| BP | GO:0043068/positive regulation of programmed cell death | 3 | 4.61E-02 | TNF/RARG/LRRK2 |
| BP | GO:0042117/monocyte activation | 3 | 4.61E-02 | ADAM10/CSF1/ADAM9 |
| BP | GO:0050863/regulation of T cell activation | 3 | 4.61E-02 | LAT/SIT1/PAG1 |
| BP | GO:2000643/positive regulation of early endosome to late endosome transport | 3 | 4.61E-02 | PTPN23/RDX/RAB21 |
| BP | GO:2000210/positive regulation of anoikis | 3 | 4.61E-02 | AES/PTRH2/MYBBP1A |
| CC | GO:0005829/cytosol | 206 | 1.44E-08 | ALS2/SCPEP1/XRCC4/VPS54/PPP2R5D/RNF217/RANGAP1/RAB1A/HOOK3/COL4A3BP/ROCK1/ROCK2/PIK3CB/PIK3CD/UBR2/VPS41/MARK3/CEP162/RAB18/HUWE1/FBXL8/MAPK9/HSD17B11/ADSS/RFFL/HNMT/IDH1/FH/GIT1/SRP54/MAP2K4/NPL/SMAD3/FAM213B/SMAD2/TAX1BP1/DENND1C/PTPN12/GBE1/RHEB/RANBP10/PHKB/COPS4/EVI5/PBXIP1/EEF2K/ARHGEF1/ARHGEF18/SNUPN/ZDHHC8/PIK3C2B/CNOT10/TP53/ATP6V1C1/RAB11FIP3/CHMP1B/BNIP2/SIAH1/MAP3K14/SDCCAG8/USP8/KIAA0196/PPP6R1/EEA1/KLC2/FBF1/SORBS3/RB1CC1/PPP2CA/SOS2/PPP2CB/ABCD3/EXOC6/LYPLAL1/MTMR6/RASA1/TRIP12/CSNK1A1/GDI1/PDS5B/AIMP1/MCF2/VTA1/AMBRA1/MAPK14/DUSP7/FAM126A/TBK1/IL18/CAPZA2/FOXO1/CNOT6/KLHL2/NLRC5/UBXN2B/DERA/ITCH/DNAJC3/DNAJC2/NANP/RNF146/CMAS/RELA/PCTP/CORO7/LYPLA1/STK3/PSMA6/USO1/TESK1/SDCBP/LRRK2/VPS26A/NRBP2/SNRPG/ME1/PACS1/TRAF1/TRAF2/FZR1/STAM2/RIOK1/PTRH2/RRAGB/NDRG1/NDRG2/AXIN2/UBE2D1/ARHGDIA/TEC/FGD4/PTPN7/CFLAR/VAV3/TBC1D10A/GALT/EVL/TRIM62/KCTD7/IKBKE/SARM1/PRKAR1A/RHOT1/FPGT/CRYZL1/KYNU/METAP2/AGTPBP1/DICER1/ECHDC1/ARHGAP19/EDC4/NIPSNAP3A/RGP1/FER/SKAP2/PRMT3/MKLN1/PSMD1/ARHGAP1/MKKS/SHC1/PSMD6/RHOF/CHUK/ZW10/SGK3/MINK1/STXBP3/ARHGAP29/IPO8/HMGA1/ATP6V1D/RFWD2/ATG4C/NUCB2/UCHL5/MDM2/UGP2/VPS29/PRKCZ/TNFRSF25/SNX16/PPM1A/PAXBP1/SEC62/TFAM/TUBGCP6/RASGRP2/HSPA4/INPP5E/PLCD1/APC/NFATC1/MTHFD2L/FLT3/CENPQ/RAF1/MEMO1/RALGDS/CDC25B/AMDHD2/ATXN3/SCFD1/IRF3/APAF1 |
| CC | GO:0016020/membrane | 149 | 1.67E-08 | ALS2/PLXNA3/SLC44A1/CHERP/IL9R/PDGFB/AP1G2/CAPZA2/PPP1R21/HBS1L/CNOT6/TAPBP/ST3GAL1/RNF141/FAM49B/AGPS/ITCH/ASPH/DNAJC3/PLD1/CMAS/UBIAD1/MED12/RPS6KC1/CORO7/VPS41/IL11RA/LPCAT4/PNPLA8/PNKP/IGSF8/CD37/HUWE1/MED15/TELO2/USO1/PCMTD1/SDCBP/SLU7/CPD/ARL8B/BIN1/MYBBP1A/XRN2/TPST1/ORAI1/AGFG2/ADORA2A/MAP4K1/SNX4/RFFL/FXYD5/PTRH2/CD74/CALU/ACE/PELP1/ITGB7/ITGAV/SLC30A5/PRPF40A/GIT1/GOLIM4/EVL/HGF/AK6/FXR1/LAT/LAMP2/ATP6V0E2/PRKAR1A/RHOT1/GRK6/RHEB/RIT1/IER3IP1/ATL2/LRRC8C/EDC4/NUP188/ALG6/RGP1/FLT3LG/DNAJC13/P4HA1/PSMD1/AGAP2/LBR/IFNGR1/NDUFS1/ZW10/AP3B1/ANO10/ADAM10/CNOT10/CDC5L/MAN1A1/LMBRD1/SLC9A3R1/LMBRD2/ATP6V1D/TMEM38B/CD163/SH2D3C/SEMA4F/DEF6/IPO4/SERPINB1/NKIRAS1/C16ORF58/GALNT3/PREP/PRKCZ/GALNT1/TNF/GALNT7/CSF1/LMF2/PPM1A/HCFC1/EEA1/KLC2/EDEM3/SEC62/ESYT1/SFT2D1/TUBGCP6/PPP2CA/NUP210/ABCD3/IL2RG/HSD17B4/VMP1/ZDHHC20/CD6/EHD1/ACSL4/SELPLG/CD5/ELMOD2/KCNE3/CSNK1A1/AIMP1/MCF2/ITPR2/SLC16A6/HSDL2/CSGALNACT2/ABCC1 |
| CC | GO:0005654/nucleoplasm | 170 | 1.45E-06 | XRCC4/FAM200B/SLC2A4RG/SLC44A1/PPP2R5D/MORF4L2/PTPN23/ASCC1/MED24/RORC/FOXO1/RBM7/CBX7/SLC26A11/INTS8/KLHL8/MIOS/SIN3A/MED29/GATA3/COL4A3BP/ORC4/DNAJC2/POLK/RARG/RXRB/CMAS/RELA/MED12/MTA1/RAD9A/UBR2/RMI1/BTBD8/PNKP/FAM222B/CEP162/HUWE1/PSMA6/MED15/DLD/BMP2K/MAPK9/SLU7/MYBBP1A/ZNF438/EP400/RAD17/XRN2/SNRPG/NMNAT1/HMGB1/FZR1/STAM2/SETD1B/ELK1/RRM2B/TRRAP/RFFL/RIOK1/MTIF2/DAZAP1/HNRNPA3/NIPBL/HNMT/PELP1/ZNF326/SYAP1/ISY1/POLM/SKIL/RCHY1/NDRG2/AXIN2/UBE2D1/RUNX1/PRPF40A/TCF7/PCIF1/TAF6/ZMYM3/KANSL3/GOLIM4/SMAD3/SKI/SMAD2/INTS10/AK6/BRCA1/UIMC1/PPIG/PHF1/SMARCC2/BTBD10/SCMH1/HDAC7/PPARD/KYNU/COPS5/COPS4/EDC4/RNF187/SKAP2/WBP4/JADE2/PSMD1/KIAA1033/NSMCE2/DPP8/PSMD6/CHUK/FANCB/ZCCHC8/POGZ/PAPD7/SF1/TP53/CCNC/CDC5L/IPO8/ZNF143/HMGA1/RFWD2/SENP2/RAB11FIP3/ZMIZ2/MDM2/CPSF3/THOC2/PRKD3/KIAA1429/KMT2D/USP8/KMT2A/KMT2B/UTP6/PPM1A/HAT1/HCFC1/CCDC59/POLR2A/ZBTB8OS/NR1D1/BCL9L/CC2D1B/GTF3C2/USP33/BAZ2A/ERCC4/TRIP12/NFATC1/APC/PDS5B/CENPQ/SMC6/FAM188A/SF3A2/IWS1/CDC25B/ATXN3/RNF44/PHF19/MAPK14/ATXN7/CABIN1/DGKZ/IRF3/MIS18BP1/CWC22/DUSP7 |
| CC | GO:0005737/cytoplasm | 270 | 3.26E-04 | FAM200B/SLC2A4RG/PTPN23/RNF217/RANGAP1/AQP3/TESPA1/HOOK3/SIN3A/CUL9/PHTF1/TXNL1/UBR3/RAD9A/UBR2/NME8/BTBD8/CEP162/MED15/HUWE1/ARL8B/BIN1/MYBBP1A/HSD17B11/ADSS/HSPBAP1/RFFL/HNRNPA3/HNMT/IDH1/RCHY1/OLFM2/FH/PRPF40A/GIT1/SRP54/MSTO1/ZC3H15/MAP2K4/FAM213B/SMAD3/SKI/SMAD2/PPA2/PTPN12/FNIP1/FXR1/CSPP1/BTBD10/PPP2R3C/DLEC1/LZTS2/COPS5/VBP1/RNF187/ST6GALNAC6/DNAJC13/SPG20/EEF2K/DPP8/FBXL16/USP15/ARHGEF1/ADAM10/POGZ/ARHGEF18/SNUPN/PAPD7/TP53/ELMO3/WDR47/ZDHHC17/BNIP2/SERPINB1/SIAH1/MOSPD1/SPOPL/MAP3K14/PREP/USP8/PPP6R1/ERI1/EEA1/KIN/TRIM11/FMO5/FAM117A/CIR1/NR1D1/RB1CC1/PPP2CB/UNK/LYPLAL1/MTMR6/ACSL4/USP33/KIF21B/BAZ2A/RASA1/TRIP12/KCNE3/GDI1/BRD2/AIMP1/RMDN2/AMBRA1/C21ORF2/MAPK14/DGKZ/CWC22/DRAM1/FAM126A/CHERP/XPO6/PLXNA1/ZAK/PDGFB/TBK1/PASK/FOXO1/CBX7/KLHL2/WNT1/NLRC5/MIOS/NUDCD2/NLRC3/LIX1L/ORC4/DERA/ITCH/DNAJC3/DNAJC2/RELA/UBIAD1/MTA1/RPS6KC1/LYPLA1/STK3/RNF130/TRIM35/PSMA6/FAM72B/TELO2/PCMTD1/SLU7/SDCBP/LRRK2/ZNF438/NRBP2/FBXO11/TRAF1/ZFAND6/TRAF2/STAM2/SETD1B/MAP4K1/SNX4/RRM2B/LLGL1/RRAGB/AZI2/DAZAP1/TTC1/IRAK3/SBK1/PELP1/PLCH2/SYAP1/NDRG1/NDRG2/SKIL/DDIAS/AXIN2/UBE2D1/RUNX1/ARHGDIA/FGD4/PTPN7/CFLAR/TAF6/ZMYM3/EVL/FOXP3/TRIM62/BRCA1/MPRIP/IKBKE/PPIG/SARM1/PHF1/PRKAR1A/ULK3/OVCA2/FPGT/HDAC7/HINT3/KYNU/METAP2/FAM175B/AGTPBP1/LRRC8C/DICER1/ESD/EDC4/CXCR3/FER/SKAP2/PRMT3/RMND5A/ZFYVE16/ARHGAP1/KLHL22/SV2A/LRWD1/CDK16/ZCCHC6/CHUK/ZW10/TBCE/MINK1/CDC5L/ARHGAP29/MAN1A1/SLC9A3R1/RFWD2/SH2D3C/ATG4C/IPO4/TBCD/ERN1/MDM2/HORMAD1/NKIRAS1/PRKD3/SPAST/UGP2/VPS29/PRKCZ/KMT2A/CRLF3/SSFA2/CPQ/HAT1/HCFC1/VCPKMT/DNAH1/RDX/TUBGCP6/CCDC125/PLCD1/APPBP2/TNPO2/ZC3H12D/NFATC1/APC/TMC6/TMC8/RAF1/ISOC1/RGS14/CDC25B/ATXN3/RNF44/ATXN7/CABIN1/IRF3/FEZ2/GCA/ACTR10 |
| CC | GO:0005794/Golgi apparatus | 59 | 5.52E-04 | IER3IP1/AP1G2/SGMS2/VPS54/PASK/SLC35A1/SLC26A11/RAB1A/HOOK3/ST3GAL1/ST6GALNAC6/EVI5/UBXN2B/COL4A3BP/ATP8B4/RAB27A/AP3B1/PLD1/RPGR/ADAM10/VPS45/ZDHHC8/MINK1/CORO7/MAN1A1/ZDHHC17/SCYL2/NUCB2/USO1/OSBPL11/LRRK2/GALNT3/TPST1/GALNT1/USP8/CPQ/SLC39A13/LMF1/ZDHHC18/TRRAP/RRAGB/CALU/SYAP1/SLC30A5/NDRG2/AXIN2/USP33/SRGN/FGD4/RAB2A/TMC6/GDI1/TMC8/AIMP1/GALT/GOLIM4/RAF1/FAM198B/CWC22 |
| CC | GO:0005813/centrosome | 33 | 1.75E-03 | ALS2/XRCC4/LZTS2/SDCCAG8/FAM175B/FBF1/HOOK3/TUBGCP6/KLHL22/MKKS/NDRG1/NDRG2/AXIN2/USP33/APC/CSNK1A1/RPGR/ROCK2/MED12/PIBF1/SKI/SLC9A3R1/ATP6V1D/RGS14/CDC25B/CSPP1/RFWD2/RAB11FIP3/CEP162/USP20/PPP2R3C/SPAST/IFT74 |
| CC | GO:0000922/spindle pole | 13 | 2.61E-03 | CSPP1/TUBGCP6/NUDCD2/FAM175B/RMDN2/PPP2CA/MAPK14/PPP2CB/RANGAP1/FBF1/RGS14/CDC25B/ZW10 |
| CC | GO:0016607/nuclear speck | 19 | 2.82E-03 | CSNK1A1/SRP54/SETD1B/SMC6/FAM76B/CDC5L/SF3A2/NSRP1/WBP4/RFWD2/CIR1/PPIG/SLU7/RCHY1/CWC22/THOC2/EP400/PRPF40A/KIAA1429 |
| CC | GO:0001772/immunological synapse | 7 | 3.04E-03 | LAT/CD37/STX7/CD3E/PRKAR1A/CD6/ARHGDIA |
| CC | GO:0005798/Golgi-associated vesicle | 5 | 3.22E-03 | SCFD1/ADAM10/AP1G2/VPS41/LRRK2 |
| CC | GO:0043231/intracellular membrane-bounded organelle | 39 | 3.85E-03 | VPS29/ALS2/HSD17B11/PLXNA3/STX7/STAM2/VBP1/RORC/HAT1/EDC4/RANGAP1/KIN/SLC26A11/RRAGB/AGPS/EVI5/DNAJC13/P4HA1/ZNF326/ZFYVE16/ABCD3/CC2D1B/HSD17B4/RUNX1/CHUK/POLK/ADAM10/KANSL3/RPS6KC1/MTA1/SLC9A3R1/MANBA/ZDHHC17/RAB11FIP3/BNIP2/FAM72B/SLU7/VPS26A/MYBBP1A |
| CC | GO:0005783/endoplasmic reticulum | 53 | 4.32E-03 | CYB5R4/IER3IP1/ATL2/SGPP2/SLC26A11/TAPBP/UBXN2B/P4HA1/POMT1/SERAC1/PHTF1/ASPH/SV2A/DNAJC3/PIEZO1/IFNGR1/ZW10/TOR1AIP2/MINPP1/PIK3C2B/UBIAD1/TP53/ERLIN1/MAN1A1/SIGMAR1/LPCAT4/MCTP1/SEMA4F/NUCB2/USO1/ERN1/LRRK2/UGGT2/SPAST/TMX1/PACS2/CPQ/KIAA0196/EDEM3/SEC62/CALU/FMO5/VMP1/TMC6/TMC8/AIMP1/FAM213B/FIG4/PLEKHF2/ATP2A3/SPCS3/MGST1/SEL1L |
| CC | GO:0005769/early endosome | 20 | 5.04E-03 | ALS2/VPS29/HMGB1/USP8/STX7/SGK3/SNX16/KIAA0196/PTPN23/RPS6KC1/EEA1/VPS41/RAB1A/VPS8/ZFYVE16/KIAA1033/SIAH1/EHD1/VPS26A/SNX13 |
| CC | GO:0010008/endosome membrane | 17 | 6.54E-03 | VPS29/AP1G2/TBK1/VPS45/VTA1/GOLIM4/SUN2/VPS41/RFFL/FIG4/IKBKE/ATP6V0E2/CHMP1B/DNAJC13/SCYL2/EHD1/VPS26A |
| CC | GO:0000151/ubiquitin ligase complex | 12 | 6.97E-03 | TRAF2/DCUN1D1/MED12/RNF217/UBR3/MED24/UBR2/RCHY1/UBE2D1/BRCA1/DCUN1D5/FBXO11 |
| CC | GO:0005778/peroxisomal membrane | 8 | 8.25E-03 | FAR1/TTC1/PNPLA8/AGPS/ABCD3/HSD17B4/ACSL4/MGST1 |
| CC | GO:0005789/endoplasmic reticulum membrane | 53 | 9.41E-03 | CYB5R4/IER3IP1/ATL2/SGPP2/LRRC8C/ALG6/RAB1A/TESPA1/TAPBP/SLC35D1/POMT1/COL4A3BP/ASPH/PCYT2/PIEZO1/RAB21/ZW10/TOR1AIP2/PLD1/SPTLC1/JKAMP/UBIAD1/ERLIN1/SIGMAR1/LPCAT4/PNPLA8/RAB18/ERN1/SDCBP/SPAST/TMX1/GALNT1/LMF2/LMF1/TMX3/HSD17B12/TMEM259/SEC62/CALU/FMO5/NUP210/ACSL4/RAB2A/TMC6/TMC8/ITPR2/ATXN3/SCFD1/ATP2A3/SPCS3/RHEB/MGST1/SEL1L |
| CC | GO:0000139/Golgi membrane | 39 | 9.52E-03 | TPST1/GALNT3/GALNT1/GALNT7/PDGFB/AP1G2/NDST2/UNC50/PPP6R1/MAP4K2/RFFL/RGP1/SLC35A1/CD74/RAB1A/TAPBP/ST3GAL1/ST6GALNAC6/LMTK3/SRGN/RAB2A/PLD1/ADAM10/TMEM167A/ROCK1/VPS45/GOLIM4/CORO7/MAN1A1/FIG4/TRIM23/RHBDD2/RFWD2/PNPLA8/ZDHHC17/FAM198B/CSGALNACT2/USO1/RHEB |
| CC | GO:0000790/nuclear chromatin | 17 | 9.67E-03 | PPARD/RARG/POGZ/RELA/LDB1/TP53/HAT1/SMAD3/SMAD2/FER/CBX7/NR1D1/GATA3/IPO4/SMARCC2/RAD17/NFATC1 |
| CC | GO:0005622/intracellular | 76 | 1.05E-02 | EDN3/ZAK/XPO6/HBS1L/RAB1A/TRIM46/NUDCD2/CLCF1/MFHAS1/MKKS/SHC1/AGAP2/RHOF/PAG1/ANO9/CHUK/ANO10/RAB27A/ROCK1/ROCK2/PIK3CB/PIK3C2B/PIK3CD/VPS41/IPO8/ARHGAP29/SH2D3C/PNPLA8/TRIM35/SH2D3A/RAB18/IPO4/TELO2/UCHL5/SDCBP/MAPK9/ARL8B/LRRK2/EEFSEC/NKIRAS1/PTGDR2/PRKD3/TRAF2/PRKCZ/MAP4K2/MAP4K1/CD74/GFM2/PLCH2/ITGAV/SOS2/RASGRP2/IL2RG/PLCD1/TNPO2/ZNF267/RAB2A/GDI1/SRP54/VAV3/MCF2/RAF1/SMAD3/SMC6/RALGDS/RGS14/RABL2B/CDC25B/HEATR3/MAPK14/RHOT1/RHEB/DGKZ/RIT1/ZNF764/SYTL1 |
| CC | GO:0031965/nuclear membrane | 19 | 1.06E-02 | TOR1AIP2/TMC6/FZR1/TMC8/SUN2/FAM188A/RANGAP1/FAM76B/TMEM38B/SENP2/ATP2A3/RB1CC1/IPO4/NUP210/NUP54/TNPO2/DNAJC2/LBR/SPAST |
| CC | GO:0031901/early endosome membrane | 12 | 1.17E-02 | PLEKHF2/STX7/DNAJC13/STAM2/ZFYVE16/SNX16/EEA1/SNX4/FIG4/EHD1/SNX13/LLGL1 |
| CC | GO:0005765/lysosomal membrane | 21 | 1.57E-02 | RAB2A/PLD1/STX7/GDAP2/SNX14/TMEM63A/VPS41/TRIM23/LMBRD1/SLC26A11/ATP6V1D/CD74/RRAGB/ATP6V1C1/LAMP2/MIOS/DNAJC13/RHEB/ARL8B/DRAM1/AP3B1 |
| CC | GO:0035097/histone methyltransferase complex | 5 | 1.59E-02 | KMT2D/KMT2A/SETD1B/KMT2B/ZNF335 |
| CC | GO:0000123/histone acetyltransferase complex | 5 | 1.59E-02 | JADE2/KANSL3/HCFC1/KANSL1L/TRRAP |
| CC | GO:0044322/endoplasmic reticulum quality control compartment | 4 | 1.66E-02 | EDEM3/UGGT2/ERLEC1/SEL1L |
| CC | GO:0071013/catalytic step 2 spliceosome | 10 | 1.78E-02 | HNRNPA3/CWC27/ISY1/SYF2/SLU7/CDC5L/SF3A2/CWC22/SNRPG/ZCCHC8 |
| CC | GO:0030877/beta-catenin destruction complex | 4 | 2.04E-02 | CSNK1A1/SIAH1/AXIN2/APC |
| CC | GO:0000974/Prp19 complex | 4 | 2.04E-02 | ISY1/SYF2/CDC5L/POLR2A |
| CC | GO:0005635/nuclear envelope | 14 | 2.05E-02 | PRKCZ/SUN2/MTA1/RANGAP1/NUP188/IPO8/SIGMAR1/BNIP2/NUP210/NUCB2/NUP54/BIN1/MTMR6/LBR |
| CC | GO:0016363/nuclear matrix | 10 | 2.43E-02 | PRKCZ/ATXN3/PPIG/PSMA6/ZNF326/ATXN7/TP53/HAT1/KIN/PRPF40A |
| CC | GO:0005634/nucleus | 260 | 2.55E-02 | XRCC4/SLC2A4RG/PPP2R5D/PTPN23/ASCC1/MED24/RORC/NSRP1/AQP3/SFSWAP/SIN3A/ZSCAN9/TRMT1L/PHTF1/TXNL1/POLK/RARG/PCBD2/PIK3CB/RXRB/CIZ1/ROCK2/MED12/RAD9A/BTBD8/PRDM8/PNKP/MED15/DHX29/HUWE1/EEFSEC/MYBBP1A/ZNF76/HNRNPA3/POLM/RCHY1/OLFM2/FRA10AC1/SRP54/TCF7/KANSL3/MAP2K4/SMAD3/SKI/PIBF1/SMAD2/RPF2/BTBD10/KLF2/PPP2R3C/ATAD2B/RANBP10/PPARD/COPS5/COPS4/VBP1/ZEB2/RNF187/ZNF518A/AES/PBXIP1/EVI5/NSMCE2/AGAP2/USP15/ADAM10/POGZ/ZC3H7B/PIK3C2B/SNUPN/CNOT10/ANP32E/ZNF142/PAPD7/SF1/TP53/FAM76B/CCNC/ZNF335/BRWD3/ZNF277/SIAH1/MOSPD1/SPOPL/IFT74/PREP/LCORL/ERI1/BCCIP/CIC/KIN/POLR2A/TRIM11/SFR1/SORBS3/CIR1/NR1D1/BCL11B/PPP2CA/PPP2CB/BCL9L/CC2D1B/USP36/MLLT6/BAZ2B/BAZ2A/TRIP12/ZNF267/NEMF/RAB2A/BRD2/PDS5B/AIMP1/SF3A2/TRIM23/IWS1/SNAI3/ZNF672/MAPK14/SYF2/ZBTB4/DGKZ/ZNF764/PBX4/PLXNA3/XPO6/PLXNA1/ZAK/MORF4L2/PASK/FOXO1/RBM7/CBX7/CNOT6/NLRC5/MIOS/UBXN2B/GATA3/ORC4/DERA/ITCH/DNAJC2/RNF146/CMAS/RELA/UBIAD1/MTA1/RMI1/STK3/RNF130/DCUN1D1/TRIM35/PSMA6/TELO2/BMP2K/SLU7/SDCBP/SWT1/CPD/ZNF438/RAD17/XRN2/FBXO11/NMNAT1/HMGB1/SETD1B/ELK1/TRRAP/RRAGB/ZNF330/DAZAP1/IRAK3/NIPBL/PELP1/ZNF326/SLC30A5/NDRG1/SKIL/DDIAS/AXIN2/RUNX1/SLC30A9/ARHGDIA/TAF6/FOXP3/BRCA1/UIMC1/FAM208A/IKBKE/PPIG/PHF1/DYRK1B/OVCA2/ZNF319/ZNF414/SCMH1/HDAC7/HINT3/POU6F1/FOXK1/AGTPBP1/DICER1/ARHGAP19/NIPSNAP3A/EDC4/FER/RMND5A/PSMD1/LRWD1/ZW10/ZCCHC8/LDB1/CDC5L/SLC9A3R1/HMGA1/TMEM38B/SBF1/ZMIZ2/DEF6/UCHL5/MDM2/HORMAD1/PRKD3/SPAST/UGP2/KMT2D/KMT2A/SSFA2/KMT2B/PPM1A/HAT1/HCFC1/PAXBP1/CCDC59/TFAM/APPBP2/RSL24D1/TNPO2/PHF20L1/ERCC4/ZC3H12D/NFATC1/APC/MORN2/FLT3/SMC6/RAF1/MEMO1/RALGDS/RGS14/RUFY2/ATXN3/AMDHD2/ATXN7/CABIN1/MAMLD1/IRF3/APAF1/TIAF1/MGST1 |
| CC | GO:0030173/integral component of Golgi membrane | 7 | 3.84E-02 | ST3GAL1/IER3IP1/SGMS2/SLC39A13/UNC50/CSGALNACT2/UBIAD1 |
| CC | GO:0001726/ruffle | 9 | 4.06E-02 | ALS2/MKLN1/ROCK1/INPP5E/RDX/SLC9A3R1/RASA1/KLHL2/FGD4 |
| CC | GO:0032580/Golgi cisterna membrane | 8 | 4.55E-02 | GALNT3/ST3GAL1/GALNT1/SCFD1/CSGALNACT2/GOLIM4/INPP5E/RAB21 |
| CC | GO:0000781/chromosome, telomeric region | 6 | 4.73E-02 | TELO2/HAT1/NSMCE2/SMC6/ERCC4/RAD17 |
| MF | GO:0005515/protein binding | 481 | 5.40E-13 | ALS2/XRCC4/PPP2R5D/VPS54/LTBP4/ASCC1/MED24/RORC/RANGAP1/NSRP1/INTS8/ZSCAN9/MED29/BTBD1/CUL9/RAB27A/ADAM9/PCBD2/ROCK1/RXRB/PIK3CB/ROCK2/VPS45/PIK3CD/MED12/VPS41/ZUFSP/MARK3/MED15/SCYL2/TRAPPC8/MAPK9/ARL8B/MYBBP1A/ADSS/ORAI1/HSPBAP1/HSD17B12/PRRC2B/ISY1/GCSH/RCHY1/PRPF40A/FH/FRA10AC1/GIT1/TCF7/ZC3H15/MAP2K4/SPTSSA/PIBF1/HGF/FNIP1/LACTB2/FXR1/SYTL1/PPP2R3C/LZTS2/RNF187/PBXIP1/EVI5/P4HA1/SLMAP/SPG20/IL13RA1/USP15/ARHGEF1/ZC3H7B/PIK3C2B/CNOT10/TP53/ERLIN1/ZNF143/ELMO3/ZNF335/VPS8/WDR47/BNIP2/USP20/SPOPL/THOC2/CSF1/C5/BCCIP/EEA1/FBF1/TRIM11/SORBS3/SFR1/PPP2CA/PPP2CB/SOS2/LIMD2/EXOC6/VMP1/MTMR6/USP33/KCNE3/TRIP12/RASA1/ETFA/BRD2/AIMP1/RMDN2/VTA1/TRIM23/SF3A2/PHF19/PLEKHF2/C21ORF2/PLSCR3/SYF2/ZBTB4/DGKZ/ZNF764/MEGF6/CWC22/DUSP7/CHERP/XPO6/ZAK/AP1G2/IL18/PASK/CNOT6/NLRC5/FAM49B/NUDCD2/MFHAS1/UBXN2B/SMARCD1/ASPH/RNF146/SPTLC1/RELA/PCTP/MTA1/RPS6KC1/CORO7/RMI1/DCUN1D1/TELO2/TESK1/UGGT2/LRRK2/XRN2/EP400/DCUN1D5/FBXO11/NMNAT1/TRAF1/HMGB1/TRAF2/STX7/ADORA2A/STAM2/SETD1B/PTRH2/AZI2/ZNF330/TTC1/NIPBL/ZNF326/SYAP1/ARHGDIA/TEC/VAV3/CCPG1/TAF6/CACNA1I/CYB561/UIMC1/MPRIP/LAT/IKBKE/LAMP2/SARM1/DYRK1B/SMARCC2/PRKAR1A/ZNF414/HDAC7/SEL1L/ATL2/LRRC8C/DICER1/NIPSNAP3A/EDC4/FER/RGP1/SKAP2/PRMT3/LACE1/CLCF1/ZFYVE16/SUCLA2/LRWD1/LBR/NDUFS1/ZCCHC8/SIT1/SGK3/STXBP3/CDC5L/IPO8/SLC9A3R1/ATP6V1D/HMGA1/TIMM21/RFWD2/SH2D3C/SH2D3A/DEF6/TBCD/IPO4/UCHL5/ERN1/CPSF3/UGP2/VPS29/KMT2D/KMT2A/GDAP2/CRLF3/KMT2B/PPM1A/RDX/ESYT1/CCDC120/TFAM/HSPA4/APPBP2/CD6/EHD1/CD5/SELPLG/NFATC1/TMC6/MORN2/TMC8/FLT3/SMC6/RAF1/GDPD5/FIG4/MEMO1/CDC25B/SCFD1/AMDHD2/APAF1/MIS18BP1/FEZ2/GCA/PTPN23/RAB1A/ERLEC1/HOOK3/SFSWAP/C1ORF109/SIN3A/AGPS/COL4A3BP/RAB21/PLD1/RARG/CD3E/CIZ1/RAD9A/UBR2/PRDM8/PNKP/CD37/TRAPPC13/CEP162/HUWE1/RAB18/FBXL8/BIN1/TMX3/RFFL/CD74/CALU/HNRNPA3/FAM102A/R3HCC1L/POLM/NUP54/OLFM2/FCHO2/SRP54/NPL/SMAD3/SKI/SMAD2/PTPN12/TAX1BP1/GRK6/RIT1/KLF2/PPARD/COPS5/SLC15A2/PHKB/COPS4/VBP1/ZEB2/WBP4/AES/DNAJC13/NSMCE2/AGAP2/FANCB/ADAM10/RPGR/POGZ/SF1/CCNC/DNAJC21/CBR4/FAM76B/LENG8/CD163/SENP2/ATP6V1C1/ZDHHC17/RAB11FIP3/CHMP1B/SIAH1/MAP3K14/PREP/SDCCAG8/LCORL/USP8/SLC39A13/KIAA0196/PPP6R1/KLC2/CIC/POLR2A/CIR1/NR1D1/MRPL15/BCL11B/RB1CC1/ABCD3/CC2D1B/MLLT6/BAZ2B/BAZ2A/RAB2A/CSNK1A1/GDI1/PDS5B/MCF2/NLGN3/AMBRA1/IWS1/MAPK14/DRAM1/FAM126A/RBM17/PBX4/PLXNA3/PDGFB/TBK1/MORF4L2/FOXO1/CBX7/KLHL2/TAPBP/MIOS/GATA3/ANKRD50/ORC4/ITCH/PAG1/UBIAD1/TNFRSF14/GSE1/STK3/IGSF8/PSMA6/USO1/SLU7/SDCBP/VPS26A/RAD17/SNRPG/PACS1/ZFAND6/FZR1/MAP4K2/ELK1/MAP4K1/SNX4/NAPB/RRM2B/TRRAP/RIOK1/LLGL1/RRAGB/FXYD7/GFM2/PELP1/ITGB7/ITGAV/NDRG1/NDRG2/SKIL/RUNX1/UBE2D1/AXIN2/SRGN/PTPN7/CFLAR/SESTD1/TBC1D10A/GALT/EVL/ITGA3/INTS10/FOXP3/AK6/BRCA1/TBC1D23/PPIG/PHF1/UCP2/TXNDC16/ULK3/RHOT1/SCMH1/FOXK1/ESD/JADE2/RMND5A/CCDC43/ARHGAP1/KLHL22/MKKS/CNTNAP1/SHC1/CDK16/PSMD6/IFNGR1/CHUK/ZW10/LDB1/MINK1/IFNAR1/ZMIZ2/NUCB2/MDM2/SPAST/PRKD3/PRKCZ/TNF/LMF2/HCFC1/HAT1/VCPKMT/CCDC59/IL2RG/RSL24D1/PHF20L1/TNPO2/ERCC4/ZC3H12D/APC/NDUFA4/SUN2/FAM188A/ISOC1/RALGDS/RGS14/ATXN3/ATXN7/CSGALNACT2/IRF3/MGST1/ACTR10 |
| MF | GO:0003682/chromatin binding | 34 | 2.62E-04 | ENY2/KMT2A/HCFC1/FOXO1/ELK1/CIC/CBX7/TFAM/NIPBL/SIN3A/PELP1/GATA3/SMARCD1/SKIL/LRWD1/DNAJC2/SLC30A9/BRD2/LDB1/RELA/TP53/MED12/MTA1/SKI/SMAD2/HMGA1/PHF1/ATXN7/SMARCC2/RAD17/EP400/HDAC7/ATAD2B/IFT74 |
| MF | GO:0008270/zinc ion binding | 77 | 3.58E-04 | PPARD/AGTPBP1/RORC/RNF187/WBP4/TRIM46/JADE2/RNF141/GATA3/CUL9/NSMCE2/ZCCHC6/RNF146/ZCCHC8/RARG/CIZ1/RXRB/ZDHHC8/TP53/SF1/UBR3/MTA1/VPS41/UBR2/DNAJC21/VPS8/RFWD2/RNF130/ZDHHC17/TRIM35/ZMIZ2/USP20/SLU7/MDM2/SIAH1/CPD/RNF26/FBXO11/TRAF1/ZFAND6/ADAMTS17/TRAF2/KMT2D/KMT2A/KMT2B/ERI2/EEA1/ZDHHC18/RFFL/ZFAND1/ZNF330/TRIM11/ACE/NR1D1/CDADC1/LIMD2/SLC30A5/RCHY1/ZDHHC20/MLLT6/BAZ2B/USP33/BAZ2A/ZMYM3/GALT/SMAD3/SKI/TRIM23/SF3A2/TRIM62/LACTB2/BRCA1/KCTD7/RNF44/PHF19/PHF1/CRYZL1 |
| MF | GO:0031625/ubiquitin protein ligase binding | 26 | 8.99E-04 | TRAF1/TRAF2/FOXO1/RANGAP1/RFFL/POLR2A/BTBD1/CUL9/SPG20/AXIN2/JKAMP/RELA/TP53/SMAD3/SMC6/SMAD2/SKI/TNFRSF14/ERLIN1/AMBRA1/BRCA1/IKBKE/ATXN3/PRKAR1A/MDM2/SPOPL |
| MF | GO:0008565/protein transporter activity | 11 | 1.14E-03 | VPS29/XPO6/AP1G2/ZFYVE16/IPO4/SNUPN/USO1/IPO8/TNPO2/SEC62/VPS26A |
| MF | GO:0004672/protein kinase activity | 30 | 1.21E-03 | PRKCZ/FASTKD1/TBK1/PASK/MAP4K2/MAP4K1/COL4A3BP/EEF2K/LMTK3/CDK16/CHUK/CSNK1A1/SGK3/ROCK1/MAP2K4/RAF1/MINK1/RPS6KC1/STK3/IKBKE/SCYL2/MAPK14/DYRK1B/ULK3/BMP2K/TESK1/MAPK9/LRRK2/MAP3K14/NRBP2 |
| MF | GO:0004674/protein serine/threonine kinase activity | 31 | 1.22E-03 | PRKCZ/ZAK/TBK1/PASK/MAP4K2/MAP4K1/RIOK1/IRAK3/SBK1/EEF2K/LMTK3/CDK16/CSNK1A1/SGK3/ROCK1/ROCK2/RPS6KC1/RAF1/MINK1/CCNC/MARK3/STK3/MAPK14/DYRK1B/ULK3/ERN1/BMP2K/TESK1/MAP3K14/LRRK2/NRBP2 |
| MF | GO:0003924/GTPase activity | 22 | 1.55E-03 | RAB2A/SRP54/MSTO1/ATL2/HBS1L/TRIM23/MTIF2/RRAGB/RAB1A/RABL2B/GFM2/RAB18/RHOT1/RHEB/RIT1/ARL8B/LRRK2/EEFSEC/NKIRAS1/RHOF/RAB21/RAB27A |
| MF | GO:0035091/phosphatidylinositol binding | 11 | 4.00E-03 | PLD1/SGK3/SNX14/PIK3C2B/SNX16/PASK/RPS6KC1/SNX4/SNX13/FCHO2/ITPR2 |
| MF | GO:0004842/ubiquitin-protein transferase activity | 26 | 5.60E-03 | TRAF2/RNF217/RNF187/KLHL2/KLHL8/RNF141/RMND5A/KLHL22/ITCH/RCHY1/UBE2D1/RNF146/TRIP12/UBR3/UBR2/TRIM23/TRIM62/BRCA1/RFWD2/RNF130/HUWE1/FBXL8/WDSUB1/MDM2/SIAH1/FBXO11 |
| MF | GO:0005085/guanyl-nucleotide exchange factor activity | 13 | 5.67E-03 | ALS2/RPGR/VAV3/MCF2/TBC1D10A/ARHGEF18/FNIP1/RALGDS/SH2D3C/SH2D3A/SOS2/RASGRP2/FGD4 |
| MF | GO:0005070/SH3/SH2 adaptor activity | 8 | 1.09E-02 | LAT/SH2D3C/VAV3/SH2D3A/ARHGAP1/CNTNAP1/SKAP2/PAG1 |
| MF | GO:0016874/ligase activity | 21 | 1.59E-02 | XRCC4/TRAF2/RNF217/UBR3/RNF187/UBR2/RFFL/TRIM23/TRIM62/BRCA1/TRIM11/RFWD2/RNF130/HUWE1/MDM2/NSMCE2/SIAH1/RCHY1/SUCLA2/RNF146/TRIP12 |
| MF | GO:0004896/cytokine receptor activity | 6 | 1.96E-02 | IL9R/FLT3/IL2RG/IL13RA1/IL11RA/CD74 |
| MF | GO:0001047/core promoter binding | 8 | 2.17E-02 | TCF7/MTA1/ELK1/KANSL1L/RUNX1/FOXP3/MYBBP1A/POLR2A |
| MF | GO:0005096/GTPase activator activity | 21 | 2.19E-02 | GIT1/GDI1/ARHGEF1/VAV3/TBC1D10A/AGFG2/ARHGAP19/RANGAP1/ARHGAP29/LLGL1/RGS14/EVI5/BNIP2/TBCD/ARHGAP1/AXIN2/LRRK2/AGAP2/RASA1/ARHGDIA/ELMOD2 |
| MF | GO:0030374/ligand-dependent nuclear receptor transcription coactivator activity | 7 | 2.36E-02 | ENY2/SFR1/ZMIZ2/MED12/MED24/HMGA1/SLC30A9 |
| MF | GO:0016779/nucleotidyltransferase activity | 5 | 2.89E-02 | PAPD7/PCYT2/ZCCHC6/FPGT/NMNAT1 |
| MF | GO:0019003/GDP binding | 7 | 3.03E-02 | RAB2A/SRP54/RAB18/ARL8B/TRIM23/RAB21/RAB27A |
| MF | GO:0005525/GTP binding | 26 | 3.25E-02 | RAB2A/ADSS/SRP54/ATL2/HBS1L/MTIF2/TRIM23/RAB1A/RRAGB/RABL2B/GFM2/RAB18/MFHAS1/RHOT1/RHEB/RIT1/ARL8B/EEFSEC/EHD1/LRRK2/AGAP2/FPGT/RHOF/NKIRAS1/RAB21/RAB27A |
| MF | GO:0035005/1-phosphatidylinositol-4-phosphate 3-kinase activity | 3 | 3.49E-02 | PIK3CB/PIK3C2B/PIK3CD |
| MF | GO:0019208/phosphatase regulator activity | 3 | 3.49E-02 | SBF1/BMP2K/ZEB2 |
| MF | GO:0017124/SH3 domain binding | 11 | 3.65E-02 | ADAM10/USP8/CD3E/PLSCR3/ARHGAP1/CNTNAP1/RAD9A/EVL/ELMO3/PTPN12/ADAM9 |
| MF | GO:0008536/Ran GTPase binding | 5 | 4.08E-02 | XPO6/IPO4/RANGAP1/IPO8/TNPO2 |
| MF | GO:0016409/palmitoyltransferase activity | 4 | 4.20E-02 | ZDHHC17/ZDHHC8/ZDHHC18/ZDHHC20 |
| MF | GO:0070577/lysine-acetylated histone binding | 4 | 4.20E-02 | BRD2/KMT2A/BAZ2A/ATAD2B |
| MF | GO:0042800/histone methyltransferase activity (H3-K4 specific) | 4 | 4.20E-02 | KMT2D/KMT2A/SETD1B/KMT2B |
| MF | GO:0019901/protein kinase binding | 25 | 4.32E-02 | TRAF2/PRKCZ/PTPN23/RFFL/LLGL1/RB1CC1/SV2A/DNAJC3/AXIN2/APC/ADAM10/CD3E/RELA/TP53/SMAD3/SKI/RAD9A/CDC5L/RGS14/CDC25B/LAT/TELO2/ZBTB4/RHEB/HDAC7 |
| MF | GO:0004521/endoribonuclease activity | 4 | 4.83E-02 | DICER1/ERN1/CPSF3/LACTB2 |

Supplementary Table 3 GO enrichment and DEGs at 1 month after STEMI

| Category | Term | Count | PValue | Genes |
| --- | --- | --- | --- | --- |
| BP | GO:0045892~negative regulation of transcription, DNA-templated | 46 | 1.83E-04 | XRCC5/BTAF1/SBNO2/PPARD/GCLC/SNX6/ZNF639/ARHGAP35/ELK3/ZNF253/RLIM/TRIM11/AES/NIPBL/NR1D1/PBXIP1/WWP2/TSC22D4/PCGF6/GATAD2A/RARA/BHLHE40/RUNX1/CCAR2/BAHD1/ZBTB7A/NACC1/LDB1/L3MBTL1/TP53/HDAC10/MECP2/RB1/MBD1/ATXN1/HDAC4/NOTCH1/CHMP1A/HDAC2/PIAS4/YAF2/UBA3/SMARCC2/ZNF746/SMARCA4/HDAC6 |
| BP | GO:0007249~I-kappaB kinase/NF-kappaB signaling | 12 | 2.12E-04 | TRAF2/TNFRSF1A/TBK1/LY96/ZNF675/NFKB2/MAP3K14/BIRC2/TIAF1/AZI2/CHUK/RNF31 |
| BP | GO:0015031~protein transport | 38 | 3.25E-04 | VPS29/ARFGAP1/SNX14/PLEKHM1/SNX4/SNX3/ARF5/SFT2D1/GBF1/NUP210/EXOC6/RAB21/SEC61A1/GPR89A/AP5Z1/RAB2A/GDI1/ACTN4/VTA1/RAB4A/CORO7/GABARAP/ANKRD27/TMEM115/PITPNM1/SCFD1/CHMP1A/PLEKHF2/ATG4D/ARHGAP33/RAB18/ATG4C/RAB35/CCDC53/ARF4/RAP1A/COG2/MVP |
| BP | GO:0051056~regulation of small GTPase mediated signal transduction | 18 | 5.19E-04 | GDI1/ARHGEF2/ABR/BCR/ARHGAP35/MYO9B/ARHGAP4/ARHGAP33/RAC2/GMIP/TSC2/RHOT1/RHOT2/RAP1GAP2/RHOF/ARAP1/ARHGDIA/FGD3 |
| BP | GO:0006367~transcription initiation from RNA polymerase II promoter | 19 | 8.28E-04 | TAF4/PPARD/RARG/TAF6/RXRB/RXRA/CREBBP/MED12/MED24/CCNC/GTF2B/POLR2A/TAF11/NOTCH1/NR1D1/MED15/MED16/GTF2A2/RARA |
| BP | GO:0007264~small GTPase mediated signal transduction | 26 | 9.25E-04 | ARFRP1/RAB40C/ARHGAP35/ARF5/RAC2/MFHAS1/RASGRP2/RHOF/RAB21/RAB2A/GDI1/ABR/RAB4A/RABL2B/SH2D3C/ARHGAP33/RAB18/RAB35/ARF4/RHOT1/RHEB/RAP1A/RHOT2/RAP1B/ARL8B/TNK2 |
| BP | GO:0006511~ubiquitin-dependent protein catabolic process | 21 | 1.13E-03 | USP8/USP5/UBE2G1/BAP1/UBR3/RLIM/PSMA2/CUL3/PSMA1/USP19/CUL5/ATXN3/BAG6/PSMA6/CUL7/CUL9/PSMA4/PSMA3/USP21/USP35/USP16 |
| BP | GO:0051146~striated muscle cell differentiation | 5 | 1.14E-03 | AKT1/BNIP2/MAPK14/RB1/CHUK |
| BP | GO:0006886~intracellular protein transport | 25 | 1.15E-03 | VPS29/AP1G2/SNX6/STAM2/SNX2/AP3S1/TSNARE1/TBC1D15/TBC1D13/AP3D1/VPS35/EHD1/KDELR1/VPS18/ARHGEF2/VPS45/COPG1/SGSM2/TOM1L2/IPO5/HGS/GGA1/SNX13/GGA3/HDAC6 |
| BP | GO:0016032~viral process | 29 | 1.66E-03 | PACS1/PACS2/DYNC1LI1/AP1G2/WASF2/RBX1/TNFRSF1A/CUL5/GBF1/CUL7/RAE1/NUP210/SND1/GTF2A2/SHC1/NUP54/TAF4/CREBBP/TP53/RB1/NUPL2/LMBRD1/GTF2B/VDAC1/PSMA4/PSMA3/IPO5/TSC2/SRCAP |
| BP | GO:0048384~retinoic acid receptor signaling pathway | 6 | 1.74E-03 | ALDH1A2/RARG/ACTN4/RXRB/RXRA/RARA |
| BP | GO:0031175~neuron projection development | 14 | 1.82E-03 | HMGB1/TBC1D24/PPP1R9B/ATXN10/PTK2B/RAB35/USP21/MAP4/GDPD5/RB1/MKL1/EHD1/SRF/CAPZB |
| BP | GO:0038061~NIK/NF-kappaB signaling | 11 | 1.90E-03 | PSMA2/PSMA1/PSMA6/PSMD10/PSMA4/UBA3/PSMA3/NFKB2/MAP3K14/BIRC2/CHUK |
| BP | GO:0000122~negative regulation of transcription from RNA polymerase II promoter | 56 | 2.23E-03 | PPARD/CNOT2/ZNF675/NFKB2/RLIM/CUL3/N4BP2L2/SIN3B/AES/PEX2/WWP2/PCGF6/RARA/RBM10/RARG/HIST1H1C/RXRA/TP53/HDAC10/MECP2/ARID1A/RB1/MBD1/NOC2L/MXD4/PIAS4/RFC1/MTF2/ZNF746/SMARCA4/HMGB1/CHCHD3/ARHGAP35/CIC/SORBS3/NIPBL/NR1D1/ATN1/JUND/GATAD2A/CC2D1B/BHLHE40/TCF3/ZBTB7A/WDTC1/CREBBP/EHMT2/HDAC4/NOTCH1/PHF19/HDAC2/PSMD10/SMARCC2/LRP8/NCOR2/HDAC7 |
| BP | GO:0030036~actin cytoskeleton organization | 16 | 2.81E-03 | ABR/BCR/TAOK2/DIAPH1/LIMK1/WASF2/PIP5K1C/SLC9A3R1/CAPZB/ATXN3/RAC2/FLII/TMOD3/MKL1/IQSEC1/FGD3 |
| BP | GO:0006890~retrograde vesicle-mediated transport, Golgi to ER | 12 | 3.11E-03 | ARFGAP1/TMEM115/KIF1C/SCFD1/COPG1/GBF1/ARF4/ARF5/KLC2/KDELR1/ERGIC2/TAPBP |
| BP | GO:0071260~cellular response to mechanical stimulus | 11 | 3.30E-03 | AKT1/TNFRSF1A/HDAC4/GCLC/PTGER4/MAPK8/ATP1A1/MAP3K14/CASP2/ARHGDIA/SLC9A1 |
| BP | GO:0045116~protein neddylation | 5 | 3.43E-03 | UBA3/DCUN1D4/DCUN1D5/NAE1/RBX1 |
| BP | GO:0016180~snRNA processing | 5 | 3.43E-03 | TUT1/INTS8/INTS1/INTS3/INTS10 |
| BP | GO:0006914~autophagy | 16 | 3.50E-03 | HMGB1/VPS18/PLEKHM1/BECN1/STK11/STAM2/VTA1/PTPN22/UBQLN4/GABARAP/DRAM2/ATG4D/ATG4C/MAP1S/ARSA/HGS |
| BP | GO:0045893~positive regulation of transcription, DNA-templated | 42 | 3.72E-03 | MMS19/PPARD/PTGES2/ELF4/ZXDC/MED24/ELK3/SFR1/NR1D1/RARA/RUNX1/USP16/DNAJC2/TCF3/DVL2/IRAK1/DVL3/TAF4/MAP2K2/CREBBP/TP53/MED12/MECP2/ARID1A/RB1/IFNAR1/DVL1/HDAC4/NOTCH1/HDAC2/HIF1A/RFC1/ANKRD49/YAF2/MED16/DYRK1B/SMARCC2/USP21/NFE2L1/KLF2/SMARCA4/DNM2 |
| BP | GO:0043547~positive regulation of GTPase activity | 45 | 4.11E-03 | ARFGAP1/AGFG2/PREX1/CYTH4/ARHGAP35/MYO9B/RGP1/LLGL1/ARHGAP4/GBF1/GMIP/RASGRP2/SHC1/RAP1GAP2/FGD3/ARHGDIA/IQSEC1/DVL2/GIT1/DVL3/GDI1/ARHGEF2/ARHGEF1/BCR/ABR/PSD4/RGS14/RIC8A/ANKRD27/TBC1D24/SH2D3C/FNBP1/ARHGAP33/ACAP3/SGSM2/SBF1/BNIP2/ARF4/TSC2/RAP1A/JAK3/DENND4B/SH3BP1/SNX13/ARAP1 |
| BP | GO:0006406~mRNA export from nucleus | 13 | 5.21E-03 | SRSF3/UPF3B/UPF1/SRSF10/SMG6/SMG5/RAE1/NUP210/THOC7/CASC3/NUP54/CPSF3/NUPL2 |
| BP | GO:0016569~covalent chromatin modification | 14 | 5.34E-03 | BRD2/L3MBTL1/ARID1A/ARID1B/RB1/BAG6/PHF19/MTF2/SMARCD1/SMARCC2/CABIN1/DNAJC2/SMARCA4/BAHD1 |
| BP | GO:0008360~regulation of cell shape | 16 | 5.66E-03 | CSNK1A1/FMNL1/TAOK2/DIAPH1/ANXA1/ARHGAP35/ITGB2/MYL12A/SLC9A3R1/SYNE3/VRK1/C21ORF2/PTK2B/FGD3/ARHGDIA/ARAP1 |
| BP | GO:0043161~proteasome-mediated ubiquitin-dependent protein catabolic process | 20 | 8.68E-03 | CSNK1A1/ANAPC5/TP53/CDC16/RLIM/BIRC2/RBX1/PSMA2/CUL3/PSMA1/ATXN3/FBXW5/PSMA6/BTBD1/WWP2/PSMD10/PSMA4/PPP2CB/PSMA3/HECTD3 |
| BP | GO:0048096~chromatin-mediated maintenance of transcription | 4 | 9.03E-03 | SMARCD1/KMT2B/ARID1A/ARID1B |
| BP | GO:0006661~phosphatidylinositol biosynthetic process | 9 | 9.31E-03 | INPPL1/PIK3CD/PI4KA/PIP5K1C/PIK3R5/INPP5D/PI4KB/MTMR6/FIG4 |
| BP | GO:0042795~snRNA transcription from RNA polymerase II promoter | 10 | 9.43E-03 | TAF11/INTS8/TAF6/POU2F2/GTF2A2/INTS1/INTS3/INTS10/GTF2B/POLR2A |
| BP | GO:0000723~telomere maintenance | 7 | 9.45E-03 | XRCC5/PTGES3/UPF1/SMG6/POLD1/CTC1/POT1 |
| BP | GO:0032496~response to lipopolysaccharide | 17 | 1.05E-02 | IRAK1/ALAD/ABR/BCR/PTGER4/LY96/PTPN22/TNFRSF14/NFKB2/IFNAR1/TNFRSF1A/NOTCH1/HDAC2/RELT/IL10RA/JUND/CHUK |
| BP | GO:0000398~mRNA splicing, via spliceosome | 21 | 1.08E-02 | RALY/PRPF4B/SRSF10/SNRPB2/CASC3/SF3A2/SF3A1/POLR2A/SRSF3/PAPOLA/DDX46/UPF3B/PLRG1/CD2BP2/PRPF8/SRRM2/LSM5/CWC22/CPSF3/SNRNP27/SNRPG |
| BP | GO:0006909~phagocytosis | 8 | 1.09E-02 | CSNK1A1/ABCA7/SCFD1/UNC13D/ANXA1/PIP5K1C/ITGB2/DNM2 |
| BP | GO:1901796~regulation of signal transduction by p53 class mediator | 14 | 1.14E-02 | TAF11/AKT1/TAF4/HDAC2/TAF6/STK11/MAPK14/L3MBTL1/TP53/RAD9A/TOPBP1/EHMT2/RAD17/NOC2L |
| BP | GO:0051726~regulation of cell cycle | 14 | 1.14E-02 | BCR/E2F5/COPS5/CCNI/L3MBTL1/BAP1/RB1/CCNG1/BIRC2/DOT1L/UBA3/TSC2/JUND/USP16 |
| BP | GO:0006468~protein phosphorylation | 36 | 1.19E-02 | FASTKD1/PRPF4B/STK11/STK10/MKNK2/EIF2A/RIOK2/AKT1/VRK1/PTK2B/RARA/CHUK/SIK3/CSNK1A1/IRAK1/BCR/LIMK1/PIK3CD/PIM1/MINK1/PKN1/CCNC/DGUOK/CDC25B/GAK/TYK2/GLYCTK/RPS6KA4/SCYL2/DYRK1B/GRK6/MAPK8/JAK3/MAP3K12/NEK6/MAP3K11 |
| BP | GO:0006103~2-oxoglutarate metabolic process | 5 | 1.19E-02 | MRPS36/D2HGDH/STAT5A/STAT5B/OGDH |
| BP | GO:0006105~succinate metabolic process | 4 | 1.24E-02 | STAT5A/SUCLG1/STAT5B/SUCLA2 |
| BP | GO:0006351~transcription, DNA-templated | 123 | 1.28E-02 | MMS19/XRCC5/RALY/STAT5A/MORF4L2/CNOT3/ZXDC/CNOT2/CNOT7/ZNF253/SFSWAP/SIN3B/MED29/SND1/ATF6B/RARA/DNAJC2/CCAR2/SAMD4B/ZNF43/RARG/ZNF592/RXRB/RXRA/MECP2/HDAC10/ARID1A/ARID1B/HIF1A/RFC1/PIAS4/FLII/DNTTIP2/SWT1/MCTS1/SMARCA4/ZNF131/CHCHD3/ZNRD1/MOV10/ATN1/PELP1/ZNF71/SUPT7L/BHLHE40/TRAF7/TCF3/ZBTB45/HDAC4/HDAC2/ZNF316/SMARCC2/KLF2/HDAC7/NCOR2/ZNF410/HDAC6/PPARD/ZNF486/E2F4/E2F5/ZNF675/RLIM/SLIRP/AES/ZNF738/PCGF6/MKL1/USP16/ELMSAN1/ZNF90/LDB1/ZNF92/TP53/ZNF142/PKN1/ZNF687/RB1/ZNF335/NOC2L/MXD4/CHMP1A/ZMIZ2/USP21/COMMD3/ZNF746/SRCAP/DPF2/KMT2D/SBNO2/KMT2B/ARHGAP35/PAXBP1/CIC/CCDC59/SFR1/NR1D1/TSC22D4/PRDM11/POU2F2/GATAD2A/GTF3C6/ZSCAN25/HBP1/CC2D1B/BAHD1/ZNF267/ZBTB7A/NACC1/BRD2/POLR3H/L3MBTL1/PHF10/DRG1/BIRC2/ATXN1/ATXN3/ZNF672/PHF19/YAF2/MAPK14/ZNF764/PBX2 |
| BP | GO:0034088~maintenance of mitotic sister chromatid cohesion | 3 | 1.48E-02 | NIPBL/MAU2/RB1 |
| BP | GO:0060534~trachea cartilage development | 3 | 1.48E-02 | RARG/RARA/SRF |
| BP | GO:1904294~positive regulation of ERAD pathway | 3 | 1.48E-02 | ATXN3/BAG6/TMEM259 |
| BP | GO:0000289~nuclear-transcribed mRNA poly(A) tail shortening | 6 | 1.51E-02 | PAIP1/CNOT3/CNOT2/MLH1/CNOT7/SAMD4B |
| BP | GO:0043928~exonucleolytic nuclear-transcribed mRNA catabolic process involved in deadenylation-dependent decay | 6 | 1.51E-02 | PATL1/EXOSC8/LSM5/EDC4/CNOT7/LSM1 |
| BP | GO:0033209~tumor necrosis factor-mediated signaling pathway | 13 | 1.84E-02 | PSMA2/PSMA1/TRAF2/TNFRSF1A/PSMA6/RELT/PTK2B/PSMD10/PSMA4/PSMA3/TNFRSF14/MAP3K14/BIRC2 |
| BP | GO:0031145~anaphase-promoting complex-dependent catabolic process | 10 | 1.99E-02 | PSMA2/CUL3/PSMA1/FZR1/ANAPC5/PSMA6/PSMD10/PSMA4/PSMA3/CDC16 |
| BP | GO:0006357~regulation of transcription from RNA polymerase II promoter | 34 | 2.01E-02 | HMGB1/CAMTA2/PPARD/STAT5A/ZNF131/STAT5B/CNOT2/MED24/ABCA2/NFKB2/BLZF1/HSF2/JUND/ATF6B/SMARCD1/CTDSP1/GLO1/USP16/ELMSAN1/ELP4/BRD2/TAF4/ANKRA2/SUB1/PKN1/ARID1A/RB1/MED15/MED16/MAPK14/SMARCC2/NFE2L1/SRCAP/SMARCA4 |
| BP | GO:0070932~histone H3 deacetylation | 5 | 2.07E-02 | HDAC4/HDAC2/HDAC10/HDAC7/HDAC6 |
| BP | GO:0006401~RNA catabolic process | 5 | 2.07E-02 | SND1/SKIV2L/SUPV3L1/RNASEH2B/XRN2 |
| BP | GO:0090090~negative regulation of canonical Wnt signaling pathway | 16 | 2.11E-02 | CSNK1A1/DVL2/DVL3/DVL1/RBX1/PSMA2/CUL3/PSMA1/NOTCH1/AES/PSMA6/SCYL2/PSMD10/MAPK14/PSMA4/PSMA3 |
| BP | GO:0042407~cristae formation | 4 | 2.11E-02 | LETM1/CHCHD3/OMA1/APOO |
| BP | GO:0001558~regulation of cell growth | 10 | 2.14E-02 | BLZF1/TAOK2/STK11/RASGRP2/CLSTN1/BAP1/EGLN2/RB1/CHPT1/EBAG9 |
| BP | GO:0045944~positive regulation of transcription from RNA polymerase II promoter | 66 | 2.16E-02 | AKNA/E2F4/E2F5/COPS5/ELF4/TBK1/STAT5B/MORF4L2/ZNF639/NFKB2/CNOT7/NFATC2IP/AKT1/NLRC5/HSF2/WWP2/GTF2A2/RARA/MKL1/USP16/TNIP1/CHUK/ARHGEF2/RARG/RXRB/LDB1/RXRA/TP53/MED12/CCNC/RB1/HIF1A/MTF2/ZMIZ2/NFE2L1/ZNF746/FHOD1/SMARCA4/HMGB1/KMT2D/SBNO2/CAMTA2/PAXBP1/ELK3/SRF/SUMO2/TNFRSF1A/NIPBL/PELP1/JUND/POU2F2/RUNX1/TCF3/TAF4/SUB1/CREBBP/DOT1L/HDAC4/NOTCH1/RPS6KA4/HDAC2/MAPK14/ARF4/KLF2/PBX2/SLC9A1 |
| BP | GO:0006974~cellular response to DNA damage stimulus | 19 | 2.17E-02 | MMS19/TAOK2/STK11/TP53/SMC6/RAD9A/TOPBP1/INTS3/BRAT1/CTC1/AKT1/CHD1L/NIPBL/UBA1/FANCF/USP16/MCTS1/RAD17/CCAR2 |
| BP | GO:0098609~cell-cell adhesion | 23 | 2.34E-02 | USP8/WASF2/RPL15/SNX2/PI4KA/EIF2A/TXNDC9/KLC2/CAPZB/LARP1/MPRIP/ATXN2L/SH3GLB2/PRDX6/RARS/SND1/TMOD3/PCMT1/NDRG1/EHD1/GOLGA3/PLEC/SH3GL1 |
| BP | GO:0010810~regulation of cell-substrate adhesion | 3 | 2.38E-02 | ATXN3/RAC2/MYADM |
| BP | GO:0006104~succinyl-CoA metabolic process | 3 | 2.38E-02 | SUCLG1/OGDH/SUCLA2 |
| BP | GO:0021766~hippocampus development | 8 | 2.41E-02 | PPP1R9B/PLXNA3/PAPD4/RARA/OGDH/UQCRQ/SRF/XRN2 |
| BP | GO:0006635~fatty acid beta-oxidation | 7 | 2.43E-02 | PPARD/PEX2/BDH2/HSD17B4/DECR1/HIBCH/HADHB |
| BP | GO:0010629~negative regulation of gene expression | 14 | 2.43E-02 | HAVCR2/TBK1/INPPL1/MAP2K2/PTPN22/RB1/CNOT7/RNASEH2B/MYADM/AKT1/ACE/AES/WWP2/CD46 |
| BP | GO:0071364~cellular response to epidermal growth factor stimulus | 6 | 2.56E-02 | AKT1/PPP1R9B/BECN1/STAT5B/CAD/PTPN12 |
| BP | GO:0051436~negative regulation of ubiquitin-protein ligase activity involved in mitotic cell cycle | 9 | 2.89E-02 | PSMA2/PSMA1/FZR1/ANAPC5/PSMA6/PSMD10/PSMA4/PSMA3/CDC16 |
| BP | GO:0036498~IRE1-mediated unfolded protein response | 8 | 3.11E-02 | ARFGAP1/SYVN1/CUL7/SHC1/CTDSP2/SEC62/DCTN1/SEC61A1 |
| BP | GO:0032743~positive regulation of interleukin-2 production | 4 | 3.23E-02 | TRAF2/ANXA1/RUNX1/SASH3 |
| BP | GO:0040029~regulation of gene expression, epigenetic | 4 | 3.23E-02 | HDAC4/KLF2/ZNF335/HDAC6 |
| BP | GO:0071044~histone mRNA catabolic process | 4 | 3.23E-02 | TUT1/UPF1/PAPD4/LSM1 |
| BP | GO:0006338~chromatin remodeling | 10 | 3.25E-02 | HDAC4/CHD1L/HDAC2/MORF4L2/SMARCC2/SMARCD1/ARID1A/RB1/ARID1B/SMARCA4 |
| BP | GO:0007088~regulation of mitotic nuclear division | 5 | 3.25E-02 | CUL7/FBXW5/CUL9/L3MBTL1/CDC16 |
| BP | GO:0071108~protein K48-linked deubiquitination | 5 | 3.25E-02 | USP19/ATXN3/USP8/USP5/BAP1 |
| BP | GO:0043433~negative regulation of sequence-specific DNA binding transcription factor activity | 8 | 3.37E-02 | HDAC4/HDAC2/WWP2/PIM1/BHLHE40/RB1/RLIM/FLNA |
| BP | GO:0016925~protein sumoylation | 12 | 3.86E-02 | NFATC2IP/SUMO2/NSMCE4A/PIAS4/RAE1/NUP210/TP53/SMC6/NUP54/TOP2B/NUPL2/HDAC7 |
| BP | GO:0007254~JNK cascade | 7 | 3.88E-02 | IRAK1/SH2D3C/PTGER4/MINK1/MAPK8/MAP3K12/MAP3K11 |
| BP | GO:0006122~mitochondrial electron transport, ubiquinol to cytochrome c | 4 | 3.89E-02 | MECP2/UQCRQ/PMPCB/UQCRHL |
| BP | GO:0051437~positive regulation of ubiquitin-protein ligase activity involved in regulation of mitotic cell cycle transition | 9 | 4.11E-02 | PSMA2/PSMA1/FZR1/ANAPC5/PSMA6/PSMD10/PSMA4/PSMA3/CDC16 |
| BP | GO:0006904~vesicle docking involved in exocytosis | 5 | 4.22E-02 | VPS18/SCFD1/VPS45/EXOC6/STXBP3 |
| BP | GO:0019827~stem cell population maintenance | 7 | 4.22E-02 | NIPBL/PHF19/TRIM8/MTF2/MED12/MED24/LSM1 |
| BP | GO:0032436~positive regulation of proteasomal ubiquitin-dependent protein catabolic process | 8 | 4.55E-02 | CSNK1A1/AKT1/SUMO2/GCLC/PSMD10/USP5/DNAJB2/DVL1 |
| BP | GO:0032481~positive regulation of type I interferon production | 7 | 4.59E-02 | XRCC5/IRAK1/POLR3H/TBK1/CREBBP/PTPN22/NFKB2 |
| BP | GO:0010592~positive regulation of lamellipodium assembly | 4 | 4.60E-02 | HDAC4/RAC2/WASF2/DNM2 |
| BP | GO:0032012~regulation of ARF protein signal transduction | 4 | 4.60E-02 | GBF1/CYTH4/PSD4/IQSEC1 |
| BP | GO:0022900~electron transport chain | 3 | 4.67E-02 | NDUFB3/ETFDH/CYB561 |
| BP | GO:0010870~positive regulation of receptor biosynthetic process | 3 | 4.67E-02 | HIF1A/HDAC2/HDAC6 |
| BP | GO:0060071~Wnt signaling pathway, planar cell polarity pathway | 10 | 4.69E-02 | PSMA2/DVL2/PSMA1/DVL3/PSMA6/PSMD10/PSMA4/PSMA3/MED12/DVL1 |
| BP | GO:0016192~vesicle-mediated transport | 14 | 5.00E-02 | VPS18/AP1G2/AP3S1/TSNARE1/SFT2D1/BLZF1/CHMP1A/FNBP1/COPG1/TSC2/AP3D1/MAPK8IP3/GGA1/GGA3 |
| CC | GO:0005654~nucleoplasm | 242 | 2.11E-20 | XRCC5/DYNC1LI1/KIAA0556/STAT5A/PPP2R5D/STAT5B/ZNF639/MED24/INTS1/PNISR/INTS3/CDC16/SLC26A11/CUL3/INTS8/SIN3B/CUL7/MED29/LSM5/RPP30/RARA/CCAR2/RARG/ANAPC5/RXRB/RXRA/MED12/RAD9A/ISG20L2/PNKP/DCAF7/MED15/PIAS4/RFC1/HUWE1/MTF2/MED16/RARS/FLII/MAPK8/NEK6/GNAI2/SRSF10/ZNRD1/VRK1/ATN1/POLM/TCF3/PCIF1/KANSL1/ZC3H18/TM2D1/CREBBP/SRSF3/NOTCH1/UPF3B/NSMCE4A/POMP/SNRNP27/NCOR2/SLC9A1/TAF1C/PPARD/COPS5/ELF4/TAF1D/COPS4/MLH1/NFKB2/RLIM/WBP4/BLZF1/BAG6/FANCE/DPP8/FANCF/TOP2B/GOLGA3/SYMPK/EXOSC8/TP53/MBD4/CCNC/DECR1/ECSIT/GTF2B/NOC2L/TAF11/USP21/THOC7/USP8/SKIV2L/FAM193B/MKNK2/POT1/POLR2A/SUMO2/NUMA1/NR1D1/CD2BP2/PTK2B/GATAD2A/MLLT1/AP3D1/CC2D1B/HBP1/POLR3H/UPF1/HIST1H2BF/SF3A2/SF3A1/DOT1L/PAPOLA/PHF19/RPS6KA4/YAF2/MAPK14/PSMD10/DGKZ/CWC22/TEX10/MMS19/PRPF4B/MAU2/MORF4L2/CNOT7/FAHD1/MIOS/SRRM2/ORC4/CTDSP1/CTDSP2/RBM10/DNAJC2/ORC3/AP5Z1/NEIL3/HDAC10/TOPBP1/ARID1A/ARID1B/NUPL2/CARF/PSMA2/PSMA1/HIF1A/PSMA6/PSMA4/PSMA3/RAD17/XRN2/EP400/SMARCA4/SNRPG/HMGB1/FZR1/SYVN1/STK11/STAM2/SNRPB2/ELK3/CCNG1/SRF/RIOK2/RBX1/DAZAP1/DDX47/NIPBL/CSE1L/PELP1/PPIL4/RUNX1/TAF4/TAF6/CASC3/EHMT2/INTS10/AK6/HDAC4/HDAC2/POLD1/SMARCC2/HDAC7/PTGES3/E2F4/E2F5/C9ORF78/BAP1/EDC4/CAD/LARP1/AKT1/PLRG1/TRIM8/RPS3A/GTF2A2/MKL1/TNIP1/CHUK/ELMSAN1/NOL6/ELP4/LIMK1/PKN1/RB1/MCM5/ZMIZ2/IPO5/CPSF3/PPP2R2A/DPF2/KMT2D/BTAF1/NDUFB4/NDUFB5/NDUFB6/KMT2B/EGLN2/HAT1/CCDC59/DIMT1/ZBTB8OS/CHD1L/PRPF8/POU2F2/GTF3C6/ERCC1/BAHD1/ZBTB7B/PM20D2/L3MBTL1/ANXA1/SMC6/CDC25B/SMC4/ATXN1/GPI/PPP1R9B/ATXN3/CABIN1/MIS18BP1/GGA1 |
| CC | GO:0005829~cytosol | 247 | 2.14E-12 | XRCC5/ALAD/STAT5A/PPP2R5D/STAT5B/RPL15/CDC16/OGDH/CUL3/CUL5/GBF1/CUL7/ATXN10/LSM5/LSM1/BCR/ANAPC5/SUCLG1/PIK3CD/MECP2/DCTN1/ST13/RAB18/HUWE1/FBXL8/RARS/MAPK8/NEK6/HSD17B11/ADSS/GNAI2/SRSF10/MYO9B/CAPZB/MOV10/VRK1/RAC2/RAP1GAP2/GIT1/DVL2/DVL3/SRP54/ABR/BECN1/MAP2K2/PAIP1/EPRS/PTPN12/PPA1/DVL1/NOTCH1/UPF3B/UBA1/UBA3/ARF4/POMP/HGS/RHEB/FUK/IDI1/COPS4/WASF2/NFKB2/RLIM/USP19/BAG6/NUBP1/PBXIP1/TOP2B/GOLGA3/FMNL1/EXOSC8/ARHGEF2/ARHGEF1/INPPL1/ZDHHC8/RAB4A/PI4KA/TP53/PI4KB/DGUOK/FLNA/ANKRD27/PAPD4/BNIP2/GNB1/FARSB/MAP3K14/MAP3K12/MIDN/USP8/PPP6R1/RPL37/KLC2/HPRT1/NUMA1/SORBS3/PTK2B/PPP2CB/EXOC6/PCMT1/MTMR6/PLEC/CSNK1A1/GDI1/POLR3H/UPF1/VTA1/BIRC2/GABARAP/NAE1/CAPN1/MAPK14/PSMD10/ARAF/MAT2B/DNM2/COG2/TBK1/CNOT3/CNOT2/UBQLN4/CNOT7/FAHD1/NLRC5/DNAJC2/RNF31/PCTP/CORO7/LYPLA1/NUPL2/PSMA2/PSMA1/HIF1A/COPG1/PSMA6/PSMA4/PSMA3/TESK1/YARS2/SNRPG/PACS1/ARFGAP1/TRAF2/FZR1/GCLC/SNX6/DIAPH1/STK11/STAM2/ARFRP1/SNX2/SNX3/RIOK2/RBX1/CSE1L/GMIP/EIF3H/GLO1/NDRG1/FGD3/SEC61A1/ARHGDIA/SMG6/MMADHC/SMG5/GALT/CASC3/MYL12A/TRIM62/GLYCTK/HDAC4/MAN2C1/SARM1/TSC2/RHOT1/RHOT2/RAP1A/DNAJB2/RAP1B/CRYZL1/HDAC6/PTGES3/METAP2/PTGES2/PREX1/NIPSNAP3A/PIP5K1C/EDC4/CAD/RGP1/AKT1/ARHGAP4/TRIM8/RPS3A/SHC1/MKL1/PLCB2/RHOF/CASP2/CHUK/IRAK1/PPP2R1A/CPTP/ANKRA2/PFKL/LIMK1/PKN1/MINK1/STXBP3/MID1IP1/GAK/TYK2/ATG4D/ARHGAP33/ATG4C/PRDX6/ZFYVE28/UGP2/PPP2R2A/VPS29/HAUS1/CYTH4/ARHGAP35/PAXBP1/SEC62/PATL1/ALDH1A2/TUBGCP6/RASGRP2/BDH2/PIK3R5/VPS35/SH2B1/INPP5D/WDTC1/SCOC/TPMT/CDC25B/SMC4/GPI/SCFD1/ATXN3/PSMG2/MAP1S/JAK3/ARAP1 |
| CC | GO:0016020~membrane | 178 | 1.18E-11 | AKNA/XRCC5/MMS19/DYNC1LI1/TMEM19/PLXNA3/CHERP/HM13/AP1G2/HBS1L/RPL15/CNOT2/INTS1/RCE1/CNOT7/TSNARE1/TAPBP/CUL3/SLC2A6/FAM49B/GBF1/WWP2/ATXN10/SND1/SLC4A2/DNAJC5/PHRF1/SLC12A9/BCR/UGCG/STRN4/MED12/PSD4/CORO7/MOGS/CHPT1/ALDH16A1/IL11RA/LPCAT4/DCTN1/ERGIC2/PNPLA6/PITPNM1/PNKP/PNPLA8/SCCPDH/CD37/IGSF8/UNC13D/MED15/HUWE1/MED16/RARS/ATP5C1/ARL8B/XRN2/MVP/SMARCA4/ORAI1/SYVN1/GNAI2/AGFG2/STK11/ARFRP1/SFXN3/SNX2/ITGB2/MYO9B/ABCA2/SNX4/CAPZB/ACE/DDX47/DDX46/CSE1L/RAC2/PELP1/EIF3H/SLC30A5/KLRF1/LFNG/IQSEC1/SEC61A1/GIT1/LAPTM4A/ABR/MYO1G/EPRS/NIPA2/AK6/VDAC1/SYNE3/ATP13A1/POLD1/TSC2/ARF4/GRK6/RHOT1/RHEB/RHOT2/RAP1A/RAP1B/TNK2/ALG12/NCOR2/LRRC8C/MLH1/EDC4/CAD/RGP1/LARP1/PIGK/BAG6/PEX2/GBAS/SLC25A3/ABHD13/PTDSS1/KDELR1/CASP2/GOLGA3/FMNL1/PPP2R1A/CLN3/PFKL/ANKRA2/LIMK1/PI4KA/LMBRD1/SLC9A3R1/FLNA/MCM5/GAK/ANKRD27/TYK2/SH2D3C/GNB1/PRDX6/ZDHHC13/IPO5/FARSB/LRMP/MAP3K12/FHOD1/MAP3K11/DEGS1/GALNT1/LMF2/KLC2/ITM2B/SEC62/SFT2D1/TUBGCP6/ATXN2L/PRPF8/NUP210/AP3D1/PIK3R5/SH2B1/HSD17B4/EHD1/NBEAL2/SELPLG/CSNK1A1/CNNM3/SLC12A4/ATP1A1/DRG1/BRAT1/CAPN1/GPI/HSDL2/DPM1/LRP8/JAK3/GGA1/RETSAT/COG2 |
| CC | GO:0005737~cytoplasm | 340 | 1.02E-09 | DYNC1LI1/KIAA0556/STAT5A/STAT5B/PTPN22/PNISR/CDC16/AQP3/SIN3B/CUL7/WWP2/RAE1/ATXN10/CUL9/RARA/LSM1/CCAR2/TBPL1/TXNL1/TTC7A/SUCLG1/STRN4/PIM1/UBR3/RAD9A/TXNDC9/ERGIC2/DCTN1/RIC8A/ST13/PITPNM1/DCAF7/HUWE1/MED15/PIAS4/RFC1/MTF2/RARS/FLII/ARL8B/MCTS1/NEK6/SH3GL1/HSD17B11/ADSS/STK11IP/GNAI2/SRSF10/CHCHD3/MYO9B/ARF5/PIN4/CAPZB/TIPRL/FAM65A/VRK1/RAC2/ATN1/DNAAF2/RAP1GAP2/TCF3/IQSEC1/DVL2/GIT1/SRP54/MSTO1/TAOK2/MAP2K2/PAIP2/PAIP1/CREBBP/EPRS/PTPN12/PPA1/SRSF3/UPF3B/UBA1/METTL10/POMP/HGS/MAP4/TNK2/FUK/PPP2R3C/SASH3/SLC9A1/DLEC1/COPS5/UBE2G1/RNPEPL1/TCOF1/EIF2A/NFKB2/RLIM/TTLL3/BLZF1/BAG6/NUBP1/HSF2/DPP8/MICAL1/USP16/TOP2B/SIK3/SYMPK/CLN3/ARHGEF2/EXOSC8/ARHGEF1/INPPL1/PI4KA/TP53/KLHDC1/ZNF687/PI4KB/DECR1/ECSIT/MBD1/FLNA/NOC2L/ANKRD27/EAPP/BNIP2/FARSB/USP21/THOC7/ZNF746/MAP3K14/MAP3K12/MAP3K11/USP8/FAM193B/MKNK2/PPP6R1/HPRT1/TRIM11/NUMA1/ATXN2L/NR1D1/CD2BP2/PTK2B/PPP2CB/MLLT1/PCMT1/UNK/MTMR6/KIF21B/PLEC/GDI1/BRD2/UPF1/NAT6/HIST1H2BF/BIRC2/BRAT1/NAE1/CAPN1/DRAM2/PAPOLA/RPS6KA4/LRP1/C21ORF2/YAF2/MAPK14/PSMD10/DGKZ/CWC22/CMTM3/TEX10/TOB2/DNM2/MMS19/CHERP/PLXNA1/TBK1/NIT2/CNOT2/UBQLN4/MBIP/SMNDC1/FAHD1/NLRC5/TBC1D15/MIOS/SND1/ORC4/DNAJC2/SAMD4B/AP5Z1/ACTN4/HDAC10/TOPBP1/LYPLA1/ARID1B/NUPL2/PSMA2/ANKRD13D/PSMA1/HIF1A/RELT/PSMA6/SGSM2/PSMA4/PSMA3/NFE2L1/YARS2/MAP7D1/MVP/ZFAND6/TRAF2/TMEM214/SNX6/STK11/STAM2/STK10/SNX2/SNX4/BEX4/SNX3/SRF/LLGL1/AZI2/DAZAP1/CSE1L/FBXW5/ST13P4/PELP1/PPIL4/HECTD3/NDRG1/GLO1/ST13P5/RUNX1/FGD3/ARHGDIA/B4GALT3/TAF4/SMG6/TAF6/SMG5/MMADHC/WDR6/MTRF1L/TRIM62/MPRIP/GLYCTK/TBC1D24/HDAC4/HDAC2/SARM1/POLD1/TSC2/RAP1A/HDAC7/HDAC6/PTGES3/METAP2/E2F5/LRRC8C/PREX1/C9ORF78/GABBR1/ESD/BAP1/EDC4/LARP1/NFATC2IP/ARHGAP4/AKT1/TRIM8/RPS3A/NARS2/KLHL22/KLHL24/MKL1/CASP2/TNIP1/CHUK/ELP4/IRAK1/CPTP/PFKL/ANKRA2/LIMK1/NDUFC2/PKN1/MINK1/SLC9A3R1/TYK2/EML3/SH2D3C/ATG4D/ARHGAP33/ATG4C/PRDX6/IPO5/COMMD3/FHOD1/UGP2/DPF2/VPS29/CPQ/GPSM3/HAUS1/PPP1R12C/HAT1/ARHGAP35/DNAH1/DIMT1/NSUN6/ALDH1A2/TUBGCP6/CHD1L/CCDC125/SH3GLB2/TSC22D4/PRDM11/POU2F2/BDH2/PIK3R5/ERCC1/TMC6/NACC1/GRSF1/ANXA1/DRG1/RGS14/CDC25B/SMC4/ATXN1/GPI/PPP1R9B/ATXN3/MAP1S/CABIN1/MAPK8IP3/TEP1/SH3BP1/GCA/ACTR10 |
| CC | GO:0005634~nucleus | 340 | 7.27E-08 | XRCC5/ALAD/KIAA0556/STAT5A/PPP2R5D/STAT5B/RPL15/ZNF639/MED24/PTPN22/INTS3/CDC16/ZNF253/NSRP1/AQP3/CUL3/SFSWAP/N4BP2L2/SIN3B/CUL7/WWP2/RAE1/LSM5/RPP30/RARA/FAM103A1/LSM1/CCAR2/CDCA4/TXNL1/ZNF43/RARG/ANAPC5/RXRB/CIZ1/RXRA/PIM1/MECP2/MED12/RAD9A/TXNDC9/ERGIC2/PNKP/HUWE1/MED15/PIAS4/RFC1/MTF2/MED16/MAPK8/NEK6/SRSF10/ZNF131/CHCHD3/DUSP11/PIN4/VRK1/ATN1/POLM/ZNF71/SUPV3L1/SUPT7L/TCF3/IQSEC1/DVL2/SRP54/TAOK2/KANSL1/TM2D1/BECN1/SUB1/MAP2K2/CREBBP/MPC2/ZBTB45/RPF2/VDAC3/VDAC1/TMEM115/NOTCH1/UPF3B/UBA1/UBA3/POMP/TNK2/KLF2/NCOR2/PPP2R3C/SASH3/PPARD/ZNF486/COPS5/ELF4/COPS4/TCOF1/MLH1/ZNF675/NFKB2/SLIRP/RLIM/BLZF1/BAG6/AES/PBXIP1/NUBP1/ZNF738/HSF2/SLC25A3/FANCE/USP16/TOP2B/GOLGA3/CLN3/EXOSC8/HIST1H1C/ZNF142/TP53/CCNC/ZNF687/MBD4/FAM76B/DGUOK/DECR1/ECSIT/GTF2B/MBD1/ZNF335/FLNA/KLHDC2/MXD4/NOC2L/EAPP/THOC7/ZNF746/SRCAP/SKIV2L/FAM193B/MKNK2/CIC/POT1/POLR2A/TRIM11/SUMO2/SORBS3/NUMA1/SFR1/NR1D1/CD2BP2/PTK2B/PPP2CB/REXO1/GATAD2A/MLLT1/ZSCAN25/CC2D1B/MLLT6/ZNF267/RAB2A/BRD2/UPF1/HIST1H2BF/PHF10/SF3A2/BIRC2/BRAT1/NAE1/DOT1L/PAPOLA/ZNF672/RPS6KA4/ANKRD49/YAF2/MAPK14/PSMD10/DPM1/DGKZ/MAT2B/ZNF764/PBX2/TOB2/DNM2/MMS19/AKNA/RALY/PLXNA3/PLXNA1/MAU2/MORF4L2/CNOT3/ZXDC/CNOT2/UBQLN4/MBIP/CNOT7/SMNDC1/NLRC5/MIOS/SND1/ATF6B/ORC4/CTDSP1/RBM10/DNAJC2/SAMD4B/AP5Z1/ZNF592/ACTN4/NEIL3/HDAC10/TOPBP1/ARID1A/NUPL2/RNASEH2B/CARF/PSMA2/PSMA1/SCCPDH/FOPNL/HIF1A/PSMA6/PSMA4/PSMA3/NFE2L1/DNTTIP2/SWT1/DCUN1D4/RAD17/XRN2/SMARCA4/MVP/HMGB1/CAMTA2/SNX6/STK11/BEX4/ELK3/SRF/ZNF330/DAZAP1/NIPBL/DDX46/CSE1L/PELP1/JUND/SLC30A5/HECTD3/NDRG1/BHLHE40/RUNX1/ARHGDIA/SMG6/TAF6/SMG5/MICU2/MUSTN1/EHMT2/CTC1/HDAC4/HDAC2/ZNF316/DYRK1B/POLD1/TSC2/DNAJB2/HDAC7/ZNF410/HDAC6/PTGES3/E2F4/PTGES2/E2F5/CLSTN1/NIPSNAP3A/EDC4/PIP5K1C/BAP1/CAD/NFATC2IP/AKT1/PLRG1/RPS3A/PCGF6/MKL1/CASP2/ELMSAN1/NOL6/IRAK1/PPP2R1A/CPTP/ANKRA2/ZNF90/ZNF92/LDB1/PKN1/C9ORF129/RB1/MID1IP1/SLC9A3R1/MCM5/TUT1/TYK2/SBF1/ZMIZ2/IPO5/COMMD3/UGP2/FHOD1/KMT2D/KMT2B/EGLN2/HAT1/ARHGAP35/PAXBP1/CCDC59/DIMT1/CHD1L/SH3GLB2/TSC22D4/PRPF8/PRDM11/POU2F2/PIK3R5/SH2B1/ERCC1/ZBTB7B/ZBTB7A/NACC1/WDTC1/L3MBTL1/ANXA1/SMC6/RGS14/SMC4/ATXN1/ATXN3/PSMG2/MAP1S/CABIN1/DENND4B/TIAF1 |
| CC | GO:0010008~endosome membrane | 28 | 4.09E-07 | VPS29/PLEKHM2/AP1G2/PLEKHM1/TBK1/SNX2/ABCA2/SNX3/ITM2B/AP3D1/VPS35/EHD1/TCIRG1/IRAK1/TM9SF2/CPTP/BECN1/LY96/VTA1/VPS45/SUN2/TMBIM1/FIG4/UBA1/SCYL2/RAB35/GGA1/GGA3 |
| CC | GO:0016607~nuclear speck | 28 | 2.14E-06 | SRSF10/PNISR/DUSP11/NSRP1/WBP4/SMNDC1/PATL1/ATXN2L/DDX46/PLRG1/CD2BP2/PRPF8/SRRM2/GATAD2A/CSNK1A1/SRP54/SMC6/FAM76B/CASC3/SF3A2/MBD1/SRSF3/TUT1/HIF1A/THOC7/CWC22/NEK6/EP400 |
| CC | GO:0005794~Golgi apparatus | 71 | 2.78E-05 | AP1G2/ELF4/AP3S1/SLC35A1/SLC26A11/ARHGAP4/CUL3/GBF1/CUL7/CD46/ATF6B/GOLGA3/CLN3/ARHGEF2/CPTP/INPPL1/VPS45/ZDHHC8/MINK1/CORO7/TOPBP1/ERGIC2/GAK/SCAP/TAF11/KIF1C/RAB11FIP5/EAPP/COPG1/SCYL2/ZDHHC13/IPO5/SRCAP/ARFGAP1/ARV1/ABCA7/GALNT1/USP8/TMEM214/CPQ/SLC39A13/ARFRP1/LMF1/ARF5/ITM2B/AP3D1/SLC30A5/BHLHE40/FGD3/SRGN/RAB2A/GDI1/TMC6/B4GALT3/LAPTM4A/LZTR1/MAP2K2/GALT/ATP1A1/TMBIM1/GABARAP/TMEM115/DRAM2/ITGA5/ARF4/TSC2/DENND4B/GGA1/CWC22/ARAP1/DNM2 |
| CC | GO:0000790~nuclear chromatin | 25 | 2.86E-05 | DPF2/PPARD/E2F4/HAT1/SRF/NR1D1/JUND/RARA/TCF3/CCAR2/DVL3/TAF4/RARG/RXRA/LDB1/CREBBP/TP53/ARID1A/EHMT2/MBD1/HDAC2/SMARCC2/RAD17/NCOR2/SMARCA4 |
| CC | GO:0005739~mitochondrion | 99 | 3.61E-05 | MRPS36/CMC2/FASTKD1/MRPL42/NIT2/OGDH/WDR81/HIBADH/FAHD1/TBC1D15/GBF1/SND1/DNAJC5/APOO/TXNL1/POLG/SUCLG1/MECP2/KIAA0141/LYPLA1/COX6C/SCCPDH/PNKP/SLC25A32/LETM1/RARS/ATP5C1/MRPL47/MAPK8/YARS2/MTFMT/PACS2/STK11/CHCHD3/SFXN3/ELK3/HADHB/GCSH/SUPV3L1/MRPS27/MMADHC/MAP2K2/BECN1/MICU2/C21ORF33/MPC1/MTRF1L/MPC2/ABCB7/VDAC3/VDAC1/GLYCTK/SARM1/UBA1/RHOT1/SLC9A1/D2HGDH/PTGES2/NIPSNAP3A/SLIRP/UQCRQ/AKT1/GBAS/NARS2/SLC25A3/TMEM102/SUCLA2/CASP2/DNAJC19/NOL6/PPP2R1A/CLN3/ZDHHC8/TP53/MRPL9/DGUOK/DECR1/ECSIT/ZMIZ2/DEGS1/NDUFB4/NDUFB5/NDUFB6/TNFRSF1A/BDH2/VPS35/HSD17B4/NDUFA4/TCIRG1/GRSF1/MRPL30/CAPN1/C21ORF2/HSDL2/MAPK14/RAB35/ARAF/MAT2B/HIBCH |
| CC | GO:0000139~Golgi membrane | 52 | 7.38E-05 | PTGES2/AP1G2/B3GALT4/CLSTN1/RGP1/SLC35A1/SLC35A4/TAPBP/CUL3/BLZF1/GBF1/KDELR1/GPR89A/GOLGA3/EBAG9/APOO/CLN3/VPS45/UGCG/CORO7/PI4KB/CHPT1/SCAP/PNPLA8/RAB11FIP5/COPG1/ZDHHC13/TMCO1/ABCA7/GALNT1/CYTH4/SLC35A5/PPP6R1/ITM2B/B3GNT9/TNFRSF1A/NUMA1/AP3D1/B4GALT6/SRGN/RAB2A/B4GALT3/SCOC/FIG4/GABARAP/TMEM115/NOTCH1/RAB35/RHEB/MAPK8IP3/COG2/DNM2 |
| CC | GO:0043234~protein complex | 40 | 7.51E-05 | ORAI1/SNX2/HAT1/SNX4/CAD/RGP1/KLC2/CIC/AKT1/BTBD1/TMEM102/TCF3/DNAJC19/HIGD1A/GDI1/ARHGEF2/BCR/ACTN4/BECN1/POLG/LDB1/WDR6/STRN4/ANXA1/TP53/PKN1/ATP1A1/BIRC2/SCAP/ST13/DOT1L/HDAC4/HDAC2/DCAF7/SMARCC2/SRCAP/SMARCA4/RBM17/DNM2/HDAC6 |
| CC | GO:0019773~proteasome core complex, alpha-subunit complex | 5 | 3.50E-04 | PSMA2/PSMA1/PSMA6/PSMA4/PSMA3 |
| CC | GO:0000932~cytoplasmic mRNA processing body | 13 | 4.11E-04 | UPF1/CNOT3/EDC4/CNOT2/CNOT7/PATL1/PSMA2/MOV10/PSMA6/BTBD1/PSMA4/LSM1/SAMD4B |
| CC | GO:0043231~intracellular membrane-bounded organelle | 47 | 4.58E-04 | VPS29/DPF2/HSD17B11/ABCA7/BTAF1/TAF1C/PLXNA3/SC5D/STK11IP/SNX6/PREX1/STAM2/AP3S1/HAT1/EDC4/ELK3/ITM2B/SLC26A11/TIPRL/PATL1/BAG6/TRIM8/CC2D1B/TRAF7/HSD17B4/RUNX1/CHUK/POLR3H/RAB4A/ATP1A1/SLC9A3R1/CHPT1/ERGIC2/GAK/ATXN1/RAB11FIP5/DRAM2/ATP13A1/BNIP2/PSMA4/ZDHHC13/CCDC53/IPO5/DPM1/HGS/GGA1/NEK6 |
| CC | GO:0016514~SWI/SNF complex | 6 | 5.70E-04 | SMARCC2/SMARCD1/ARID1A/RB1/ARID1B/SMARCA4 |
| CC | GO:0031901~early endosome membrane | 16 | 5.73E-04 | ABCA7/SNX6/STAM2/SNX2/ANXA1/SNX4/SNX3/FIG4/LLGL1/RAB11FIP5/PLEKHF2/ZFYVE28/HGS/EHD1/SNX13/SH3GL1 |
| CC | GO:0016363~nuclear matrix | 14 | 9.31E-04 | ATXN1/NUMA1/CHMP1A/ATXN3/DCAF7/PSMA6/PIAS4/ATN1/TP53/HAT1/TEP1/CAD/MBD1/NCOR2 |
| CC | GO:0005765~lysosomal membrane | 27 | 1.09E-03 | STK11IP/HM13/SLC38A9/PLEKHM1/SNX14/TMEM63A/ABCA2/SLC26A11/MIOS/AP3D1/DNAJC5/VPS35/TCIRG1/RAB2A/CLN3/VPS18/SLC12A4/ABCC10/TMBIM1/LMBRD1/ATP13A2/DRAM2/LRP1/UBA1/GNB1/RHEB/ARL8B |
| CC | GO:0000781~chromosome, telomeric region | 9 | 1.66E-03 | PTGES3/DOT1L/NSMCE4A/SMG6/HAT1/TEP1/SMC6/RAD17/POT1 |
| CC | GO:0071564~npBAF complex | 5 | 2.11E-03 | SMARCC2/SMARCD1/PHF10/ARID1A/SMARCA4 |
| CC | GO:0005789~endoplasmic reticulum membrane | 62 | 2.85E-03 | CYB5R4/SC5D/HM13/ORMDL1/LRRC8C/CLSTN1/UBQLN4/RCE1/ALG8/TAPBP/USP19/PIGK/PLOD3/ATF6B/PIGB/PIEZO1/PTDSS1/KDELR1/RAB21/APOO/SPTLC1/JKAMP/MOGS/PIGQ/PNPLA6/LPCAT4/ERGIC2/SCAP/PNPLA8/PITPNM1/COPG1/RAB18/LRMP/NFE2L1/DEGS1/ARV1/GALNT1/SYVN1/TMEM214/LMF2/LMF1/HSD17B12/TMEM259/SEC62/TMEM129/NUP210/SHISA5/RAB2A/TMC6/BECN1/P4HTM/NOTCH1/ATXN3/SCFD1/ATP13A1/ATP2A3/RAB35/DPM1/RHEB/ALG12/RETSAT/SLC9A1 |
| CC | GO:0030904~retromer complex | 6 | 3.75E-03 | VPS29/SNX6/SNX2/VPS35/SNX3/DCTN1 |
| CC | GO:0005743~mitochondrial inner membrane | 36 | 3.85E-03 | NDUFB3/MRPS36/NDUFB4/MRPL42/NDUFB5/NDUFB6/CHCHD3/UQCRQ/HADHB/FAHD1/ETFDH/SLC25A3/SLC25A40/OMA1/DNAJC19/HIGD1A/MRPS27/NDUFA4/MICU2/SUCLG1/NDUFC2/MPC1/MRPL9/TMEM126B/MRPS21/MPC2/ECSIT/ABCB7/MRPL30/VDAC1/COX6C/UQCRHL/SLC25A32/LETM1/ATP5C1/MRPL47 |
| CC | GO:0071565~nBAF complex | 5 | 3.94E-03 | SMARCC2/SMARCD1/ARID1A/ARID1B/SMARCA4 |
| CC | GO:0005622~intracellular | 87 | 5.71E-03 | ZNF486/ADCY7/PLEKHM1/TRPV2/HBS1L/WASF2/BAP1/ZNF675/TRIM46/TBC1D15/ZNF738/TRIM8/MFHAS1/TBC1D13/ATF6B/MOB3A/TMEM102/SHC1/RHOF/TNIP1/PLCB2/CHUK/ZNF43/ARHGEF2/BCR/ACTN4/PTGER4/ZNF90/RAB4A/ZNF92/PIK3CD/PI4KA/PI4KB/MAST3/SH2D3C/PNPLA8/PITPNM1/RAB18/GNB1/IPO5/MAPK8/ARL8B/ZNF746/MAP3K12/MAP3K11/TRAF2/SNX6/ARFRP1/SNX2/RAB40C/MYO9B/ARF5/RAC2/GMIP/RASGRP2/ZSCAN25/SH2B1/ZNF267/RAB2A/DVL2/GDI1/DVL3/SRP54/ABR/SMC6/RGS14/RABL2B/CDC25B/CAPN1/DVL1/RPS6KA4/ZNF316/TOM1L2/RAB35/MAPK14/ARF4/ARAF/RHOT1/RAP1A/RHEB/DGKZ/RHOT2/HGS/RAP1B/MAT2B/ZNF764/GGA1 |
| CC | GO:0005874~microtubule | 27 | 6.13E-03 | DYNC1LI1/HAUS1/DNAH1/KLC2/WDR81/TTLL3/ARHGAP4/TUBGCP6/PBXIP1/NDRG1/KIF21B/ARHGEF2/MAP2K2/MID1IP1/GABARAP/DCTN1/RGS14/DVL1/KIF1C/EML3/SARM1/MAP1S/MAP4/NEK6/DNM2/HDAC6/MAP3K11 |
| CC | GO:0031966~mitochondrial membrane | 12 | 6.46E-03 | SLC25A32/ATXN3/SYNJ2BP-COX16/NDUFB6/BECN1/PLSCR3/ANXA1/ETFDH/SFXN3/OGDH/OMA1/DNM2 |
| CC | GO:0005681~spliceosomal complex | 12 | 6.46E-03 | PRPF8/SNRPB2/LSM5/RHEB/SF3A2/CWC22/WBP4/SF3A1/CCAR2/SMNDC1/RBM17/SNRPG |
| CC | GO:0048471~perinuclear region of cytoplasm | 46 | 6.53E-03 | CYB5R4/GALNT1/PTGES2/CHERP/COPS5/PREX1/SLC39A13/UBQLN4/ZNF675/RAB40C/PTPN22/MYO9B/ARF5/SLIRP/ALDH1A2/CUL7/ATN1/PTK2B/ATXN10/HECTD3/NDRG1/RARA/RAP1GAP2/EHD1/ACTN4/MAP2K2/RAB4A/RNF207/ITGA3/CASC3/PI4KB/SLC9A3R1/FLNA/GABARAP/GAK/PNPLA8/BNIP2/MAP1S/SCYL2/TSC2/RAP1A/SRCAP/SLC9A1/MVP/DNM2/HDAC6 |
| CC | GO:0000922~spindle pole | 13 | 7.40E-03 | DYNC1LI1/HAUS1/TOPBP1/RGS14/DCTN1/CDC25B/TUBGCP6/NUMA1/POC1B/MAPK14/PPP2CB/LRMP/NEK6 |
| CC | GO:0005669~transcription factor TFIID complex | 7 | 7.72E-03 | TAF11/TAF4/TAF6/GTF2A2/TP53/GTF2B/ERCC1 |
| CC | GO:0031461~cullin-RING ubiquitin ligase complex | 4 | 8.01E-03 | CUL3/CUL5/CUL9/RBX1 |
| CC | GO:0071004~U2-type prespliceosome | 5 | 8.33E-03 | PRPF39/SF3A2/LUC7L/SF3A1/SNRPG |
| CC | GO:0016605~PML body | 12 | 8.78E-03 | PATL1/SUMO2/PIAS4/TRIM8/ELF4/MKNK2/TP53/SMC6/TOPBP1/RB1/LRCH4/RGS14 |
| CC | GO:0005730~nucleolus | 59 | 8.92E-03 | XRCC5/TAF1C/E2F5/TCOF1/MORF4L2/MBIP/PLRG1/RAE1/RPS3A/ORC4/SLC25A3/TOP2B/GOLGA3/NOL6/EXOSC8/CPTP/TP53/MED12/ISG20L2/GTF2B/FLNA/ERGIC2/NOC2L/CARF/TUT1/PNKP/RFC1/IPO5/DNTTIP2/XRN2/RAD17/SMARCA4/MIDN/SNRPB2/MKNK2/ZNRD1/PIN4/POLR2A/ZNF330/DIMT1/VRK1/DDX47/PELP1/SLC30A5/MLLT1/BHLHE40/SRP54/TAOK2/SMG6/SUB1/L3MBTL1/RPF2/AK6/RPF1/LRP1/UPF3B/MAP1S/TEX10/RCN2 |
| CC | GO:0031965~nuclear membrane | 21 | 9.60E-03 | TMC6/NDUFB4/FZR1/TMEM201/ELF4/SUN2/INTS1/FAM76B/NUPL2/KLHDC2/PLRG1/ATP2A3/NUP210/IPO5/SHISA5/NUP54/RAP1GAP2/DNAJC2/CMTM3/TEX10/RETSAT |
| CC | GO:0032580~Golgi cisterna membrane | 10 | 1.22E-02 | TMEM115/PITPNM1/GALNT1/SCFD1/B4GALT3/B4GALT6/RAB21/ARAP1/GOLGA3/GPR89A |
| CC | GO:0001891~phagocytic cup | 5 | 1.25E-02 | ABCA7/MYO1G/ANXA1/PIP5K1C/DNM2 |
| CC | GO:0035631~CD40 receptor complex | 4 | 1.46E-02 | TRAF2/BIRC2/CHUK/RNF31 |
| CC | GO:0000159~protein phosphatase type 2A complex | 5 | 1.51E-02 | PPP2R1A/PPP2R5D/PPP2CB/STRN4/PPP2R2A |
| CC | GO:0005686~U2 snRNP | 5 | 1.51E-02 | CCDC97/SNRPB2/SF3A2/SF3A1/SNRPG |
| CC | GO:0070062~extracellular exosome | 163 | 1.58E-02 | ALAD/ATP1B3/PLXNA1/NIT2/HBS1L/RPL15/SZT2/SLC26A11/CUL3/TBC1D15/N4BP2L2/FAM49B/WWP2/SND1/PLOD3/CD46/CTDSP1/DNAJC5/RAB21/ITFG1/TXNL1/SLC12A9/BCR/ACTN4/POLG/SUCLG1/LYPLA1/MOGS/ALDH16A1/ST13/PSMA2/PSMA1/PDDC1/CD37/IGSF8/RELT/RAB18/RFC1/HUWE1/PSMA6/RARS/PSMA4/PSMA3/ATP5C1/ARL8B/MVP/ADSS/GNAI2/STK11/STK10/TMEM63A/CHCHD3/SNX2/ITGB2/ARF5/SNX3/PIN4/CAPZB/HADHB/FAM65A/ACE/NIPBL/CSE1L/RAC2/ST13P4/EIF3H/GALNS/NDRG1/GLO1/ARHGDIA/HAVCR2/B4GALT3/SUB1/MYO1G/ITGA3/MYL12A/VDAC3/REEP5/PPA1/VDAC1/UBA1/ARF4/HGS/RHEB/RHOT2/MAP4/RAP1A/RAP1B/SLC9A1/PTGES3/ZNF486/UBE2G1/CLSTN1/COPS4/WASF2/ESD/CAD/MAN2B2/NUBP1/RPS3A/SLC25A3/SUCLA2/CTBS/RHOF/AMY2A/FMNL1/PPP2R1A/TM9SF2/PFKL/RAB4A/PI4KA/MINK1/STXBP3/DECR1/SLC9A3R1/MYADM/FLNA/TYK2/CHMP1A/GNB1/PRDX6/GINM1/UGP2/VPS29/NDUFB3/NDUFB4/ABHD8/CPQ/ITM2B/HPRT1/ZBTB8OS/TUBGCP6/NUMA1/PPP2CB/SHISA5/PCMT1/VPS35/BDH2/EHD1/PLEC/RAB2A/NDUFA4/TMC6/CES2/COX7A2/HIST1H2BF/VTA1/PM20D2/ANXA1/ATP1A1/TMBIM1/TPMT/CAPN1/GPI/TOM1L2/RAB35/MAPK14/JMJD8/ARSA/MAT2B/HIBCH/GCA/DNM2 |
| CC | GO:0009898~cytoplasmic side of plasma membrane | 7 | 1.62E-02 | TRAF2/MAP2K2/PTPN22/BIRC2/RAB21/CHUK/RNF31 |
| CC | GO:0005819~spindle | 13 | 1.62E-02 | MMS19/ARHGEF2/CDC16/RB1/PIN4/RGS14/DCTN1/AKT1/NUMA1/VRK1/MAP1S/MAP7D1/PPP2R3C |
| CC | GO:0005764~lysosome | 20 | 1.65E-02 | CLN3/VPS18/SLC38A9/SNX14/CPQ/USP5/ABCA2/ATP13A2/GABARAP/IFNAR1/CAPN1/ANKRD27/FNBP1/ACE/DRAM2/UNC13D/PRDX6/TSC2/ARSA/CTBS |
| CC | GO:0005770~late endosome | 13 | 1.72E-02 | VPS29/ANKRD27/ANKRD13D/CLN3/VPS18/UNC13D/SLC38A9/MAP2K2/SNX14/RAP1A/VPS35/ATP13A2/IFNAR1 |
| CC | GO:0005839~proteasome core complex | 5 | 1.79E-02 | PSMA2/PSMA1/PSMA6/PSMA4/PSMA3 |
| CC | GO:0030660~Golgi-associated vesicle membrane | 5 | 1.79E-02 | HM13/ZDHHC13/PI4KA/ITM2B/GPR89A |
| CC | GO:0016235~aggresome | 6 | 2.17E-02 | GIT1/POLD1/CABIN1/SEC62/XRN2/HDAC6 |
| CC | GO:0097422~tubular endosome | 3 | 2.20E-02 | ANKRD27/SNX6/VPS35 |
| CC | GO:0032039~integrator complex | 4 | 2.35E-02 | INTS8/INTS1/INTS3/INTS10 |
| CC | GO:0017119~Golgi transport complex | 4 | 2.35E-02 | TMEM115/SCFD1/GOLGA3/COG2 |
| CC | GO:0005875~microtubule associated complex | 6 | 2.44E-02 | DNAAF2/MAP4/LRP8/WDR81/GABARAP/HDAC6 |
| CC | GO:0016592~mediator complex | 6 | 2.73E-02 | MED15/MED29/MED16/MED12/MED24/CCNC |
| CC | GO:0000502~proteasome complex | 8 | 2.75E-02 | PSMA2/TXNL1/PSMA1/PSMG2/PSMA6/PSMD10/PSMA4/PSMA3 |
| CC | GO:0005768~endosome | 19 | 2.98E-02 | VPS29/TM9SF2/HMGB1/BECN1/RAB4A/ANXA1/PKN1/ATP1A1/SCOC/ABCA2/PI4KB/ACE/LRP1/ARSA/HGS/VPS35/TNK2/RAB21/DNM2 |
| CC | GO:0005913~cell-cell adherens junction | 25 | 3.04E-02 | USP8/WASF2/RPL15/SNX2/EIF2A/KLC2/CAPZB/LARP1/ATXN2L/SH3GLB2/SND1/NDRG1/PCMT1/EHD1/GOLGA3/PLEC/PI4KA/ANXA1/TXNDC9/FLNA/MPRIP/PRDX6/RARS/TMOD3/SH3GL1 |
| CC | GO:0000118~histone deacetylase complex | 6 | 3.05E-02 | HDAC4/HDAC10/NCOR2/HDAC7/ELMSAN1/HDAC6 |
| CC | GO:0090544~BAF-type complex | 3 | 3.19E-02 | SMARCC2/ARID1A/ARID1B |
| CC | GO:0005769~early endosome | 19 | 3.46E-02 | HAVCR2/VPS29/HMGB1/CLN3/VPS18/USP8/MAP2K2/WASF2/NIPA2/SNX3/ANKRD27/RAB11FIP5/CHMP1A/CCDC53/HGS/RAP1A/VPS35/EHD1/SNX13 |
| CC | GO:0045334~clathrin-coated endocytic vesicle | 4 | 3.48E-02 | DVL2/RAB35/LMBRD1/DNM2 |
| CC | GO:0071013~catalytic step 2 spliceosome | 10 | 3.73E-02 | RALY/PRPF4B/PLRG1/SRRM2/PRPF8/SNRPB2/SF3A2/CWC22/SF3A1/SNRPG |
| CC | GO:0030014~CCR4-NOT complex | 4 | 4.13E-02 | PATL1/CNOT3/CNOT2/CNOT7 |
| CC | GO:0032587~ruffle membrane | 9 | 4.86E-02 | ABCA7/PPP1R9B/ARHGEF2/ITGA5/DIAPH1/ARF4/RASGRP2/PIP5K1C/DNM2 |
| MF | GO:0005515~protein binding | 577 | 4.46E-20 | XRCC5/PLEKHM2/PPP2R5D/RPL15/ZNF639/MED24/INTS3/NSRP1/CUL3/INTS8/CUL5/CUL7/MED29/BTBD1/CUL9/LSM5/LSM1/TBPL1/CCAR2/CDCA4/RXRB/RXRA/VPS45/PIK3CD/MECP2/MED12/ISG20L2/ERGIC2/ST13/PITPNM1/UNC13D/DCAF7/MED15/MED16/SCYL2/FLII/MAPK8/ARL8B/MCTS1/SH3GL1/ADSS/ORAI1/GNAI2/HSD17B12/CHCHD3/ARF5/HADHB/ATN1/GCSH/TCF3/IQSEC1/DVL2/GIT1/DVL3/ZC3H18/PAIP2/MAP2K2/PAIP1/DVL1/VDAC1/SRSF3/TMEM115/SYNE3/FNBP1/UPF3B/UBA1/FAM214A/UBA3/ARF4/MAP4/HGS/TNK2/PPP2R3C/SLC9A1/TAF1C/ELF4/TAF1D/TCOF1/ZNF675/AP3S1/EIF2A/USP19/PBXIP1/NUBP1/HSF2/TMEM102/MICAL1/SIK3/FMNL1/CLN3/SYMPK/EXOSC8/ARHGEF2/TTC33/ARHGEF1/HIST1H1C/PI4KA/TP53/MRPL9/ZNF687/PI4KB/ZNF335/FLNA/MXD4/TAF11/MAST3/PAPD4/GNB1/BNIP2/USP21/THOC7/ZNF746/MKNK2/ITM2B/POT1/TRIM11/SFR1/SORBS3/NUMA1/PTK2B/PPP2CB/EXOC6/MTMR6/BRD2/UPF1/HIST1H2BF/VTA1/SF3A2/SF3A1/CAPN1/NAE1/DOT1L/LRP1/PHF19/RPS6KA4/PLEKHF2/C21ORF2/ARAF/PLSCR3/DPM1/DGKZ/LRP8/MAT2B/ZNF764/CWC22/CMTM3/DNM2/RALY/MMS19/CHERP/PRPF4B/AP1G2/MAU2/CNOT3/ZXDC/CNOT2/CNOT7/NLRC5/MFF/CHIC2/FAM49B/MFHAS1/SND1/ATF6B/SMARCD1/CTDSP1/CTDSP2/SPTLC1/ZNF592/ACTN4/PCTP/HDAC10/CORO7/TOPBP1/ARID1A/TMEM126B/ARID1B/HIF1A/COPG1/TESK1/NFE2L1/YARS2/DCUN1D4/DCUN1D5/EP400/XRN2/SMARCA4/ARFGAP1/HMGB1/TRAF2/CAMTA2/STK11/STK10/STAM2/SNRPB2/CCNG1/AZI2/ZNF330/DDX47/NIPBL/FBXW5/JUND/TRAF7/SEC61A1/ARHGDIA/TAF4/SMG6/TAF6/SMG5/MICU2/MYL12A/EHMT2/CYB561/CTC1/MPRIP/GLYCTK/HDAC4/HDAC2/SARM1/POLD1/DYRK1B/SMARCC2/TSC2/DNAJB2/HDAC7/HDAC6/ZNF410/PTGES3/E2F4/PTGES2/E2F5/PREX1/LRRC8C/CLSTN1/GABBR1/NIPSNAP3A/EDC4/BAP1/PIP5K1C/RGP1/TRIM8/PLRG1/PEX2/RPS3A/GTF2A2/DDA1/SUCLA2/CASP2/PPP2R1A/PTGER4/LIMK1/PKN1/STXBP3/MID1IP1/RB1/SLC9A3R1/GAK/KIF1C/SH2D3C/PRDX6/IPO5/ZFYVE28/CPSF3/UGP2/FHOD1/PPP2R2A/VPS29/DPF2/KMT2D/GPSM3/KMT2B/HAUS1/EGLN2/CHD1L/TSC22D4/PRPF8/SHISA5/GTF3C6/VPS35/SH2B1/INPP5D/EHD1/SELPLG/CNNM3/TMC6/VPS18/WDTC1/ANXA1/SMC6/DRG1/GDPD5/FIG4/CDC25B/SMC4/PPP1R9B/SCFD1/MIS18BP1/JAK3/GGA1/GGA3/GCA/RCN2/DYNC1LI1/TMEM19/HM13/STAT5A/STAT5B/PTPN22/LPAR2/CDC16/ERLEC1/SFSWAP/N4BP2L2/SIN3B/GBF1/WWP2/ATXN10/PLOD3/CD46/RPP30/RARA/FAM103A1/RAB21/APOO/RARG/BCR/POLG/CIZ1/STRN4/KIAA0141/PIM1/RAD9A/TXNDC9/DCTN1/RIC8A/PNKP/LETM1/CD37/RAB18/PIAS4/HUWE1/RFC1/FBXL8/RARS/LRCH4/NEK6/SRSF10/MYO9B/PIN4/TIPRL/VRK1/MOV10/DNAAF2/POLM/SUPV3L1/SUPT7L/NUP54/RAP1GAP2/SRP54/KANSL1/BECN1/SUB1/CREBBP/EPRS/RNF207/ATP13A2/PTPN12/RPF1/NOTCH1/NSMCE4A/GRK6/POMP/SNRNP27/KLF2/NCOR2/PPARD/COPS5/WASF2/COPS4/MLH1/NFKB2/SLIRP/WBP4/BLZF1/BAG6/AES/GBAS/FANCF/DNAJC19/GOLGA3/LY96/INPPL1/RAB4A/CCNC/MBD4/FAM76B/LENG8/ECSIT/GTF2B/MBD1/SCAP/NOC2L/ANKRD27/RAB11FIP5/CHMP1A/FARSB/MAP3K14/SRCAP/MAP3K12/MAP3K11/USP8/SLC39A13/USP5/PPP6R1/KLC2/CIC/HPRT1/POLR2A/SUMO2/ATXN2L/NR1D1/CD2BP2/IL10RA/GATAD2A/MLLT1/CC2D1B/HBP1/PCMT1/MLLT6/PLEC/RAB2A/CSNK1A1/GDI1/ATP1A1/BIRC2/BRAT1/GABARAP/PAPOLA/ANKRD49/YAF2/TOM1L2/PSMD10/MAPK14/RAB35/JMJD7/ARSA/LUC7L/PBX2/TOB2/TEX10/COG2/RBM17/PLXNA3/TBK1/MORF4L2/UBQLN4/MBIP/VIPR1/TAPBP/SMNDC1/ANKRD52/TBC1D15/MIOS/ORC4/RBM10/POC5/RNF31/ORC3/AP5Z1/TNFRSF14/NUPL2/PSMA2/PSMA1/IGSF8/PSMA6/PSMA4/PSMA3/RAD17/MVP/SNRPG/ZFAND6/PACS1/FZR1/SYVN1/SLC38A9/SNX6/DIAPH1/ARFRP1/SNX2/SNX4/ITGB2/ELK3/SNX3/SRF/LLGL1/RBX1/GMIP/CSE1L/PELP1/EIF3H/HECTD3/NDRG1/BHLHE40/RUNX1/SRGN/MRPS27/HAVCR2/MMADHC/WDR6/GALT/ITGA3/CASC3/INTS10/AK6/REEP5/TBC1D24/POC1B/ITGA5/RHOT1/RHOT2/RAP1A/KCTD15/RAP1B/HPS3/C9ORF78/ESD/LARP1/AKT1/ARHGAP4/PIGK/PCGF6/KLHL22/SHC1/MKL1/TNIP1/CHUK/ELP4/IRAK1/PFKL/ANKRA2/LDB1/MINK1/MCM5/IFNAR1/TUT1/TYK2/KLHL18/ARHGAP33/ZMIZ2/CCDC53/COMMD3/LMF2/PPP1R12C/HAT1/CCDC59/PATL1/TNFRSF1A/SH3GLB2/ERCC1/BAHD1/NDUFA4/ZBTB7B/ZBTB7A/L3MBTL1/SUN2/SCOC/RGS14/ATXN1/RPAP3/ATXN3/PSMG2/MAP1S/MAPK8IP3/SH3BP1/ARAP1/SH3BP2/ACTR10 |
| MF | GO:0031625~ubiquitin protein ligase binding | 38 | 1.99E-07 | XRCC5/TRAF2/HM13/UBE2G1/ZNF675/PTPN22/POLR2A/RBX1/CUL3/SUMO2/USP19/BLZF1/CUL5/BAG6/CUL7/BTBD1/CUL9/RNF31/ANKRA2/JKAMP/BECN1/SMG5/TP53/SMC6/TNFRSF14/RB1/CASC3/GABARAP/NAE1/GPI/ATXN3/HIF1A/PIAS4/PRDX6/DNAJB2/TNK2/ZNF746/HDAC6 |
| MF | GO:0008134~transcription factor binding | 35 | 3.37E-06 | HMGB1/CAMTA2/PPARD/E2F4/E2F5/PAXBP1/SRF/TRIM11/SORBS3/PELP1/WWP2/JUND/GTF2A2/RARA/RUNX1/TCF3/ELMSAN1/TAF4/ARHGEF2/CREBBP/TP53/PIM1/MECP2/DRG1/RB1/GTF2B/FLNA/DOT1L/HDAC4/HDAC2/HIF1A/PSMD10/PBX2/NCOR2/SMARCA4 |
| MF | GO:0003713~transcription coactivator activity | 32 | 3.70E-06 | MMS19/PPARD/COPS5/NFKB2/HSF2/JUND/GTF2A2/SMARCD1/RARA/SUPT7L/MKL1/USP16/TCF3/TBPL1/TAF4/SUB1/RXRB/RXRA/CREBBP/MED12/ARID1A/RB1/ARID1B/BIRC2/TAF11/MED16/YAF2/DYRK1B/SMARCC2/USP21/SRCAP/SMARCA4 |
| MF | GO:0042826~histone deacetylase binding | 17 | 5.61E-05 | WDTC1/CAMTA2/ANKRA2/SMG5/HDAC10/MECP2/PKN1/RAD9A/SRF/HDAC4/HIF1A/NIPBL/RARA/MAPK8/TOP2B/NCOR2/HDAC6 |
| MF | GO:0044822~poly(A) RNA binding | 88 | 6.65E-05 | XRCC5/RALY/DYNC1LI1/MRPL42/FASTKD1/CHERP/METAP2/PRPF4B/TRMT2A/TCOF1/RPL15/PNISR/SLIRP/NSRP1/SMNDC1/LARP1/RPS3A/SND1/SRRM2/RPP30/FAM103A1/RBM10/LSM1/DNAJC2/CCAR2/SAMD4B/NOL6/ARHGEF1/ACTN4/HIST1H1C/SUCLG1/MECP2/MRPL9/EIF1B/ISG20L2/NUPL2/FLNA/NOC2L/KIF1C/HUWE1/IPO5/ATP5C1/DNTTIP2/YARS2/XRN2/SNRPG/HMGB1/ZC3H4/SRSF10/DIAPH1/DUSP11/PIN4/CCDC59/HADHB/POLR2A/DAZAP1/DIMT1/PATL1/SUMO2/MOV10/DDX47/ATXN2L/DDX46/PELP1/EIF3H/PRPF8/PPIL4/SUPV3L1/UNK/PLEC/SRP54/UPF1/ZC3H18/SUB1/WDR6/GRSF1/MRPS21/CASC3/SF3A2/SF3A1/RPF2/RPF1/SRSF3/HDAC2/LRP1/UPF3B/UBA1/MAP4 |
| MF | GO:0005524~ATP binding | 109 | 1.21E-04 | XRCC5/DYNC1LI1/PRPF4B/ADCY7/TBK1/NLRC5/CUL9/ORC4/BCR/PIK3CD/PIM1/ABCC10/PNPLA8/PNKP/RFC1/SCYL2/RARS/TESK1/MAPK8/YARS2/EP400/NEK6/RAD17/SMARCA4/ABCA7/GCLC/STK11/STK10/ABCA2/MYO9B/RIOK2/VRK1/DDX47/FICD/MOV10/DDX46/SUPV3L1/RUNX1/TAOK2/MAP2K2/MYO1G/MYO1F/EPRS/ABCB7/ATP13A2/AK6/GLYCTK/ATP13A1/ATP2A3/UBA1/DYRK1B/UBA3/GRK6/TNK2/FUK/UBE2G1/MLH1/PIP5K1C/CAD/TTLL3/AKT1/NUBP1/NARS2/SUCLA2/TOP2B/SIK3/CHUK/IRAK1/PFKL/LIMK1/TP53/PI4KA/MINK1/PKN1/PI4KB/DGUOK/MCM5/GAK/TYK2/KIF1C/TUT1/MAST3/PAPD4/FARSB/MAP3K14/SRCAP/MAP3K12/MAP3K11/BTAF1/SKIV2L/MKNK2/DNAH1/ITM2B/CHD1L/PTK2B/EHD1/KIF21B/CSNK1A1/UPF1/SMC6/ATP1A1/SMC4/PAPOLA/RPS6KA4/MAPK14/ARAF/TEP1/DGKZ/JAK3 |
| MF | GO:0019899~enzyme binding | 33 | 4.48E-04 | MMS19/TRAF2/PREX1/CAD/AKT1/N4BP2L2/BLZF1/ATXN10/JUND/RARA/SLC4A2/TOP2B/CASP2/CCAR2/BCR/LDB1/RXRA/TP53/HDAC10/RAD9A/CASC3/DVL1/TUT1/NOTCH1/HDAC2/HIF1A/MAPK14/POLD1/TEP1/MAT2B/MAPK8/DNM2/HDAC6 |
| MF | GO:0003682~chromatin binding | 37 | 4.58E-04 | CAMTA2/STAT5B/BAP1/MLH1/NFKB2/CIC/SIN3B/NIPBL/PELP1/PRDM11/SMARCD1/TOP2B/DNAJC2/BAHD1/BRD2/UPF1/POLG/LDB1/CREBBP/L3MBTL1/TP53/MED12/MECP2/PKN1/MBD1/MCM5/NOC2L/HDAC4/HDAC2/POLD1/SMARCC2/PBX2/RAD17/EP400/NCOR2/HDAC7/TEX10 |
| MF | GO:0042809~vitamin D receptor binding | 6 | 6.67E-04 | TAF11/MED16/RXRA/MED12/MED24/TOB2 |
| MF | GO:0048365~Rac GTPase binding | 9 | 7.95E-04 | FMNL1/DVL2/ARHGAP4/DVL3/ARHGEF2/PKN1/FLNA/MAP3K11/DVL1 |
| MF | GO:0005096~GTPase activator activity | 28 | 1.09E-03 | ARFGAP1/AGFG2/PREX1/ARHGAP35/MYO9B/LLGL1/ARHGAP4/TBC1D15/GMIP/TBC1D13/RAP1GAP2/ARHGDIA/GIT1/GDI1/ARHGEF1/BCR/ABR/RGS14/RIC8A/ANKRD27/TBC1D24/ARHGAP33/SGSM2/ACAP3/BNIP2/TSC2/SH3BP1/ARAP1 |
| MF | GO:0019003~GDP binding | 10 | 1.51E-03 | RAB2A/DYNC1LI1/SRP54/RAB18/RAB4A/RAB35/RAB40C/RAP1B/ARL8B/RAB21 |
| MF | GO:0032041~NAD-dependent histone deacetylase activity (H3-K14 specific) | 5 | 2.39E-03 | HDAC4/HDAC2/HDAC10/HDAC7/HDAC6 |
| MF | GO:0003714~transcription corepressor activity | 21 | 3.72E-03 | LDB1/MECP2/ARHGAP35/ELK3/RLIM/MXD4/NOC2L/HDAC4/AES/NR1D1/ATN1/PBXIP1/PIAS4/YAF2/RARA/BHLHE40/RUNX1/NCOR2/TOB2/HDAC7/SMARCA4 |
| MF | GO:0003924~GTPase activity | 23 | 4.28E-03 | RAB2A/SRP54/MSTO1/GNAI2/RAB4A/HBS1L/ARFRP1/ARF5/RABL2B/RAB18/RAC2/GNB1/RAB35/ARF4/RHOT1/RAP1A/RHEB/RHOT2/RAP1B/ARL8B/RHOF/RAB21/DNM2 |
| MF | GO:0032403~protein complex binding | 21 | 4.39E-03 | NDUFA4/GIT1/TRAF2/STRN4/WASF2/ITGB2/SLC9A3R1/RBX1/SCAP/VDAC1/ST13/LRP1/PTK2B/GNB1/SLC25A3/RAP1A/RAP1B/RBM10/DNM2/PPP2R2A/COG2 |
| MF | GO:0004672~protein kinase activity | 31 | 5.59E-03 | FASTKD1/PRPF4B/STK11/TBK1/MKNK2/CAD/RIOK2/AKT1/VRK1/CHUK/SIK3/CSNK1A1/IRAK1/TAOK2/LIMK1/MAP2K2/PKN1/MINK1/GAK/TYK2/PPP1R9B/SCYL2/MAPK14/DYRK1B/ARAF/TESK1/TNK2/MAP3K14/NEK6/MAP3K12/MAP3K11 |
| MF | GO:0004674~protein serine/threonine kinase activity | 32 | 5.91E-03 | PRPF4B/TBK1/STK11/STK10/MKNK2/RIOK2/AKT1/VRK1/SIK3/CSNK1A1/IRAK1/BCR/TAOK2/LIMK1/MAP2K2/PIM1/MINK1/PKN1/CCNC/GAK/MAST3/RPS6KA4/MAPK14/DYRK1B/ARAF/TESK1/MAPK8/TNK2/MAP3K14/MAP3K12/NEK6/MAP3K11 |
| MF | GO:0042162~telomeric DNA binding | 6 | 6.45E-03 | XRCC5/UPF1/SMG6/SMG5/CTC1/POT1 |
| MF | GO:0005086~ARF guanyl-nucleotide exchange factor activity | 6 | 7.73E-03 | FNBP1/GBF1/ARF4/CYTH4/PSD4/IQSEC1 |
| MF | GO:0008565~protein transporter activity | 10 | 1.07E-02 | VPS29/AP1G2/RAB4A/IPO5/RAP1A/AP3S1/AP3D1/VPS35/SEC62/COG2 |
| MF | GO:0008270~zinc ion binding | 78 | 1.18E-02 | ALAD/PPARD/RNPEPL1/ZNF675/CAD/ZNF253/RLIM/WBP4/TRIM46/MAN2B2/TRIM8/PEX2/CUL9/PCGF6/MICAL1/RARA/RBM10/USP16/RNF31/PHRF1/ARHGEF2/RARG/ZNF90/LIMK1/RXRB/CIZ1/RXRA/ZNF92/NEIL3/ZDHHC8/TP53/UBR3/ZSWIM8/GTF2B/MBD1/CHMP1A/PIAS4/ZMIZ2/MTF2/ZDHHC13/COMMD3/PMPCB/DPF2/ZFAND6/TRAF2/KMT2D/SYVN1/USP5/KMT2B/ZNRD1/ZFAND1/RBX1/ZNF330/TRIM11/ACE/NR1D1/GATAD2A/SLC30A5/GLO1/TRAF7/MLLT6/UPF1/L3MBTL1/CREBBP/GALT/PHF10/RNF207/EHMT2/SF3A2/TRIM62/ATP13A2/BIRC2/HDAC4/MAN2C1/PHF19/YAF2/CRYZL1/HDAC6 |
| MF | GO:0098641~cadherin binding involved in cell-cell adhesion | 25 | 1.37E-02 | USP8/WASF2/RPL15/SNX2/EIF2A/KLC2/CAPZB/LARP1/ATXN2L/SH3GLB2/SND1/NDRG1/PCMT1/EHD1/GOLGA3/PLEC/PI4KA/ANXA1/TXNDC9/FLNA/MPRIP/PRDX6/RARS/TMOD3/SH3GL1 |
| MF | GO:0003723~RNA binding | 41 | 1.43E-02 | RALY/CHERP/SRSF10/SKIV2L/RPL15/RPL37/DUSP11/RLIM/CNOT7/DAZAP1/PATL1/SFSWAP/RAE1/RPS3A/PRPF8/LSM5/RPP30/SUPV3L1/HBP1/FAM103A1/SAMD4B/UPF1/PAIP1/GRSF1/THUMPD2/CASC3/SF3A1/RPF1/SRSF3/TUT1/ATXN1/PSMA1/PAPOLA/PSMA6/FARSB/THOC7/TEP1/CWC22/CPSF3/RBM17/SNRPG |
| MF | GO:0019903~protein phosphatase binding | 9 | 1.43E-02 | TRAF2/ANAPC5/MAPK14/STAT5B/TP53/PPP6R1/JAK3/SNX3/MVP |
| MF | GO:0001047~core promoter binding | 9 | 1.57E-02 | HDAC4/NOTCH1/HDAC2/KANSL1/RB1/RUNX1/GTF2B/POLR2A/HDAC6 |
| MF | GO:0070182~DNA polymerase binding | 4 | 1.60E-02 | HMGB1/SMG6/SMG5/SMARCA4 |
| MF | GO:0005154~epidermal growth factor receptor binding | 6 | 1.92E-02 | ITGA5/ARF4/SNX2/SHC1/SNX4/TNK2 |
| MF | GO:0004298~threonine-type endopeptidase activity | 5 | 2.01E-02 | PSMA2/PSMA1/PSMA6/PSMA4/PSMA3 |
| MF | GO:0004843~thiol-dependent ubiquitin-specific protease activity | 10 | 2.04E-02 | USP19/ATXN3/USP8/COPS5/USP5/USP21/BAP1/USP35/USP16/TNIP1 |
| MF | GO:0005351~sugar:proton symporter activity | 4 | 2.06E-02 | SLC2A6/SLC35A5/SLC35A1/SLC35A4 |
| MF | GO:0003708~retinoic acid receptor activity | 3 | 2.34E-02 | RARG/RXRA/RARA |
| MF | GO:0047485~protein N-terminus binding | 11 | 2.60E-02 | TAF11/SCFD1/NIPBL/ACTN4/MAU2/SRRM2/TP53/MECP2/BIRC2/NCOR2/SMARCA4 |
| MF | GO:0031593~polyubiquitin binding | 5 | 2.74E-02 | ZFAND6/BAG6/UBQLN4/DNAJB2/HDAC6 |
| MF | GO:0004386~helicase activity | 10 | 2.90E-02 | BTAF1/DDX47/MOV10/UPF1/SKIV2L/ANXA1/SUPV3L1/SRCAP/EP400/SMARCA4 |
| MF | GO:0019894~kinesin binding | 6 | 3.11E-02 | PLEKHM2/CLSTN1/MAPK8IP3/LRP8/KLC2/NEK6 |
| MF | GO:0008308~voltage-gated anion channel activity | 3 | 3.40E-02 | VDAC3/GPR89A/VDAC1 |
| MF | GO:0005525~GTP binding | 29 | 3.67E-02 | ADSS/DYNC1LI1/GNAI2/ARFRP1/HBS1L/RAB40C/ARHGAP35/ARF5/RAC2/MFHAS1/EHD1/RHOF/RAB21/RAB2A/SRP54/SUCLG1/RAB4A/DRG1/RABL2B/RAB18/RAB35/ARF4/RHOT1/RHEB/RAP1A/RHOT2/RAP1B/ARL8B/DNM2 |
| MF | GO:0019904~protein domain specific binding | 18 | 3.77E-02 | DVL2/PHRF1/E2F4/MECP2/MED12/ATP1A1/ITGA3/TRIM11/ST13/CHMP1A/ATN1/RFC1/POU2F2/HGS/RARA/BHLHE40/CASP2/ERCC1 |
| MF | GO:0016301~kinase activity | 20 | 4.00E-02 | CSNK1A1/GIT1/IRAK1/BCR/STK11/PIK3CD/PI4KA/PI4KB/DGUOK/GAK/AKT1/DGKZ/HGS/MAPK8IP3/PIK3R5/MAPK8/TNK2/FUK/MAP3K14/MAP3K11 |
| MF | GO:0017124~SH3 domain binding | 12 | 4.06E-02 | USP8/INPPL1/WASF2/PLSCR3/PTPN22/MICAL1/RAD9A/INPP5D/SH3BP1/PTPN12/DNM2/SH3BP2 |
| MF | GO:0003684~damaged DNA binding | 8 | 4.07E-02 | XRCC5/PNKP/HMGB1/POLD1/NEIL3/CREBBP/TP53/ERCC1 |
| MF | GO:0031491~nucleosome binding | 4 | 4.51E-02 | L3MBTL1/CABIN1/ARID1A/NOC2L |
| MF | GO:0031434~mitogen-activated protein kinase kinase binding | 4 | 4.51E-02 | ACE/TAOK2/MAPK8IP3/MAP3K11 |
| MF | GO:0051721~protein phosphatase 2A binding | 5 | 4.64E-02 | AKT1/SMG5/STRN4/TP53/PPP2R2A |
| MF | GO:0046966~thyroid hormone receptor binding | 5 | 4.64E-02 | TAF11/MED16/MED12/MED24/GTF2B |

Supplementary Table 4 GO enrichment and DEGs at 6 months after STEMI

| Category | Term | Count | PValue | Genes |
| --- | --- | --- | --- | --- |
| BP | GO:0007264/small GTPase mediated signal transduction | 24 | 5.15E-05 | RAB2A/GDI1/VAV3/RAB5B/RAB4A/RAB1A/RABL2B/SH2D3C/KRAS/RAB18/RAC2/MFHAS1/RAB35/SOS2/RAB11B/RHOT1/RHOBTB1/RAP1A/RHEB/RHOT2/RIT1/RAP1B/AGAP2/RAB21 |
| BP | GO:0015031/protein transport | 32 | 8.44E-05 | VPS29/ARFGAP1/RAB5B/SNX14/KIAA0196/UNC50/SNX4/CHMP2B/SFT2D1/GBF1/TIMM9/RANBP3/COX18/AGAP2/RAB21/PIK3R2/AP5Z1/RAB2A/GDI1/SCAMP2/GOLT1B/RAB4A/CORO7/SENP2/PITPNM1/SCFD1/PLEKHF2/AAAS/RAB18/RAB35/RAP1A/VAMP2 |
| BP | GO:0043161/proteasome-mediated ubiquitin-dependent protein catabolic process | 20 | 2.24E-04 | CSNK1A1/RAD23A/TP53/MTA1/SKP1/RLIM/UBE2B/PSMA1/PSMC6/ATXN3/RMND5A/FBXW5/PSMA6/UBXN2B/PPP2CB/PSMC1/KCTD17/HECTD3/PSME4/SPOPL |
| BP | GO:0016032/viral process | 24 | 8.80E-04 | ABCE1/PACS2/HTATIP2/CAMLG/AP1G2/RAD23A/TP53/ABI1/DAXX/HNRNPA1/GTF2B/SGTA/PDCL3/AAAS/HNRNPK/GBF1/RAE1/EIF4A2/GTF2A2/TSC2/IPO5/IL2RG/NUP54/SRCAP |
| BP | GO:0000389/mRNA 3'-splice site recognition | 4 | 1.62E-03 | ISY1/SLU7/SF3A2/SF3A1 |
| BP | GO:0030521/androgen receptor signaling pathway | 7 | 3.91E-03 | MED17/MED12/MED24/ARID1A/CDK7/DAXX/NRIP1 |
| BP | GO:0045944/positive regulation of transcription from RNA polymerase II promoter | 54 | 4.35E-03 | E2F4/COPS5/ZNF639/MED25/NFKB2/CXCR3/FLT3LG/MEN1/NLRC5/GATA3/GTF2A2/HSF4/MKL1/USP16/AGAP2/TNIP1/CHUK/RARG/LDB1/TP53/MED12/CCNC/CDK7/NRIP1/SENP2/MTF2/ZMIZ2/MED17/KMT2D/ELK1/PAXBP1/ELK3/SRF/SUMO2/PFN1/HNRNPK/NIPBL/PELP1/JUND/BCL9L/RUNX1/TCF3/PIK3R2/ASXL2/CCPG1/SMAD3/RAF1/SKI/RBMX/HDAC2/NELFA/KLF2/RERE/ATAD2B |
| BP | GO:0016567/protein ubiquitination | 25 | 4.40E-03 | SYVN1/MED24/RLIM/KLHL2/KBTBD2/FBXW5/CUL9/KLHL22/RABGEF1/FBXO4/KLHL24/DCAF15/WDTC1/DTX3/MED12/UBR3/SKP1/UBE2B/TRIM62/CISH/KLHL17/WDSUB1/MED17/FBXO33/NEURL3 |
| BP | GO:0006344/maintenance of chromatin silencing | 3 | 7.94E-03 | HDAC2/ARID1A/UBE2B |
| BP | GO:0007507/heart development | 15 | 8.86E-03 | POU6F1/PPARD/MED12/RAF1/CAD/ITGA3/MBD2/SRF/DVL1/SENP2/HEXIM1/RB1CC1/GATA3/TSC2/LOX |
| BP | GO:0006120/mitochondrial electron transport, NADH to ubiquinone | 7 | 9.46E-03 | NDUFA4/NDUFB3/NDUFB6/NDUFA8/DLD/NDUFAB1/NDUFA1 |
| BP | GO:0016569/covalent chromatin modification | 11 | 9.70E-03 | PHF19/BCORL1/MTF2/SMARCC2/SMARCD1/CABIN1/ARID1A/LRWD1/DNAJC2/DAXX/CBX6 |
| BP | GO:0019827/stem cell population maintenance | 7 | 1.04E-02 | NIPBL/PHF19/MTF2/MED17/MED12/MED24/LSM1 |
| BP | GO:0038061/NIK/NF-kappaB signaling | 8 | 1.13E-02 | PSMA1/PSMC6/PSMA6/PSMC1/NFKB2/PSME4/SKP1/CHUK |
| BP | GO:0016925/protein sumoylation | 11 | 1.22E-02 | SENP2/SUMO2/HNRNPK/AAAS/RAE1/TP53/MTA1/NUP54/TOP2B/SENP5/HDAC7 |
| BP | GO:0045892/negative regulation of transcription, DNA-templated | 30 | 1.26E-02 | BTAF1/PPARD/GCLC/ZNF552/SNX6/ZNF639/ELK3/RLIM/DAXX/MEN1/CIR1/NIPBL/HEXIM1/GATA3/GATAD2A/LOXL2/RUNX1/NACC1/LDB1/TP53/HDAC10/SIRT6/MBD2/UIMC1/ATXN1/HDAC2/YAF2/SMARCC2/MAP3K10/DNAJB6 |
| BP | GO:0010810/regulation of cell-substrate adhesion | 3 | 1.29E-02 | ATXN3/RAC2/MYADM |
| BP | GO:0006338/chromatin remodeling | 9 | 1.49E-02 | CHD1L/HDAC2/GATA3/SMARCC2/SMARCD1/ARID1A/ACTR8/DAXX/RERE |
| BP | GO:0006281/DNA repair | 17 | 1.54E-02 | PDS5B/RAD9A/BCCIP/MBD4/CEP164/TRRAP/UBE2B/BOD1L1/MEN1/PNKP/RPAIN/CHD1L/NABP2/PSME4/ACTR8/RTEL1/FANCC |
| BP | GO:1902474/positive regulation of protein localization to synapse | 3 | 1.89E-02 | CLSTN3/NLGN2/DVL1 |
| BP | GO:0006511/ubiquitin-dependent protein catabolic process | 14 | 1.92E-02 | PSMA1/ATXN3/PSMC6/USP8/PSMA6/CUL9/UBR3/FBXO4/UCHL3/SKP1/USP16/RLIM/UBE2B/USP15 |
| BP | GO:0000122/negative regulation of transcription from RNA polymerase II promoter | 39 | 2.00E-02 | PPARD/PPM1A/CHCHD3/MED25/NFKB2/CIC/RLIM/CBX6/MEN1/NIPBL/ATN1/HEXIM1/PEX2/GATA3/JUND/GATAD2A/CC2D1B/HSF4/TCF3/WDTC1/RARG/TP53/HDAC10/LMCD1/SMAD3/ARID1A/SKI/MBD2/EHMT2/NRIP1/SNAI3/PHF19/HDAC2/MTF2/SMARCC2/TXN/LRP8/HDAC7/RERE |
| BP | GO:0060071/Wnt signaling pathway, planar cell polarity pathway | 9 | 2.16E-02 | PFN1/PSMA1/DVL3/PSMC6/PSMA6/PSMC1/MED12/PSME4/DVL1 |
| BP | GO:1900034/regulation of cellular response to heat | 8 | 2.17E-02 | PTGES3/BAG5/AAAS/RAE1/BAG3/NUP54/DNAJC2/DNAJB6 |
| BP | GO:0000289/nuclear-transcribed mRNA poly(A) tail shortening | 5 | 2.18E-02 | TNKS1BP1/EIF4A2/CNOT3/MLH1/SAMD4B |
| BP | GO:0030033/microvillus assembly | 4 | 2.38E-02 | MINK1/RAP1A/RAP1B/FXYD5 |
| BP | GO:0098609/cell-cell adhesion | 18 | 2.63E-02 | ZC3H15/USP8/EPS15L1/ABI1/KLC2/RAB1A/CHMP2B/TNKS1BP1/PFN1/CDC42EP1/HNRNPK/SH3GLB2/BAG3/PAK4/RAB11B/NDRG1/EHD1/SH3GL1 |
| BP | GO:0048147/negative regulation of fibroblast proliferation | 5 | 2.72E-02 | PEX2/TP53/MED25/SKI/FBXO4 |
| BP | GO:0006367/transcription initiation from RNA polymerase II promoter | 12 | 2.74E-02 | PPARD/NRBP1/RARG/MED15/MED17/GTF2A2/MED12/MED25/MED24/CCNC/CDK7/GTF2B |
| BP | GO:0051170/nuclear import | 4 | 2.78E-02 | HTATIP2/SNRPE/HNRNPA1/SNRPG |
| BP | GO:0060324/face development | 4 | 2.78E-02 | ALDH1A2/RARG/RAF1/SRF |
| BP | GO:0006396/RNA processing | 9 | 2.86E-02 | ATXN1/CHERP/HNRNPK/LARP7/HNRNPD/GRSF1/MPHOSPH10/SF3A1/XRN2 |
| BP | GO:0006886/intracellular protein transport | 16 | 3.18E-02 | VPS29/AP1G2/SNX6/VPS41/TSNARE1/TBC1D15/ACD/SGSM2/TOM1L2/IPO5/HGS/SLU7/SAR1B/EHD1/GGA3/AP3B1 |
| BP | GO:0006892/post-Golgi vesicle-mediated transport | 4 | 3.22E-02 | SCFD1/SCAMP2/GBF1/VAMP2 |
| BP | GO:0006909/phagocytosis | 6 | 3.26E-02 | CSNK1A1/ABCA7/SCFD1/UNC13D/ADORA2A/PIP5K1C |
| BP | GO:0016926/protein desumoylation | 3 | 3.36E-02 | SENP2/FAM76B/SENP5 |
| BP | GO:0006351/transcription, DNA-templated | 89 | 3.49E-02 | POU6F1/PPARD/E2F4/CNOT3/RLIM/DAXX/CBX6/MEN1/ZNF738/ACTR8/HSF4/LOXL2/USP16/MKL1/DNAJC2/SAMD4B/ZNF43/KDM7A/RARG/LDB1/TP53/ZNF142/HDAC10/MTA1/ARID1A/ZBTB26/MBD2/NRIP1/PRDM8/ZNF341/ZMIZ2/ZNF692/TXN/COMMD3/VOPP1/SRCAP/KMT2D/ZNF552/SETD1B/ZNF76/KMT2B/CHCHD3/TRRAP/PAXBP1/CIC/COMMD8/DIDO1/ZNF653/CIR1/ATN1/PELP1/ZNF326/HEXIM1/RB1CC1/GTF3C6/GATAD2A/HNRNPD/ZSCAN25/BCL9L/HBP1/CC2D1B/VPS36/TCF3/ZNF267/ASXL2/NACC1/ZNF770/LMCD1/SMAD3/ZNF669/SKI/ZNF524/FOXP4/ZNF22/UIMC1/ATXN1/ATXN3/SNAI3/HDAC2/PHF19/YAF2/BCORL1/SMARCC2/RFX1/ZNF764/KLF2/HDAC7/RERE/ZNF410 |
| BP | GO:0000398/mRNA splicing, via spliceosome | 15 | 3.92E-02 | PRPF4B/LSM8/LSM6/SF3A2/HNRNPA1/SF3A1/RBMX/HNRNPK/DDX46/ISY1/DHX15/HNRNPD/SLU7/SNRPE/SNRPG |
| BP | GO:0006521/regulation of cellular amino acid metabolic process | 6 | 4.09E-02 | OAZ2/PSMA1/PSMC6/PSMA6/PSMC1/PSME4 |
| BP | GO:0048096/chromatin-mediated maintenance of transcription | 3 | 4.21E-02 | SMARCD1/KMT2B/ARID1A |
| BP | GO:0047497/mitochondrion transport along microtubule | 3 | 4.21E-02 | MAP1S/RHOT1/RHOT2 |
| BP | GO:0051569/regulation of histone H3-K4 methylation | 3 | 4.21E-02 | GATA3/KMT2B/EHMT2 |
| BP | GO:0002223/stimulatory C-type lectin receptor signaling pathway | 9 | 4.28E-02 | PSMA1/PSMC6/KRAS/PSMA6/PSMC1/RAF1/PSME4/SKP1/CHUK |
| BP | GO:0000723/telomere maintenance | 5 | 4.41E-02 | PTGES3/ACD/FBXO4/RTEL1/CTC1 |
| BP | GO:0070207/protein homotrimerization | 4 | 4.71E-02 | TRAF2/BRK1/SKI/HSF4 |
| BP | GO:0048813/dendrite morphogenesis | 5 | 4.81E-02 | MINK1/ABI1/HPRT1/RERE/DVL1 |
| BP | GO:0045893/positive regulation of transcription, DNA-templated | 28 | 4.81E-02 | PPARD/PPM1A/MED24/ELK1/ELK3/MAP3K2/GATA3/HNRNPD/USP16/RUNX1/DNAJC2/TCF3/DVL3/KDM7A/MAP2K2/TP53/MED12/SMAD3/ARID1A/CDK7/NRIP1/DVL1/IFNAR1/HDAC2/YAF2/MED17/SMARCC2/KLF2 |
| CC | GO:0005654/nucleoplasm | 176 | 8.42E-15 | FAM200B/NRBP1/PRPF4B/LSM8/PPP2R5D/LSM6/ZNF639/MED25/MED24/RBM7/SLC26A11/CBX6/FAHD1/GATA3/DNAJC2/AP5Z1/IBTK/RARG/SCAMP2/EFCAB13/MED12/HDAC10/MTA1/RAD9A/ARID1A/SKP1/PNKP/PSMA1/FAM222B/NABP2/AAAS/MED15/HUWE1/PSMA6/MTF2/MED17/DLD/SLU7/PIDD1/SNRPE/EP400/XRN2/SNRPG/SYVN1/SETD1B/ELK1/CEP164/BOP1/ELK3/TRRAP/CCNG1/SRF/PXN/HNRNPK/ACD/NIPBL/ATN1/PELP1/HEXIM1/ZNF326/ISY1/HNRNPD/RUNX1/TCF3/ASXL2/ZC3H18/MYO1C/MPHOSPH10/LMCD1/SMAD3/SKI/INTS12/EHMT2/INTS10/AK6/RBMX/HNRNPA1/UIMC1/TNKS1BP1/PSMC6/CCT4/HDAC2/PSMC1/SMARCC2/NELFA/RFX1/H3F3A/HDAC7/DNAJB6/PTGES3/TAF1C/PPARD/HTATIP2/E2F4/COPS5/MLH1/SHOC2/EDC4/CAD/NFKB2/SENP5/SKAP2/RLIM/DAXX/WBP4/BOD1L1/MEN1/RPS3A/MAPKAP1/GTF2A2/RANBP3/FANCE/HSF4/MKL1/LOXL2/TWISTNB/TOP2B/TNIP1/CHUK/RTEL1/FANCC/KDM7A/RRP36/TP53/MBD4/CCNC/CDK7/MBD2/GTF2B/UBE2B/NRIP1/SENP2/ZMIZ2/LARP7/IPO5/TXN/THOC7/PRKD3/MATR3/BTAF1/KMT2D/USP8/NDUFB6/FAM193B/KMT2B/PPM1A/HAT1/SUMO2/CHD1L/MAP3K2/GATAD2A/GTF3C6/MLLT1/BCL9L/CC2D1B/HBP1/C11ORF1/HIST1H2BC/PDS5B/HIST1H2BF/CENPP/PNO1/SIRT6/SF3A2/SF3A1/ZNF22/CDC25B/ATXN1/ATXN3/PHF19/BCORL1/YAF2/CABIN1/ZC3H11A/PPP1R13B/MIS18BP1 |
| CC | GO:0016020/membrane | 133 | 1.04E-09 | ADCY3/PLXNA3/CHERP/NRBP1/IL9R/AP1G2/RCE1/TSNARE1/TAPBP/RNF141/FAM49B/PRRT2/GBF1/IBTK/GOLT1B/MED12/CORO7/VPS41/IL11RA/LPCAT4/PNPLA8/SCCPDH/PNKP/PITPNM1/IGSF8/CD37/AAAS/UNC13D/HUWE1/MED15/MED17/ATP5C1/SLU7/VAMP2/XRN2/TPST1/ORAI1/MFNG/SYVN1/ME2/AGFG2/ADORA2A/SNX4/FXYD5/SEMA5A/PFN1/ACE/KRAS/DDX46/HNRNPK/RAC2/PELP1/RPL9/GIT1/CAMLG/ABCB8/SPHK2/MYO1C/SPTBN5/EVL/AK6/RBMX/HNRNPA1/PLEKHA3/PSMC6/ATP13A1/TSC2/PSMC1/RHOT1/GRK6/RHOT2/RHEB/RAP1A/RIT1/RAP1B/ALG12/DNAJB6/HTATIP2/RAB5B/CLSTN3/LRRC8C/NAP1L1/MLH1/EDC4/CAD/EPS15L1/RGP1/CANX/FLT3LG/BAG5/PEX2/SLC25A3/PTDSS1/LOXL2/AGAP2/CASP2/ANO6/AP3B1/ABCE1/ANKRA2/PIGO/SH2D3C/IPO5/LRMP/C16ORF58/MAP3K12/MATR3/DEGS1/GALNT1/CSF1/LMF2/SLC37A4/PPM1A/KLC2/ITM2B/SEC62/SFT2D1/PIK3R5/IL2RG/WDR11/HSD17B4/EHD1/ACSL4/ACSL3/SELPLG/CSNK1A1/AIMP1/NCDN/NLGN2/SGTA/SLCO1B1/LRP8/JAK3 |
| CC | GO:0005634/nucleus | 265 | 1.83E-09 | PPP2R5D/ZNF639/MED24/AQP3/RAE1/FAM103A1/LSM1/ZNF43/RARG/EFCAB13/PIM1/MED12/RAD9A/SKP1/PRDM8/PNKP/AAAS/MED15/HUWE1/MTF2/MED17/PIDD1/RAD23A/ZNF76/CHCHD3/UBA5/DUSP12/BOP1/DIDO1/PFN1/ACD/ATN1/HEXIM1/NGRN/BRIX1/TCF3/FRA10AC1/SRP54/BECN1/MAP2K2/SMAD3/SKI/ZNF524/HNRNPA1/TNKS1BP1/H3F3A/KLF2/RERE/ATAD2B/RANBP10/PPARD/HTATIP2/COPS5/TCOF1/VBP1/NAP1L1/MLH1/SHOC2/NFKB2/SENP5/RLIM/DAXX/BAG5/ZNF738/SLC25A3/FANCE/HSF4/ACTR8/USP16/TOP2B/TWISTNB/AGAP2/USP15/FANCC/ZC3H7A/RRP36/ANP32D/SLC25A6/ZNF142/TP53/MBD4/FAM76B/CCNC/MBD2/ZBTB26/GTF2B/EAPP/ZNF341/PDCL3/ZNF692/TXN/PFDN4/THOC7/SPOPL/SRCAP/ZNF552/FAM193B/BCCIP/ABI1/CIC/ZNF653/SUMO2/SPANXA1/CIR1/C19ORF66/PPP2CB/GATAD2A/DHX15/MLLT1/ZSCAN25/BCL9L/CC2D1B/WDR11/MLLT6/TPRKB/ZNF267/NEMF/RAB2A/HIST1H2BC/PDS5B/AIMP1/HIST1H2BF/ZNF770/ZNF669/SF3A2/SGTA/ZNF22/SNAI3/YAF2/MAT2B/ZNF764/TJP3/PLXNA3/PLXNA1/CNOT3/RBM7/CBX6/NLRC5/UBXN2B/GATA3/LOX/DNAJC2/SAMD4B/AP5Z1/HDAC10/MTA1/ARID1A/PSMA1/SCCPDH/FOPNL/NABP2/RPAIN/PSMA6/SLU7/SNRPE/XRN2/SNX6/SETD1B/ELK1/ELK3/TRRAP/SRF/COMMD8/CHMP2B/OAZ2/NIPBL/HNRNPK/DDX46/PELP1/ZNF326/RPL9/JUND/HNRNPD/HECTD3/NDRG1/SSX2IP/RUNX1/ARHGDIA/ABCB8/MICU2/LMCD1/SUGT1/EHMT2/FOXP4/RBMX/CTC1/UIMC1/PSMC6/HDAC2/TSC2/PSMC1/RFX1/DNAJB6/HDAC7/ZNF410/HINT3/PTGES3/POU6F1/NDUFAF6/E2F4/AGTPBP1/LYAR/NIPSNAP3A/PIP5K1C/EDC4/EPS15L1/CAD/MEN1/RMND5A/RPS3A/MAPKAP1/RANBP3/LRWD1/MKL1/LOXL2/CASP2/RTEL1/KDM7A/SGK2/ANKRA2/LDB1/CDK7/UBE2B/NRIP1/SBF1/ZMIZ2/LARP7/IPO5/COMMD3/UCHL3/PSME4/PRKD3/SPAST/MATR3/KMT2D/MOB1B/KMT2B/PPM1A/HAT1/PAXBP1/CHD1L/SH3GLB2/PIK3R5/RSL24D1/C11ORF1/VPS36/ZC3H12D/PIK3R2/NACC1/WDTC1/MORN2/NCDN/CENPP/PNO1/RAF1/SIRT6/RGS14/ATXN1/ATXN3/PSMG2/MAP1S/CABIN1/PPP1R13B/GGNBP2/TIAF1 |
| CC | GO:0005829/cytosol | 178 | 6.90E-09 | NRBP1/PPP2R5D/LSM6/CNOT3/OGDH/RAB1A/KLHL2/FAHD1/NLRC5/GBF1/BRPF3/UBXN2B/LSM1/SAR1B/DNAJC2/NANP/PIK3CD/CORO7/VPS41/LYPLA1/SKP1/PSMA1/RAB18/HUWE1/PSMA6/RIOK3/FBXL8/PIDD1/VAMP2/SNRPE/PCCA/NRBP2/SNRPG/ARFGAP1/HSD17B11/TRAF2/ADSS/GCLC/MYL5/SNX6/UBA5/DUSP12/CEP164/PXN/CHMP2B/OAZ2/PFN1/KRAS/RAC2/RPL9/HNRNPD/RHOBTB1/FBXO4/NDRG1/AMD1/ARHGDIA/EIF3M/GIT1/DVL3/SRP54/VAV3/SPTBN5/MYO1C/SPHK2/BECN1/MAP2K2/MMADHC/SMAD3/EVL/MYL12A/SUGT1/TRIM62/PTPN12/DVL1/TNKS1BP1/URM1/PSMC6/MAN2C1/CCT4/TSC2/PSMC1/RHOT1/HGS/RHEB/RHOT2/RAP1A/RAP1B/FUK/IDI1/DNAJB6/RANBP10/PTGES3/METAP2/AGTPBP1/NIPSNAP3A/EDC4/PIP5K1C/CAD/NFKB2/RGP1/SKAP2/RLIM/DAXX/BAG5/MEN1/RPS3A/MAPKAP1/BAG3/RANBP3/ABHD10/MKL1/TOP2B/CASP2/CHUK/EFR3B/FANCC/ARHGEF1/SGK2/ANKRA2/RAB4A/TP53/MINK1/STXBP3/PAPD4/BNIP2/EIF4A2/TXN/PFDN4/PSME4/MAP3K12/VPS29/MOB1B/USP8/TNFRSF25/KIAA0196/HAUS1/PPM1A/PPP6R1/ABI1/PAXBP1/KLC2/HPRT1/SEC62/ALDH1A2/MAP3K2/RB1CC1/PPP2CB/SOS2/PIK3R5/BRK1/INPP5E/LYPLAL1/RPIA/TPRKB/VPS36/PIK3R2/CSNK1A1/GDI1/WDTC1/PDS5B/AIMP1/NCDN/CENPP/PNO1/RAF1/SCOC/AMBRA1/CISH/SGTA/CDC25B/SCFD1/ATXN3/PSMG2/MAP1S/ARAF/MAT2B/JAK3/FAM126A |
| CC | GO:0000790/nuclear chromatin | 21 | 2.11E-05 | DVL3/PPARD/RARG/E2F4/LDB1/TP53/HAT1/SMAD3/ARID1A/EHMT2/MBD2/SRF/UBE2B/NRIP1/MEN1/HNRNPK/HDAC2/GATA3/JUND/SMARCC2/TCF3 |
| CC | GO:0005737/cytoplasm | 235 | 3.06E-05 | ADCY3/KIFC2/FAM200B/AQP3/RAE1/CUL9/LSM1/ADAM8/IBTK/EFCAB13/PIM1/UBR3/RAD9A/SKP1/PITPNM1/AAAS/MED15/HUWE1/MTF2/PIDD1/SH3GL1/HSD17B11/ADSS/CHCHD3/UBA5/DUSP12/BOP1/DIDO1/PXN/PFN1/KRAS/ACD/CDC42EP1/RAC2/ATN1/HEXIM1/FBXO4/TCF3/GIT1/SRP54/ZC3H15/MAP2K2/PAIP2/SMAD3/SKI/HNRNPA1/PPA2/PTPN12/TNKS1BP1/CCT4/HGS/MAP4/FUK/DLEC1/HTATIP2/COPS5/TCOF1/VBP1/SHOC2/NFKB2/RLIM/DAXX/BAG3/TOP2B/USP16/USP15/FBXL16/FANCC/ABCE1/ARHGEF1/TP53/MBD2/EAPP/PDCL3/BNIP2/PFDN4/TXN/MAP3K10/THOC7/SPOPL/MAP3K12/USP8/FAM193B/PPP6R1/ABI1/HPRT1/SPANXA1/CIR1/RB1CC1/C19ORF66/PPP2CB/DHX15/MLLT1/WDR11/LYPLAL1/UNK/ACSL4/TPRKB/KIF21B/GDI1/PPP1R18/HIST1H2BC/NAT6/AIMP1/HIST1H2BF/AMBRA1/CISH/SGTA/BCORL1/YAF2/HSP90AB3P/ZMYND19/FAM126A/CHERP/PLXNA1/KLHL2/FAHD1/NLRC5/TBC1D15/DNAJC2/SAMD4B/AP5Z1/STMN3/HDAC10/MTA1/LYPLA1/ANKRD13D/PSMA1/RPAIN/NABP2/SGSM2/PSMA6/SLU7/MAP7D1/NRBP2/ZFAND6/TRAF2/TMEM214/SNX6/SETD1B/SNX4/SRF/COMMD8/CHMP2B/OAZ2/HNRNPK/FBXW5/PELP1/RPL9/HECTD3/NDRG1/RUNX1/ARHGDIA/PRSS53/B4GALT3/MYO1C/SPTBN5/MMADHC/LMCD1/EVL/TRIM62/HDAC2/LCE1E/PSMC1/TSC2/NELFA/KCTD17/RAP1A/TP53I13/HDAC7/HINT3/PTGES3/HNRNPA1L2/NDUFAF6/METAP2/AGTPBP1/LRRC8C/EDC4/CXCR3/SKAP2/MEN1/RMND5A/RPS3A/MAPKAP1/RANBP6/KLHL22/KLHL24/LRWD1/MKL1/TNIP1/CASP2/CHUK/ANKRA2/MINK1/CDK7/UBE2B/SH2D3C/LARP7/IPO5/AAMP/COMMD3/UCHL3/PRKD3/SPAST/VPS29/MOB1B/CPQ/HAUS1/GPSM3/HAT1/DNAH1/ALDH1A2/CHD1L/WDPCP/SH3GLB2/CCDC124/MAP3K2/PIK3R5/ZC3H12D/NACC1/TMC8/DTX3/GRSF1/RAF1/SIRT6/RGS14/CDC25B/ATXN1/ATXN3/MAP1S/CABIN1/PPP1R13B/SH3BP1/GGNBP2/ACTR10 |
| CC | GO:0005681/spliceosomal complex | 13 | 1.32E-04 | HNRNPA1L2/LSM8/ZNF326/LSM6/SLU7/RHEB/SF3A2/WBP4/HNRNPA1/SNRPE/SF3A1/RBM17/SNRPG |
| CC | GO:0005622/intracellular | 71 | 7.83E-04 | ADCY3/RAB5B/TRPV2/RAB1A/TBC1D15/ZNF738/MFHAS1/MOB3A/RANBP3/GUCY1A3/SAR1B/AGAP2/TNIP1/ANO6/ANO9/CHUK/ZNF43/SGK2/RAB4A/PIK3CD/VPS41/SH2D3C/PNPLA8/PITPNM1/RAB18/IPO5/MAP3K10/UCHL3/PRKD3/MAP3K12/TRAF2/GPN3/ZNF552/SNX6/MAP4K2/ABI1/CHMP2B/GFM2/KRAS/RAC2/SOS2/RAB11B/ZSCAN25/RHOBTB1/IL2RG/ZNF267/RAB2A/GDI1/DVL3/SRP54/VAV3/RAF1/SMAD3/ZNF669/RGS14/RABL2B/CDC25B/DVL1/TOM1L2/HEATR3/RAB35/ARAF/RHOT1/RHEB/RHOT2/HGS/RAP1A/RIT1/MAT2B/RAP1B/ZNF764 |
| CC | GO:0043231/intracellular membrane-bounded organelle | 35 | 1.90E-03 | VPS29/HSD17B11/ABCA7/TAF1C/BTAF1/PLXNA3/ME2/SNX6/SLC37A4/VBP1/HAT1/EDC4/UBA5/ELK3/ITM2B/SENP5/SLC26A11/ZNF326/CC2D1B/RPIA/HSD17B4/RUNX1/CHUK/SCAMP2/RAB4A/MTA1/CTSS/ATXN1/ATP13A1/BNIP2/IPO5/RFX1/SLU7/HGS/VAMP2 |
| CC | GO:0005811/lipid particle | 9 | 2.39E-03 | HSD17B11/SCCPDH/PITPNM1/GBF1/RAP1B/ACSL4/EHD1/ACSL3/SPAST |
| CC | GO:0030532/small nuclear ribonucleoprotein complex | 5 | 2.68E-03 | LSM6/SLU7/SF3A2/SNRPE/SNRPG |
| CC | GO:0043234/protein complex | 27 | 3.94E-03 | ORAI1/GPN3/CLSTN3/HAT1/CAD/SNX4/RGP1/KLC2/CIC/CANX/MEN1/SSX2IP/TCF3/HIGD1A/GDI1/BECN1/LDB1/TP53/SKI/MBD2/SUGT1/SCAP/HDAC2/SMARCC2/H3F3A/SRCAP/RBM17 |
| CC | GO:0005686/U2 snRNP | 5 | 5.01E-03 | CCDC97/SF3A2/SNRPE/SF3A1/SNRPG |
| CC | GO:0000502/proteasome complex | 8 | 5.46E-03 | PSMA1/PSMC6/PSMG2/PSMA6/RAD23A/PSMC1/SHFM1/PSME4 |
| CC | GO:0071013/catalytic step 2 spliceosome | 10 | 5.68E-03 | HNRNPK/PRPF4B/ISY1/SLU7/SF3A2/RBMX/SNRPE/HNRNPA1/SF3A1/SNRPG |
| CC | GO:0005794/Golgi apparatus | 46 | 7.82E-03 | TPST1/ARFGAP1/ADCY3/ABCA7/GALNT1/USP8/TMEM214/AP1G2/CPQ/SLC39A13/LMF1/FGFRL1/TRRAP/ITM2B/SLC26A11/RAB1A/CDC42EP1/GBF1/UBXN2B/CD46/MAPKAP1/PAK4/SAR1B/ACSL3/AP3B1/RAB2A/GDI1/B4GALT3/TMC8/SCAMP2/AIMP1/STMN3/MAP2K2/MINK1/RAF1/CORO7/SCAP/SLC35C2/PLEKHA3/EAPP/LARP7/TMEM43/IPO5/TSC2/PIDD1/SRCAP |
| CC | GO:0016363/nuclear matrix | 10 | 8.00E-03 | ATXN1/MEN1/ATXN3/PSMA6/ATN1/ZNF326/TP53/HAT1/CAD/MATR3 |
| CC | GO:0000118/histone deacetylase complex | 6 | 8.74E-03 | CIR1/HDAC10/MBD2/RERE/HDAC7/NRIP1 |
| CC | GO:0005739/mitochondrion | 65 | 9.47E-03 | AGTPBP1/NIPSNAP3A/OGDH/HIBADH/FAHD1/BAG5/TBC1D15/GBF1/TIMM9/SLC25A3/ABHD10/ATP5L/AGAP2/CASP2/ABCE1/SLC25A6/TP53/LYPLA1/CDK7/SCCPDH/PNKP/ZMIZ2/DLD/TXN/PFDN4/ATP5C1/MRPL47/DEGS1/PACS2/NDUFB6/ME2/COX7B/CHCHD3/ELK1/ELK3/HADHB/CCDC58/CHMP2B/GFM2/KRAS/RAB11B/HSD17B4/LIPT1/ETFA/NDUFA4/ABCB8/NDUFA8/MMADHC/MAP2K2/BECN1/MICU2/GRSF1/AMBRA1/MRPL30/AFG3L2/NDUFA1/APOPT1/MRPL22/RAB35/MTFP1/ARAF/RHOT1/YJEFN3/MAT2B/PPP1R13B |
| CC | GO:0005789/endoplasmic reticulum membrane | 44 | 1.80E-02 | CYB5R4/GALNT1/SYVN1/TMEM214/CLSTN3/LRRC8C/LMF2/ORMDL1/HSD17B12/LMF1/SLC37A4/RCE1/SEC62/CANX/RAB1A/TAPBP/SERINC1/SHISA5/PTDSS1/SAR1B/ACSL4/ACSL3/RAB21/RAB2A/TMC8/SEC11C/PIGX/BECN1/LPCAT4/PIGO/SCAP/PITPNM1/PNPLA8/ATXN3/SCFD1/ATP13A1/RAB18/ATP2A3/RAB35/RHEB/LRMP/ALG12/SPAST/DEGS1 |
| CC | GO:0005730/nucleolus | 43 | 2.53E-02 | TAF1C/LSM6/LYAR/TCOF1/BOP1/SENP5/DAXX/RAE1/PELP1/RPS3A/GATA3/RPL9/BRIX1/SLC25A3/DHX15/MLLT1/BCL9L/RSL24D1/TOP2B/TWISTNB/AGAP2/KDM7A/SRP54/ZC3H15/ABCB8/MYO1C/RRP36/CENPP/PNO1/TP53/MPHOSPH10/MED12/SIRT6/AK6/GTF2B/RPF1/NRIP1/PNKP/RPAIN/MAP1S/IPO5/XRN2/RCN2 |
| CC | GO:0042470/melanosome | 9 | 2.86E-02 | RAB2A/CCT4/RAB5B/SGSM2/RAB35/TRPV2/NAP1L1/CANX/RAB1A |
| CC | GO:0005743/mitochondrial inner membrane | 25 | 2.88E-02 | NDUFB3/NDUFAF6/NDUFB6/COX7B/CHCHD3/NDUFAB1/HADHB/FAHD1/TIMM9/SLC25A3/ATP5L/HIGD1A/NDUFA4/ABCB8/NDUFA8/SLC25A6/MICU2/AFG3L2/MRPL30/NDUFA1/UQCRHL/MRPL22/MTFP1/ATP5C1/MRPL47 |
| CC | GO:0016235/aggresome | 5 | 2.93E-02 | GIT1/CABIN1/RAB11B/SEC62/XRN2 |
| CC | GO:0016607/nuclear speck | 14 | 2.93E-02 | CSNK1A1/SRP54/CIR1/DDX46/SETD1B/GATAD2A/SLU7/THOC7/FAM76B/PSME4/SF3A2/WBP4/EP400/NRIP1 |
| CC | GO:0005747/mitochondrial respiratory chain complex I | 6 | 3.03E-02 | NDUFA4/NDUFB3/NDUFB6/NDUFA8/NDUFAB1/NDUFA1 |
| CC | GO:0031463/Cul3-RING ubiquitin ligase complex | 7 | 3.26E-02 | KBTBD2/KLHL17/KCTD17/KLHL22/KLHL24/SPOPL/KLHL2 |
| CC | GO:0016592/mediator complex | 5 | 3.55E-02 | MED15/MED17/MED12/MED24/CCNC |
| CC | GO:0048471/perinuclear region of cytoplasm | 32 | 3.97E-02 | CYB5R4/GALNT1/NRBP1/CHERP/COPS5/CSF1/SLC39A13/BAG5/ALDH1A2/ATN1/HECTD3/NDRG1/EHD1/ACSL3/INF2/MAP2K2/RAB4A/RNF207/ITGA3/CDK7/AMBRA1/PNPLA8/BNIP2/MAP1S/EIF4A2/TSC2/RAP1A/PPP1R13B/VAMP2/SRCAP/DNAJB6/SPAST |
| CC | GO:0005913/cell-cell adherens junction | 19 | 4.34E-02 | ZC3H15/USP8/EPS15L1/ABI1/KLC2/RAB1A/CHMP2B/TNKS1BP1/PFN1/CDC42EP1/HNRNPK/SH3GLB2/BAG3/PAK4/RAB11B/NDRG1/SSX2IP/EHD1/SH3GL1 |
| CC | GO:0005778/peroxisomal membrane | 6 | 4.35E-02 | PNPLA8/PEX2/MAP2K2/HSD17B4/ACSL4/ACSL3 |
| CC | GO:0035097/histone methyltransferase complex | 4 | 4.76E-02 | MEN1/KMT2D/SETD1B/KMT2B |
| MF | GO:0005515/protein binding | 417 | 4.95E-14 | LSM8/LSM6/PPP2R5D/ZNF639/MED25/MED24/BRPF3/CUL9/ADAM8/LSM1/PIK3CD/MED12/VPS41/SKP1/PITPNM1/UNC13D/MED15/MED17/PIDD1/SH3GL1/ORAI1/ADSS/RAD23A/HSD17B12/CHCHD3/UBA5/DUSP12/HADHB/PFN1/CDC42EP1/ATN1/HEXIM1/ISY1/TCF3/FRA10AC1/GIT1/DVL3/ZC3H15/ZC3H18/SPHK2/PAIP2/MAP2K2/SPTSSA/ZNF524/DVL1/HGS/MAP4/H3F3A/RERE/TAF1C/HTATIP2/TCOF1/NAP1L1/DAXX/GUCY1A3/USP15/ARHGEF1/TP53/ZNF341/PDCL3/PAPD4/BNIP2/EIF4A2/TXN/PFDN4/THOC7/SPOPL/CSF1/ABI1/BCCIP/ITM2B/C19ORF66/PPP2CB/SOS2/RAB11B/DHX15/LIMD2/WDR11/ETFA/PPP1R18/HIST1H2BC/AIMP1/HIST1H2BF/SF3A2/SF3A1/SGTA/KIAA1143/PHF19/PLEKHF2/ARAF/PLSCR3/ZMYND19/MAT2B/LRP8/ZNF764/MEGF6/CHERP/PRPF4B/AP1G2/CNOT3/NDUFAB1/RELL2/NLRC5/FAM49B/MFHAS1/UBXN2B/TIMM9/SMARCD1/HDAC10/MTA1/CORO7/ARID1A/NABP2/VAMP2/XRN2/EP400/DCUN1D5/ARFGAP1/TRAF2/ADORA2A/SETD1B/CCNG1/OAZ2/FBXW9/HNRNPK/NIPBL/FBXW5/ZNF326/RPL9/JUND/HNRNPD/ARHGDIA/FBXO46/VAV3/CCPG1/MYO1C/MICU2/MPHOSPH10/MYL12A/EHMT2/RBMX/CTC1/UIMC1/HDAC2/TSC2/SMARCC2/FBXO33/DNAJB6/HDAC7/ZNF410/PTGES3/E2F4/LYAR/LRRC8C/NIPSNAP3A/EDC4/PIP5K1C/RGP1/SKAP2/RPS3A/PEX2/GTF2A2/LRWD1/LOXL2/ANO6/CASP2/RTEL1/SIT1/STXBP3/CDK7/SH2D3C/IPO5/UCHL3/VPS29/KMT2D/KMT2B/HAUS1/GPSM3/PPM1A/CCDC120/CHD1L/CALML3/SHISA5/GTF3C6/BRK1/EHD1/SELPLG/VPS36/WDTC1/TMC8/MORN2/NCDN/RAF1/GDPD5/CDC25B/SCFD1/MIS18BP1/JAK3/RCN1/GGA3/RCN2/PRR14/LPAR2/ERLEC1/RAB1A/C1ORF109/GBF1/CD46/FAM103A1/RAB21/RARG/PIM1/RAD9A/PRDM8/PNKP/CD37/TRAPPC13/HUWE1/RAB18/FBXL8/LRCH4/CEP164/BOP1/PXN/KRAS/ACD/SERINC1/FBXO4/NUP54/SRP54/CAMLG/BECN1/SMAD3/RNF207/SKI/AFG3L2/HNRNPA1/PTPN12/RPF1/CCT4/GRK6/RIT1/KLF2/PPARD/RAB5B/COPS5/VBP1/MLH1/ANKRD10/SHOC2/NFKB2/SENP5/WBP4/CANX/BAG5/MCEE/BAG3/ACTR8/AGAP2/FANCC/ABCE1/SLC25A6/RAB4A/MBD4/FAM76B/CCNC/LENG8/MBD2/ZBTB26/GTF2B/SCAP/SENP2/SRCAP/MAP3K12/USP8/SLC39A13/KIAA0196/SHFM1/PPP6R1/KLC2/HPRT1/CIC/SUMO2/SPANXA1/CIR1/RB1CC1/GATAD2A/MLLT1/CC2D1B/HBP1/RPIA/MLLT6/TPRKB/CSNK1A1/RAB2A/GDI1/PDS5B/AMBRA1/CISH/YAF2/TOM1L2/RAB35/UBXN8/TJP3/RBM17/FAM126A/PLXNA3/NRBP1/KLHL2/CBX6/TAPBP/TBC1D15/GATA3/RABGEF1/LOX/AP5Z1/SCAMP2/TNFRSF14/PSMA1/IGSF8/PSMA6/RIOK3/SLU7/SNRPE/SNRPG/ZFAND6/GPN3/SYVN1/SNX6/MAP4K2/ELK1/SNX4/TRRAP/ELK3/SRF/COMMD8/FXYD7/CHMP2B/GFM2/PELP1/HECTD3/NDRG1/SSX2IP/RUNX1/EIF3M/MMADHC/CCDC24/EVL/ITGA3/INTS12/SUGT1/INTS10/FOXP4/AK6/URM1/PSMC6/TXNDC16/PSMC1/NELFA/RFX1/KCTD17/RHOT1/RHOT2/RAP1A/RAP1B/EPS15L1/MEN1/RMND5A/MAPKAP1/PAK4/RANBP6/KLHL22/RANBP3/CNTNAP1/MKL1/TNIP1/CHUK/EFR3B/ANKRA2/LDB1/MINK1/UBE2B/IFNAR1/NRIP1/ZMIZ2/LARP7/CD82/COMMD3/PSME4/PRKD3/MATR3/SPAST/MOB1B/LMF2/HAT1/SH3GLB2/MAP3K2/IL2RG/RSL24D1/ZC3H12D/PIK3R2/NDUFA4/DTX3/CENPP/SIRT6/SCOC/RGS14/ATXN1/RPAP3/ATXN3/PSMG2/MAP1S/TMEM43/ZC3H11A/PPP1R13B/SH3BP1/ACTR10 |
| MF | GO:0019003/GDP binding | 10 | 1.48E-04 | RAB2A/SRP54/KRAS/RAB5B/RAB18/RAB4A/RAB35/RAB11B/RAP1B/RAB21 |
| MF | GO:0043621/protein self-association | 9 | 2.54E-04 | ATXN1/SPTBN5/TMEM43/LDB1/TP53/ZNF639/VPS41/VAMP2/ADAM8 |
| MF | GO:0003924/GTPase activity | 21 | 4.54E-04 | RAB2A/SRP54/GPN3/RAB5B/RAB4A/RAB1A/RABL2B/GFM2/KRAS/RAB18/RAC2/RAB35/RAB11B/RHOT1/RAP1A/RHEB/RHOT2/RIT1/RAP1B/SAR1B/RAB21 |
| MF | GO:0003682/chromatin binding | 29 | 6.88E-04 | ELK1/MLH1/NFKB2/CIC/MEN1/NIPBL/PELP1/GATA3/SMARCD1/HNRNPD/TOP2B/LOXL2/LRWD1/DNAJC2/LDB1/TP53/MED12/MTA1/SIRT6/SKI/MBD2/RBMX/HDAC2/SMARCC2/NELFA/EP400/RERE/HDAC7/ATAD2B |
| MF | GO:0044822/poly(A) RNA binding | 64 | 7.00E-04 | FBRSL1/PRPF4B/METAP2/CHERP/LSM8/LSM6/LYAR/TCOF1/NAP1L1/RBM7/CANX/RSRC2/RPS3A/FAM103A1/LSM1/DNAJC2/SAMD4B/ARHGEF1/ZC3H7A/RRP36/HUWE1/LARP7/EIF4A2/IPO5/TXN/ATP5C1/MATR3/XRN2/SNRPG/ZC3H4/BCCIP/BOP1/DIDO1/HADHB/SUMO2/PFN1/DDX46/HNRNPK/PELP1/ZNF326/CCDC124/NGRN/BRIX1/DHX15/HNRNPD/ISY1/UNK/SRP54/ZC3H15/ZC3H18/PNO1/MPHOSPH10/GRSF1/SF3A2/SF3A1/HNRNPA1/RBMX/RPF1/MRPL22/CCT4/HDAC2/PSMC1/MAP4/ZC3H11A |
| MF | GO:0051082/unfolded protein binding | 13 | 7.67E-04 | PTGES3/SYVN1/AFG3L2/CANX/ERLEC1/SCAP/TAPBP/CCT4/HEATR3/PFDN4/HSP90AB3P/AAMP/DNAJB6 |
| MF | GO:0003713/transcription coactivator activity | 21 | 9.41E-04 | PPARD/HTATIP2/COPS5/MED12/MTA1/ARID1A/NFKB2/CDK7/NRIP1/YAF2/GATA3/MED17/JUND/GTF2A2/SMARCD1/SMARCC2/BCL9L/MKL1/USP16/TCF3/SRCAP |
| MF | GO:0005525/GTP binding | 27 | 2.31E-03 | ADSS/GPN3/RAB5B/RAB1A/GFM2/KRAS/RAC2/MFHAS1/RAB11B/GUCY1A3/RHOBTB1/EHD1/SAR1B/AGAP2/RAB21/RAB2A/SRP54/RAB4A/RABL2B/RAB18/RAB35/RHOT1/RAP1A/RHOT2/RHEB/RIT1/RAP1B |
| MF | GO:0003714/transcription corepressor activity | 17 | 3.71E-03 | LDB1/LMCD1/MTA1/SIRT6/SKI/ELK3/RLIM/DAXX/NRIP1/CIR1/ATN1/YAF2/MAP3K10/HSF4/LOXL2/RUNX1/HDAC7 |
| MF | GO:0008565/protein transporter activity | 9 | 5.02E-03 | VPS29/AP1G2/RAB4A/IPO5/RANBP6/TIMM9/RAP1A/COX18/SEC62 |
| MF | GO:0004709/MAP kinase kinase kinase activity | 5 | 8.00E-03 | MAP3K2/ARAF/MAP3K10/RAF1/MAP3K12 |
| MF | GO:0016887/ATPase activity | 15 | 8.26E-03 | KIFC2/ABCA7/ABCE1/MLH1/DNAH1/AK6/PSMC6/CHD1L/ATP13A1/EIF4A2/PSMC1/ATP5C1/ATP5L/KIF21B/ATAD2B |
| MF | GO:0019903/protein phosphatase binding | 8 | 8.47E-03 | TRAF2/TP53/PPP6R1/SHOC2/HSF4/JAK3/AP3B1/PIK3R2 |
| MF | GO:0051087/chaperone binding | 9 | 1.01E-02 | BAG5/SYVN1/BAG3/PFDN4/TIMM9/TP53/RNF207/OGDH/DNAJB6 |
| MF | GO:0005524/ATP binding | 73 | 1.26E-02 | ADCY3/KIFC2/PRPF4B/NRBP1/MLH1/PIP5K1C/CAD/NLRC5/PAK4/CUL9/ACTR8/TOP2B/RTEL1/CHUK/ABCE1/SGK2/PIK3CD/TP53/PIM1/MINK1/ABCC10/CDK7/UBE2B/PNPLA8/PNKP/PAPD4/RIOK3/EIF4A2/MAP3K10/SRCAP/PCCA/EP400/PRKD3/SPAST/MAP3K12/NRBP2/BTAF1/ABCA7/GCLC/MAP4K2/DNAH1/UBA5/ITM2B/KRAS/DDX46/CHD1L/MAP3K2/DHX15/RUNX1/ACSL4/EHD1/ACSL3/KIF21B/CSNK1A1/PDS5B/ABCB8/MYO1C/SPHK2/MAP2K2/RAF1/AFG3L2/AK6/PSMC6/ATP13A1/CCT4/ATP2A3/ARAF/HSP90AB3P/PSMC1/GRK6/JAK3/FUK/ATAD2B |
| MF | GO:0004175/endopeptidase activity | 7 | 1.44E-02 | SENP2/ACE/PSMA6/CD46/RCE1/FAM76B/SENP5 |
| MF | GO:0008134/transcription factor binding | 19 | 1.89E-02 | PPARD/E2F4/TP53/PIM1/MED25/SMAD3/PAXBP1/DAXX/SRF/GTF2B/HDAC2/PELP1/GATA3/JUND/GTF2A2/HNRNPD/PPP1R13B/RUNX1/TCF3 |
| MF | GO:0003723/RNA binding | 31 | 2.08E-02 | HNRNPA1L2/CHERP/LSM8/LSM6/SETD1B/UNC50/RLIM/HNRNPK/RPS3A/RAE1/C19ORF66/RPL9/BRIX1/HNRNPD/HBP1/FAM103A1/SAMD4B/GRSF1/THUMPD2/RBMX/SF3A1/HNRNPA1/RPF1/ATXN1/PSMA1/PSMA6/LARP7/THOC7/SNRPE/RBM17/SNRPG |
| MF | GO:0098505/G-rich strand telomeric DNA binding | 3 | 2.54E-02 | NABP2/HNRNPA1/CTC1 |
| MF | GO:0016929/SUMO-specific protease activity | 3 | 2.54E-02 | SENP2/FAM76B/SENP5 |
| MF | GO:0008270/zinc ion binding | 57 | 2.79E-02 | PPARD/AGTPBP1/CAD/RLIM/WBP4/RNF141/BRPF3/PEX2/GATA3/CUL9/TIMM9/RABGEF1/USP16/ADAM8/KDM7A/RARG/TP53/UBR3/MTA1/ZSWIM8/VPS41/GTF2B/MTF2/ZMIZ2/COMMD3/SLU7/NEURL3/MATR3/PMPCB/ZFAND6/TRAF2/KMT2D/SYVN1/KMT2B/DUSP12/DIDO1/PXN/ACE/GATAD2A/LIMD2/MLLT6/DTX3/LMCD1/SMAD3/RNF207/SKI/SIRT6/INTS12/SF3A2/AFG3L2/EHMT2/TRIM62/ZNF22/MAN2C1/PHF19/YAF2/RERE |
| MF | GO:0008137/NADH dehydrogenase (ubiquinone) activity | 6 | 3.16E-02 | NDUFA4/NDUFB3/NDUFB6/NDUFA8/NDUFAB1/NDUFA1 |
| MF | GO:0042826/histone deacetylase binding | 9 | 3.55E-02 | WDTC1/NIPBL/ANKRA2/HNRNPD/HDAC10/RAD9A/TOP2B/SRF/NRIP1 |
| MF | GO:0019901/protein kinase binding | 22 | 4.00E-02 | IBTK/TRAF2/TP53/SMAD3/RAD9A/SKI/DAXX/RGS14/PXN/CDC25B/DVL1/BAG5/FBXW5/MAP3K2/TOM1L2/RB1CC1/MAPKAP1/RHEB/ACSL3/TPRKB/HDAC7/MAP3K12 |
| MF | GO:0070412/R-SMAD binding | 4 | 4.09E-02 | MEN1/PPM1A/RANBP3/SMAD3 |
| MF | GO:0017025/TBP-class protein binding | 4 | 4.09E-02 | PSMC6/PSMC1/GTF2A2/GTF2B |
| MF | GO:0098641/cadherin binding involved in cell-cell adhesion | 18 | 4.16E-02 | ZC3H15/USP8/EPS15L1/ABI1/KLC2/RAB1A/CHMP2B/TNKS1BP1/PFN1/CDC42EP1/HNRNPK/SH3GLB2/BAG3/PAK4/RAB11B/NDRG1/EHD1/SH3GL1 |
| MF | GO:0032403/protein complex binding | 14 | 4.33E-02 | GIT1/NDUFA4/TRAF2/NDUFA8/ABI1/SCAP/KRAS/RPAIN/BAG3/SLC25A3/KCTD17/RAP1A/BRK1/RAP1B |

Supplementary Table 5 KEGG pathway and DEGs at admission

| KEGG PATHWAY | Count | PValue | Genes |
| --- | --- | --- | --- |
| hsa04071:Sphingolipid signaling pathway | 30 | 1.84E-08 | PRKCZ/TRAF2/PPP2R3B/ADORA3/SGMS2/SGPP2/PPP2R5D/PTEN/ASAH1/S1PR1/KRAS/S1PR4/BCL2/PPP2CB/PLCB1/PIK3R2/PPP2R1A/PLD1/SPTLC1/ROCK1/SPTLC2/PIK3CB/RELA/PIK3CD/TP53/RAF1/MAPK14/ABCC1/PPP2R3C/SMPD2 |
| hsa05221:Acute myeloid leukemia | 16 | 1.41E-05 | PPARD/TCF7/PIK3CB/FLT3/RELA/STAT5B/PIK3CD/PML/PIM1/RAF1/PIM2/KRAS/SOS2/MTOR/CHUK/PIK3R2 |
| hsa04144:Endocytosis | 38 | 3.54E-05 | VPS29/ARFGAP1/PRKCZ/TSG101/CAPZA2/STAM2/PML/EEA1/SNX3/AMPH/CHMP2B/GBF1/SH3GLB2/SH3GLB1/ZFYVE16/SPG20/KIAA1033/SPG21/IL2RG/WIPF1/EHD1/AGAP2/GIT1/PLD1/IL2RA/VPS45/RAB4A/VTA1/KIF5C/SMAD3/LDLRAP1/ACAP2/RAB5A/GRK6/GRK4/BIN1/VPS26A/DNM2 |
| hsa05231:Choline metabolism in cancer | 21 | 7.08E-05 | CHKA/SLC44A1/PLD1/PIK3CB/PIK3CD/RAF1/LYPLA1/CHPT1/RALGDS/DGKA/PLA2G4A/HIF1A/KRAS/PLCG1/TSC2/SOS2/SLC22A4/DGKZ/RHEB/MTOR/PIK3R2 |
| hsa04660:T cell receptor signaling pathway | 20 | 1.90E-04 | VAV3/PIK3CB/CD3E/RELA/PIK3CD/RAF1/CDK4/CARD11/LAT/KRAS/PLCG1/MAPK14/SOS2/MAP3K14/NFATC2/CHUK/TEC/PIK3R2/CD28/NFATC1 |
| hsa04919:Thyroid hormone signaling pathway | 21 | 4.44E-04 | RXRB/PIK3CB/CREBBP/PIK3CD/TP53/MED12/RAF1/MED24/FOXO1/ATP1A1/HIF1A/KRAS/PLCG1/MED16/ITGAV/TSC2/RHEB/PLCD1/MTOR/PLCB1/PIK3R2 |
| hsa05162:Measles | 23 | 4.84E-04 | RAB9A/IL2RA/TBK1/PIK3CB/CD3E/RELA/STAT5B/PIK3CD/TP53/TLR2/TLR4/CDK4/IFNAR1/TLR9/IKBKE/CCND2/IL2RG/RCHY1/JAK3/CHUK/IFNGR1/PIK3R2/CD28 |
| hsa05161:Hepatitis B | 24 | 6.69E-04 | LAMTOR5/TBK1/PIK3CB/RELA/CREBBP/PIK3CD/STAT5B/TP53/TLR2/RAF1/ELK1/TLR4/RB1/CDK4/PTEN/IFNAR1/IKBKE/KRAS/BCL2/APAF1/NFATC2/CHUK/PIK3R2/NFATC1 |
| hsa05222:Small cell lung cancer | 16 | 1.93E-03 | TRAF1/TRAF2/RXRB/PIK3CB/RELA/PIK3CD/TP53/ITGA3/RB1/CDK4/PTEN/ITGAV/BCL2/APAF1/CHUK/PIK3R2 |
| hsa05166:HTLV-I infection | 34 | 1.98E-03 | ADCY3/IL1R2/STAT5B/ELK1/NFKB2/TRRAP/KRAS/RANBP3/IL2RG/NFATC2/TCF3/TBPL1/CHUK/LTA/PIK3R2/NFATC1/IL2RA/ANAPC5/PIK3CB/CD3E/RELA/CREBBP/PIK3CD/TP53/SMAD3/RB1/VDAC2/CDK4/DVL1/CCND2/POLD1/JAK3/MAP3K14/WNT7A |
| hsa05142:Chagas disease (American trypanosomiasis) | 18 | 2.35E-03 | PPP2R1A/CFLAR/PIK3CB/CD3E/RELA/PIK3CD/TLR2/SMAD3/TLR4/TLR6/TLR9/ACE/MAPK14/PPP2CB/PLCB1/CHUK/IFNGR1/PIK3R2 |
| hsa04064:NF-kappa B signaling pathway | 16 | 2.44E-03 | TRAF1/TRAF2/CFLAR/LY96/RELA/BCL2A1/TLR4/NFKB2/CARD11/LAT/PLCG1/BCL2/MAP3K14/LTB/CHUK/LTA |
| hsa05200:Pathways in cancer | 47 | 2.56E-03 | ADCY3/TRAF1/TRAF2/PPARD/STAT5B/PML/EGLN2/FOXO1/NFKB2/PTEN/FLT3LG/KRAS/BCL2/ITGAV/RASGRP2/SOS2/RALB/AXIN2/PLCB1/GNG5/CHUK/PIK3R2/TCF7/CTBP1/ARHGEF1/BCR/ROCK1/PIK3CB/RXRB/FLT3/RELA/CREBBP/PIK3CD/TP53/RAF1/SMAD3/ITGA3/RB1/HGF/CDK4/RALGDS/DVL1/VEGFB/HIF1A/PLCG1/MTOR/WNT7A |
| hsa00562:Inositol phosphate metabolism | 14 | 2.70E-03 | IMPA2/PIK3CB/PIK3C2B/PIK3CD/PI4KA/ITPKB/PTEN/ISYNA1/PLCG1/PLCH2/INPP5E/PLCD1/PLCB1/MTMR6 |
| hsa05215:Prostate cancer | 16 | 2.74E-03 | TCF7/PIK3CB/RELA/CREBBP/PIK3CD/TP53/RAF1/FOXO1/RB1/PTEN/KRAS/BCL2/SOS2/MTOR/CHUK/PIK3R2 |
| hsa05220:Chronic myeloid leukemia | 14 | 3.07E-03 | CTBP1/KRAS/BCR/PIK3CB/RELA/SOS2/PIK3CD/STAT5B/TP53/RAF1/RB1/CDK4/CHUK/PIK3R2 |
| hsa04068:FoxO signaling pathway | 21 | 3.09E-03 | GABARAPL1/SGK3/PIK3CB/CREBBP/PIK3CD/RAF1/SMAD3/FOXO1/CCNG2/PTEN/S1PR1/KRAS/CSNK1E/CCND2/S1PR4/MAPK14/SOS2/KLF2/AGAP2/CHUK/PIK3R2 |
| hsa04070:Phosphatidylinositol signaling system | 17 | 3.16E-03 | IMPA2/PIK3CB/PIK3C2B/PIK3CD/PI4KA/PPIP5K1/ITPKB/ITPR3/PTEN/DGKA/PLCG1/DGKZ/INPP5E/PLCD1/PLCB1/MTMR6/PIK3R2 |
| hsa05212:Pancreatic cancer | 13 | 3.62E-03 | KRAS/PIK3CB/RELA/PIK3CD/TP53/RALB/RAF1/SMAD3/RB1/CDK4/CHUK/RALGDS/PIK3R2 |
| hsa04115:p53 signaling pathway | 13 | 4.68E-03 | RFWD2/PPM1D/CCND2/CD82/SHISA5/TSC2/TP53/RCHY1/APAF1/RRM2B/CDK4/CCNG2/PTEN |
| hsa05213:Endometrial cancer | 11 | 5.67E-03 | TCF7/KRAS/PIK3CB/PIK3CD/SOS2/TP53/RAF1/ELK1/AXIN2/PTEN/PIK3R2 |
| hsa04152:AMPK signaling pathway | 19 | 6.02E-03 | RAB2A/PPP2R1A/PPP2R3B/PFKL/PIK3CB/PPP2R5D/PIK3CD/FBP1/FOXO1/RPTOR/PPP2CB/TSC2/FASN/EEF2K/RHEB/CAB39/MTOR/PPP2R3C/PIK3R2 |
| hsa04620:Toll-like receptor signaling pathway | 17 | 6.93E-03 | LY96/TBK1/PIK3CB/RELA/TLR1/PIK3CD/TLR2/TLR4/TLR5/TLR6/TLR9/IFNAR1/IKBKE/CD86/MAPK14/CHUK/PIK3R2 |
| hsa04630:Jak-STAT signaling pathway | 21 | 7.62E-03 | IL9R/IL2RA/PIK3CB/STAM2/CREBBP/IL21R/STAT5B/PIK3CD/PIM1/IL11RA/CISH/IFNAR1/CCND2/IL10RA/IL4R/SOS2/IL2RG/JAK3/IL13RA1/IFNGR1/PIK3R2 |
| hsa00600:Sphingolipid metabolism | 10 | 8.76E-03 | ACER3/SPTLC1/SPTLC2/SGMS2/SGPP2/CERK/B4GALT6/SMPD4/ASAH1/SMPD2 |
| hsa05321:Inflammatory bowel disease (IBD) | 12 | 9.10E-03 | IL4R/RELA/IL21R/TLR2/SMAD3/RORC/IL2RG/TLR4/TLR5/FOXP3/IFNGR1/NFATC1 |
| hsa05230:Central carbon metabolism in cancer | 12 | 9.10E-03 | KRAS/HIF1A/PFKL/PIK3CB/FLT3/PIK3CD/TP53/RAF1/MTOR/PTEN/PDHB/PIK3R2 |
| hsa04910:Insulin signaling pathway | 20 | 9.34E-03 | PRKCZ/EXOC7/PIK3CB/PHKB/PIK3CD/MKNK2/FBP1/RAF1/ELK1/FOXO1/RPTOR/KRAS/SOS2/PPP1R3F/TSC2/FASN/RHEB/PTPN1/MTOR/PIK3R2 |
| hsa05223:Non-small cell lung cancer | 11 | 9.67E-03 | KRAS/PLCG1/PIK3CB/RXRB/PIK3CD/SOS2/TP53/RAF1/RB1/CDK4/PIK3R2 |
| hsa00511:Other glycan degradation | 6 | 9.90E-03 | MAN2C1/MAN2B2/HEXB/FUCA2/FUCA1/MANBA |
| hsa04330:Notch signaling pathway | 10 | 1.01E-02 | MFNG/CTBP1/CIR1/PSEN1/DTX1/CREBBP/APH1B/ADAM17/LFNG/DVL1 |
| hsa05214:Glioma | 12 | 1.02E-02 | KRAS/PLCG1/PIK3CB/PIK3CD/SOS2/TP53/RAF1/RB1/MTOR/CDK4/PTEN/PIK3R2 |
| hsa04722:Neurotrophin signaling pathway | 18 | 1.03E-02 | PIK3CB/RELA/PIK3CD/TP53/RAF1/MAGED1/IRAK3/KRAS/PLCG1/PSEN1/BCL2/MAPK14/SOS2/RIPK2/RAP1A/RAP1B/ARHGDIA/PIK3R2 |
| hsa05211:Renal cell carcinoma | 12 | 1.14E-02 | KRAS/HIF1A/PIK3CB/PIK3CD/CREBBP/SOS2/RAP1A/EGLN2/RAF1/RAP1B/HGF/PIK3R2 |
| hsa05120:Epithelial cell signaling in Helicobacter pylori infection | 12 | 1.28E-02 | ATP6V1C1/GIT1/ATP6V0E2/PLCG1/MAPK14/RELA/ADAM17/ATP6V1H/ATP6V1B2/MAP3K14/ATP6V1D/CHUK |
| hsa04066:HIF-1 signaling pathway | 15 | 1.49E-02 | PIK3CB/RELA/CREBBP/PIK3CD/MKNK2/EGLN2/TLR4/PDHB/HIF1A/PLCG1/BCL2/ENO2/MTOR/IFNGR1/PIK3R2 |
| hsa04662:B cell receptor signaling pathway | 12 | 1.58E-02 | CARD11/VAV3/KRAS/PIK3CB/RELA/PIK3CD/SOS2/RAF1/NFATC2/CHUK/NFATC1/PIK3R2 |
| hsa04062:Chemokine signaling pathway | 24 | 1.59E-02 | ADCY3/PRKCZ/VAV3/ROCK1/PIK3CB/RELA/STAT5B/PIK3CD/RAF1/CXCR3/KRAS/CXCR5/CCR4/SOS2/RASGRP2/GRK6/RAP1A/GRK4/RAP1B/JAK3/PLCB1/GNG5/CHUK/PIK3R2 |
| hsa04917:Prolactin signaling pathway | 12 | 1.93E-02 | KRAS/CCND2/PIK3CB/MAPK14/RELA/PIK3CD/STAT5B/SOS2/GALT/RAF1/CISH/PIK3R2 |
| hsa04210:Apoptosis | 11 | 1.93E-02 | TRAF2/CFLAR/PIK3CB/RELA/BCL2/PIK3CD/TP53/APAF1/MAP3K14/CHUK/PIK3R2 |
| hsa05210:Colorectal cancer | 11 | 1.93E-02 | TCF7/KRAS/PIK3CB/BCL2/PIK3CD/TP53/RAF1/SMAD3/AXIN2/RALGDS/PIK3R2 |
| hsa04611:Platelet activation | 18 | 2.16E-02 | ADCY3/ORAI1/PRKCZ/TBXAS1/ARHGEF1/ROCK1/PIK3CB/PIK3CD/ARHGAP35/MYL12A/ITPR3/PLA2G4A/MAPK14/RASGRP2/RAP1A/RAP1B/PLCB1/PIK3R2 |
| hsa04151:PI3K-Akt signaling pathway | 38 | 2.41E-02 | PPP2R3B/PPP2R5D/CSF1/TLR2/TLR4/PTEN/KRAS/BCL2/ITGB7/ITGAV/IL4R/PPP2CB/SOS2/IL2RG/GNG5/CHUK/PIK3R2/PPP2R1A/IL2RA/SGK3/PIK3CB/RELA/PIK3CD/TP53/PKN2/RAF1/ITGA3/HGF/CDK4/RPTOR/IFNAR1/VEGFB/CCND2/TSC2/RHEB/JAK3/MTOR/PPP2R3C |
| hsa00604:Glycosphingolipid biosynthesis - ganglio series | 5 | 2.41E-02 | ST3GAL1/ST6GALNAC6/ST6GALNAC3/ST6GALNAC4/HEXB |
| hsa05168:Herpes simplex infection | 23 | 2.43E-02 | TRAF1/TRAF2/TAF6/TBK1/RELA/C5/CREBBP/PML/TP53/TLR2/HCFC1/TNFRSF14/DAXX/CD74/TLR9/POLR2A/IFNAR1/IKBKE/TAP1/TBPL1/CHUK/IFNGR1/LTA |
| hsa05132:Salmonella infection | 13 | 2.47E-02 | DYNC1LI1/ROCK1/RELA/PKN2/TLR4/TLR5/KLC2/FLNA/PFN1/NLRC4/MAPK14/CASP1/IFNGR1 |
| hsa05169:Epstein-Barr virus infection | 17 | 2.48E-02 | TRAF2/TBK1/PIK3CB/RELA/PIK3CD/TP53/NFKB2/RB1/CD44/MAPK14/BCL2/CD58/JAK3/ENTPD1/MAP3K14/CHUK/PIK3R2 |
| hsa04014:Ras signaling pathway | 27 | 2.53E-02 | TBK1/CSF1/ELK1/KRAS/RASAL3/SOS2/RASGRP2/RALB/RASA3/GNG5/CHUK/PIK3R2/PLD1/PIK3CB/RELA/PIK3CD/RAF1/HGF/RALGDS/VEGFB/LAT/PLA2G4A/PLCG1/RAB5A/RAP1A/RAP1B/SYNGAP1 |
| hsa01100:Metabolic pathways | 112 | 2.78E-02 | SGMS2/PGD/NDUFAB1/ITPKB/OGDH/PDHB/ST3GAL1/ACSS1/AGPS/NT5C2/ST3GAL6/IDUA/CRLS1/PLD1/SPTLC1/SPTLC2/CMAS/FBP1/HAL/CYP2E1/CHPT1/GLCE/LPCAT4/DLD/ATP5C1/CHKA/SRM/GLUD1/HSD17B12/RRM2B/ATP6V1B2/HADHA/HADHB/CEPT1/ISYNA1/MUT/PLCH2/IDH1/B4GALT6/B4GALT3/GALT/AK6/PLA2G4A/ATP6V0E2/GBE1/GGT7/PLCG1/POLD1/DNMT1/AHCYL2/IDI1/FPGS/SAT1/IMPA2/KYNU/HEXB/ALG6/CAD/ASAH1/ST6GALNAC6/ST6GALNAC3/ST6GALNAC4/XYLT2/P4HA1/PIGB/PLCB1/PFKL/PIK3C2B/POLR1A/PI4KA/ATP6V1H/ATP6V1D/PIGO/ATP6V1C1/COQ2/UGP2/NDUFB3/GALNT1/NDUFB6/GALNT7/ADPGK/HPRT1/AZIN2/ALDH3A2/PFAS/POLR2A/DGKA/MTHFS/ACSL1/FASN/ENO2/INPP5E/PLCD1/HSD17B4/MTMR6/ACSL4/PAPSS2/ACSL3/POLR3F/TBXAS1/ASMT/MAN1C1/MPI/CSGALNACT2/DPM1/DPM2/DGKZ/MBOAT2/LTA4H/DPYD/SMPD4/SMPD2 |
| hsa05152:Tuberculosis | 22 | 3.11E-02 | CEBPG/RELA/CREBBP/TLR1/TLR2/RAF1/ATP6V1H/TLR4/EEA1/TLR6/CD74/TLR9/LAMP2/CLEC4E/IL10RA/BCL2/MAPK14/RAB5A/RIPK2/CLEC7A/APAF1/IFNGR1 |
| hsa04150:mTOR signaling pathway | 10 | 3.23E-02 | PIK3CB/PIK3CD/TSC2/ULK3/RHEB/CAB39/MTOR/PTEN/RPTOR/PIK3R2 |
| hsa04010:MAPK signaling pathway | 29 | 3.26E-02 | TRAF2/ZAK/PPM1A/MKNK2/MAP4K2/MAP4K1/ELK1/NFKB2/DAXX/KRAS/MAP3K2/SOS2/RASGRP2/DUSP16/CHUK/NFATC1/LAMTOR3/RELA/CACNA1I/TP53/RAF1/STK3/FLNA/CDC25B/PLA2G4A/MAPK14/RAP1A/RAP1B/MAP3K14 |
| hsa04015:Rap1 signaling pathway | 25 | 3.30E-02 | ADCY3/PRKCZ/PIK3CB/ADORA2A/CSF1/PIK3CD/SIPA1L3/RAF1/HGF/RALGDS/RGS14/VEGFB/LAT/PRKD2/PFN1/KRAS/PLCG1/MAPK14/RASGRP2/RALB/RAP1A/RAP1B/PLCB1/PRKD3/PIK3R2 |
| hsa04640:Hematopoietic cell lineage | 13 | 3.42E-02 | IL1R2/CD37/IL9R/IL2RA/CD44/CD3E/FLT3/IL4R/CSF1/ITGA3/CD5/IL11RA/FLT3LG |
| hsa04664:Fc epsilon RI signaling pathway | 11 | 3.46E-02 | LAT/PLA2G4A/VAV3/KRAS/PLCG1/PIK3CB/MAPK14/PIK3CD/SOS2/RAF1/PIK3R2 |
| hsa04380:Osteoclast differentiation | 17 | 4.41E-02 | TRAF2/PIK3CB/RELA/CSF1/PIK3CD/NFKB2/IFNAR1/SIRPG/MAPK14/MAP3K14/NFATC2/CHUK/IFNGR1/TEC/TYROBP/PIK3R2/NFATC1 |
| hsa00310:Lysine degradation | 9 | 4.42E-02 | KMT2D/EHMT1/KMT2A/SETD1B/KMT2B/EHMT2/OGDH/ALDH3A2/HADHA |
| hsa04962:Vasopressin-regulated water reabsorption | 8 | 4.96E-02 | ADCY3/DYNC1LI1/RAB5A/VAMP2/DCTN1/AQP3/NSF/ARHGDIA |

Supplementary Table 6 KEGG pathway and DEGs at discharge

| KEGG PATHWAY | Count | PValue | Gene |
| --- | --- | --- | --- |
| hsa04071:Sphingolipid signaling pathway | 23 | 2.35E-08 | PRKCZ/TRAF2/PLD1/TNF/SPTLC1/ROCK1/SGMS2/ROCK2/PIK3CB/SGPP2/RELA/PPP2R5D/PIK3CD/TP53/RAF1/ASAH1/PPP2CA/S1PR4/MAPK14/PPP2CB/MAPK9/ABCC1/PPP2R3C |
| hsa04144:Endocytosis | 27 | 4.76E-05 | VPS29/PRKCZ/USP8/CAPZA2/STAM2/KIAA0196/EEA1/SNX4/ZFYVE16/SPG20/KIAA1033/IL2RG/ITCH/EHD1/AGAP2/GIT1/PLD1/VPS45/VTA1/SMAD3/SMAD2/RAB11FIP3/CHMP1B/GRK6/MDM2/BIN1/VPS26A |
| hsa05142:Chagas disease (American trypanosomiasis) | 16 | 8.22E-05 | CFLAR/TNF/PIK3CB/CD3E/RELA/PIK3CD/MAP2K4/SMAD3/SMAD2/ACE/PPP2CA/MAPK14/PPP2CB/MAPK9/IFNGR1/CHUK |
| hsa04010:MAPK signaling pathway | 26 | 2.72E-04 | TRAF2/TNF/PDGFB/ZAK/PPM1A/MAP4K2/CACNB1/MAP4K1/ELK1/SOS2/RASGRP2/RASA1/CHUK/NFATC1/PTPN7/RELA/CACNA1I/MAP2K4/TP53/RAF1/STK3/CDC25B/MAPK14/MAPK9/MAP3K14/DUSP7 |
| hsa05160:Hepatitis C | 17 | 4.16E-04 | TRAF2/TNF/TBK1/PIK3CB/RELA/PIK3CD/TP53/RAF1/IFNAR1/IKBKE/MAPK14/PPP2CA/PPP2CB/SOS2/MAPK9/IRF3/CHUK |
| hsa05210:Colorectal cancer | 11 | 5.12E-04 | TCF7/PIK3CB/PIK3CD/TP53/RAF1/SMAD3/MAPK9/SMAD2/AXIN2/RALGDS/APC |
| hsa05200:Pathways in cancer | 34 | 6.29E-04 | TRAF1/TRAF2/PPARD/PDGFB/FOXO1/FLT3LG/WNT1/ITGAV/RASGRP2/SOS2/AXIN2/RUNX1/CHUK/FH/APC/WNT10A/TCF7/ARHGEF1/ROCK1/PIK3CB/RXRB/FLT3/ROCK2/RELA/PIK3CD/TP53/SMAD3/RAF1/ITGA3/SMAD2/HGF/RALGDS/MDM2/MAPK9 |
| hsa05321:Inflammatory bowel disease (IBD) | 11 | 6.64E-04 | TNF/IL18/RELA/IL21R/SMAD3/RORC/IL2RG/SMAD2/FOXP3/IFNGR1/NFATC1 |
| hsa04660:T cell receptor signaling pathway | 14 | 6.85E-04 | TNF/VAV3/PIK3CB/CD3E/RELA/PIK3CD/RAF1/LAT/MAPK14/SOS2/MAP3K14/CHUK/TEC/NFATC1 |
| hsa05120:Epithelial cell signaling in Helicobacter pylori infection | 11 | 9.61E-04 | ATP6V1C1/GIT1/ATP6V0E2/ADAM10/MAPK14/RELA/MAP2K4/MAPK9/MAP3K14/ATP6V1D/CHUK |
| hsa05221:Acute myeloid leukemia | 10 | 9.97E-04 | PPARD/TCF7/PIK3CB/FLT3/RELA/PIK3CD/SOS2/RAF1/RUNX1/CHUK |
| hsa04668:TNF signaling pathway | 14 | 1.30E-03 | TRAF1/CFLAR/TRAF2/TNF/PIK3CB/RELA/CSF1/PIK3CD/MAP2K4/MAPK14/MAPK9/ITCH/MAP3K14/CHUK |
| hsa05169:Epstein-Barr virus infection | 15 | 1.50E-03 | TRAF2/TBK1/PIK3CB/RELA/PIK3CD/MAP2K4/TP53/MAPK14/PSMD1/MAPK9/MDM2/IRF3/PSMD6/MAP3K14/CHUK |
| hsa04210:Apoptosis | 10 | 2.09E-03 | TRAF2/CFLAR/TNF/PIK3CB/RELA/PIK3CD/TP53/APAF1/MAP3K14/CHUK |
| hsa05213:Endometrial cancer | 9 | 2.52E-03 | TCF7/PIK3CB/PIK3CD/SOS2/TP53/RAF1/ELK1/AXIN2/APC |
| hsa05212:Pancreatic cancer | 10 | 2.92E-03 | PIK3CB/RELA/PIK3CD/TP53/RAF1/SMAD3/MAPK9/SMAD2/CHUK/RALGDS |
| hsa05161:Hepatitis B | 16 | 2.92E-03 | TNF/TBK1/PIK3CB/RELA/PIK3CD/MAP2K4/TP53/RAF1/ELK1/IFNAR1/IKBKE/MAPK9/IRF3/APAF1/CHUK/NFATC1 |
| hsa04068:FoxO signaling pathway | 15 | 3.64E-03 | SGK3/PIK3CB/PIK3CD/RAF1/SMAD3/FOXO1/SMAD2/S1PR4/MAPK14/SOS2/MDM2/MAPK9/KLF2/AGAP2/CHUK |
| hsa04664:Fc epsilon RI signaling pathway | 10 | 3.98E-03 | LAT/TNF/VAV3/PIK3CB/MAPK14/PIK3CD/SOS2/MAP2K4/RAF1/MAPK9 |
| hsa04622:RIG-I-like receptor signaling pathway | 10 | 4.84E-03 | TRAF2/IKBKE/TNF/TBK1/MAPK14/RELA/MAPK9/IRF3/AZI2/CHUK |
| hsa05168:Herpes simplex infection | 18 | 4.87E-03 | TRAF1/TRAF2/TNF/TAF6/TBK1/RELA/C5/TP53/HCFC1/TNFRSF14/CD74/POLR2A/IFNAR1/IKBKE/MAPK9/IRF3/IFNGR1/CHUK |
| hsa05222:Small cell lung cancer | 11 | 5.80E-03 | TRAF1/TRAF2/PIK3CB/RXRB/ITGAV/RELA/PIK3CD/TP53/ITGA3/APAF1/CHUK |
| hsa05220:Chronic myeloid leukemia | 10 | 5.84E-03 | PIK3CB/RELA/PIK3CD/SOS2/TP53/RAF1/MDM2/SHC1/RUNX1/CHUK |
| hsa00520:Amino sugar and nucleotide sugar metabolism | 8 | 6.28E-03 | CYB5R4/AMDHD2/CMAS/NPL/GALT/FPGT/NANP/UGP2 |
| hsa04919:Thyroid hormone signaling pathway | 13 | 6.95E-03 | SIN3A/PIK3CB/RXRB/ITGAV/PIK3CD/TP53/MED12/RAF1/MDM2/FOXO1/MED24/RHEB/PLCD1 |
| hsa05231:Choline metabolism in cancer | 12 | 6.99E-03 | SLC44A1/PLD1/PDGFB/PIK3CB/PIK3CD/SOS2/RAF1/DGKZ/MAPK9/RHEB/LYPLA1/RALGDS |
| hsa05215:Prostate cancer | 11 | 7.40E-03 | TCF7/PDGFB/PIK3CB/RELA/PIK3CD/SOS2/TP53/RAF1/FOXO1/MDM2/CHUK |
| hsa04380:Osteoclast differentiation | 14 | 7.68E-03 | TRAF2/TNF/PIK3CB/RELA/CSF1/PIK3CD/IFNAR1/MAPK14/MAPK9/MAP3K14/IFNGR1/CHUK/TEC/NFATC1 |
| hsa04620:Toll-like receptor signaling pathway | 12 | 9.95E-03 | IKBKE/TNF/PIK3CB/TBK1/MAPK14/RELA/PIK3CD/MAP2K4/MAPK9/IRF3/CHUK/IFNAR1 |
| hsa05205:Proteoglycans in cancer | 18 | 1.16E-02 | WNT10A/TNF/ARHGEF1/ROCK1/ROCK2/PIK3CB/PIK3CD/TP53/ELK1/RAF1/RDX/HGF/ITPR2/WNT1/ITGAV/MAPK14/SOS2/MDM2 |
| hsa04917:Prolactin signaling pathway | 9 | 1.66E-02 | PIK3CB/MAPK14/RELA/PIK3CD/SOS2/GALT/RAF1/MAPK9/SHC1 |
| hsa04640:Hematopoietic cell lineage | 10 | 1.92E-02 | CD37/TNF/IL9R/CD3E/FLT3/CSF1/ITGA3/CD5/IL11RA/FLT3LG |
| hsa05162:Measles | 13 | 2.06E-02 | IKBKE/CD3E/PIK3CB/TBK1/RELA/PIK3CD/TP53/IRF3/IL2RG/RCHY1/CHUK/IFNGR1/IFNAR1 |
| hsa04310:Wnt signaling pathway | 13 | 2.66E-02 | CSNK1A1/WNT10A/PPARD/TCF7/ROCK2/TP53/SENP2/WNT1/MAPK9/SIAH1/AXIN2/NFATC1/APC |
| hsa05214:Glioma | 8 | 3.04E-02 | PDGFB/PIK3CB/PIK3CD/SOS2/TP53/RAF1/MDM2/SHC1 |
| hsa05164:Influenza A | 15 | 3.16E-02 | IKBKE/TNF/PIK3CB/TBK1/IL18/MAPK14/RELA/MAP2K4/PIK3CD/RAF1/MAPK9/IRF3/DNAJC3/IFNGR1/IFNAR1 |
| hsa04014:Ras signaling pathway | 18 | 3.36E-02 | PLD1/PDGFB/TBK1/PIK3CB/RELA/CSF1/PIK3CD/RAF1/ELK1/HGF/RALGDS/LAT/RASGRP2/SOS2/MAPK9/SHC1/RASA1/CHUK |
| hsa04662:B cell receptor signaling pathway | 8 | 4.04E-02 | VAV3/PIK3CB/RELA/PIK3CD/SOS2/RAF1/CHUK/NFATC1 |
| hsa00562:Inositol phosphate metabolism | 8 | 4.60E-02 | MINPP1/PIK3CB/PIK3C2B/PLCH2/PIK3CD/INPP5E/PLCD1/MTMR6 |
| hsa04064:NF-kappa B signaling pathway | 9 | 4.86E-02 | TRAF1/LAT/TRAF2/CFLAR/TNF/RELA/MAP3K14/LTB/CHUK |
| hsa05166:HTLV-I infection | 19 | 4.87E-02 | WNT10A/TNF/PDGFB/PIK3CB/CD3E/RELA/PIK3CD/MAP2K4/TP53/SMAD3/ELK1/SMAD2/TRRAP/WNT1/IL2RG/MAP3K14/CHUK/APC/NFATC1 |

Supplementary Table 7 KEGG pathway and DEGs at 1 month after STEMI

| KEGG PATHWAY | Count | PValue | Gene |
| --- | --- | --- | --- |
| hsa04919:Thyroid hormone signaling pathway | 20 | 2.70E-05 | ATP1B3/RXRB/MAP2K2/RXRA/CREBBP/PIK3CD/TP53/MED12/MED24/ATP1A1/AKT1/NOTCH1/HDAC2/HIF1A/MED16/TSC2/RHEB/PIK3R5/PLCB2/SLC9A1 |
| hsa04144:Endocytosis | 31 | 4.80E-05 | VPS29/ARFGAP1/USP8/SNX6/STAM2/SNX2/CYTH4/PIP5K1C/SNX4/ARF5/SNX3/CAPZB/GBF1/SH3GLB2/VPS35/EHD1/IQSEC1/GIT1/VPS45/VTA1/RAB4A/PSD4/RAB11FIP5/ACAP3/RAB35/CCDC53/GRK6/HGS/ARAP1/DNM2/SH3GL1 |
| hsa04071:Sphingolipid signaling pathway | 20 | 4.96E-05 | PPP2R1A/TRAF2/SPTLC1/GNAI2/MAP2K2/PPP2R5D/PIK3CD/TP53/AKT1/TNFRSF1A/RAC2/S1PR4/MAPK14/PPP2CB/PIK3R5/MAPK8/PLCB2/PPP2R3C/PPP2R2A/DEGS1 |
| hsa05220:Chronic myeloid leukemia | 14 | 2.03E-04 | AKT1/HDAC2/BCR/MAP2K2/STAT5A/PIK3CD/STAT5B/ARAF/TP53/SHC1/PIK3R5/RB1/RUNX1/CHUK |
| hsa05221:Acute myeloid leukemia | 12 | 2.90E-04 | AKT1/PPARD/MAP2K2/STAT5A/PIK3CD/STAT5B/ARAF/PIM1/RARA/PIK3R5/RUNX1/CHUK |
| hsa03015:mRNA surveillance pathway | 15 | 6.52E-04 | SYMPK/PPP2R1A/UPF1/SMG6/SMG5/PPP2R5D/HBS1L/CASC3/DAZAP1/PAPOLA/UPF3B/PPP2CB/CPSF3/PPP2R3C/PPP2R2A |
| hsa05200:Pathways in cancer | 39 | 1.04E-03 | TRAF2/PPARD/ADCY7/GNAI2/STAT5A/STAT5B/EGLN2/MLH1/LPAR2/NFKB2/RBX1/AKT1/RAC2/RASGRP2/RARA/PIK3R5/RUNX1/PLCB2/CHUK/DVL2/DVL3/ARHGEF1/BCR/PTGER4/MAP2K2/RXRB/RXRA/CREBBP/PIK3CD/TP53/ITGA3/RB1/BIRC2/DVL1/HIF1A/HDAC2/GNB1/ARAF/MAPK8 |
| hsa05203:Viral carcinogenesis | 24 | 1.57E-03 | TRAF2/ACTN4/HIST1H2BF/STAT5A/CREBBP/STAT5B/PIK3CD/TP53/HDAC10/RB1/NFKB2/SRF/GTF2B/VDAC3/HDAC4/HDAC2/SND1/ATF6B/GTF2A2/PIK3R5/JAK3/TBPL1/HDAC7/HDAC6 |
| hsa05169:Epstein-Barr virus infection | 17 | 1.63E-03 | IRAK1/TRAF2/TBK1/PIK3CD/TP53/RB1/NFKB2/AKT1/TYK2/HDAC2/MAPK14/PIK3R5/MAPK8/JAK3/MAP3K14/NCOR2/CHUK |
| hsa05016:Huntington's disease | 22 | 3.38E-03 | NDUFA4/NDUFB3/NDUFB4/TAF4/NDUFB5/COX7A2/NDUFB6/CREBBP/TP53/NDUFC2/DNAH1/UQCRQ/VDAC3/DCTN1/COX6C/VDAC1/UQCRHL/POLR2A/HDAC2/ATP5C1/PLCB2/TBPL1 |
| hsa05160:Hepatitis C | 17 | 3.97E-03 | PPP2R1A/TRAF2/TBK1/RXRA/PIK3CD/TP53/IFNAR1/AKT1/TYK2/TNFRSF1A/MAPK14/PPP2CB/ARAF/PIK3R5/MAPK8/CHUK/PPP2R2A |
| hsa05211:Renal cell carcinoma | 11 | 4.22E-03 | AKT1/HIF1A/MAP2K2/PIK3CD/CREBBP/ARAF/RAP1A/EGLN2/RAP1B/PIK3R5/RBX1 |
| hsa03018:RNA degradation | 12 | 4.38E-03 | PATL1/EXOSC8/PFKL/SKIV2L/CNOT3/LSM5/CNOT2/EDC4/CNOT7/LSM1/TOB2/XRN2 |
| hsa05231:Choline metabolism in cancer | 14 | 5.11E-03 | MAP2K2/WASF2/PIK3CD/PIP5K1C/LYPLA1/CHPT1/AKT1/HIF1A/RAC2/TSC2/RHEB/DGKZ/PIK3R5/MAPK8 |
| hsa04010:MAPK signaling pathway | 26 | 5.61E-03 | TRAF2/MKNK2/CACNB1/NFKB2/SRF/AKT1/TNFRSF1A/RAC2/RASGRP2/JUND/CHUK/TAOK2/MAP2K2/TP53/ECSIT/FLNA/CDC25B/RPS6KA4/MAPK14/RAP1A/MAPK8IP3/RAP1B/MAPK8/MAP3K14/MAP3K12/MAP3K11 |
| hsa05166:HTLV-I infection | 26 | 5.90E-03 | DVL2/DVL3/ANAPC5/ADCY7/STAT5A/CREBBP/STAT5B/PIK3CD/TP53/ITGB2/RB1/NFKB2/CDC16/VDAC3/SRF/DVL1/VDAC1/AKT1/TNFRSF1A/POLD1/PIK3R5/JAK3/MAP3K14/TCF3/TBPL1/CHUK |
| hsa04932:Non-alcoholic fatty liver disease (NAFLD) | 18 | 5.99E-03 | NDUFA4/NDUFB3/NDUFB4/TRAF2/NDUFB5/COX7A2/NDUFB6/RXRA/PIK3CD/NDUFC2/UQCRQ/COX6C/UQCRHL/AKT1/TNFRSF1A/PIK3R5/MAPK8/MAP3K11 |
| hsa05142:Chagas disease (American trypanosomiasis) | 14 | 6.55E-03 | IRAK1/PPP2R1A/GNAI2/PIK3CD/AKT1/TNFRSF1A/ACE/MAPK14/PPP2CB/PIK3R5/MAPK8/PLCB2/CHUK/PPP2R2A |
| hsa04210:Apoptosis | 10 | 8.64E-03 | AKT1/TRAF2/TNFRSF1A/PIK3CD/TP53/PIK3R5/MAP3K14/BIRC2/CHUK/CAPN1 |
| hsa04914:Progesterone-mediated oocyte maturation | 12 | 1.10E-02 | AKT1/FZR1/GNAI2/ANAPC5/ADCY7/MAPK14/PIK3CD/ARAF/MAPK8/PIK3R5/CDC16/CDC25B |
| hsa05223:Non-small cell lung cancer | 9 | 1.42E-02 | AKT1/RXRB/MAP2K2/RXRA/PIK3CD/ARAF/TP53/PIK3R5/RB1 |
| hsa05012:Parkinson's disease | 16 | 1.65E-02 | NDUFA4/NDUFB3/NDUFB4/NDUFB5/COX7A2/NDUFB6/GNAI2/UBE2G1/NDUFC2/UQCRQ/VDAC3/COX6C/UQCRHL/VDAC1/UBA1/ATP5C1 |
| hsa04330:Notch signaling pathway | 8 | 1.92E-02 | DVL2/DVL3/NOTCH1/HDAC2/CREBBP/LFNG/NCOR2/DVL1 |
| hsa05161:Hepatitis B | 16 | 1.97E-02 | TBK1/MAP2K2/STAT5A/CREBBP/STAT5B/PIK3CD/TP53/RB1/VDAC3/IFNAR1/AKT1/PTK2B/ATF6B/PIK3R5/MAPK8/CHUK |
| hsa04917:Prolactin signaling pathway | 10 | 2.01E-02 | AKT1/MAP2K2/MAPK14/STAT5A/PIK3CD/STAT5B/GALT/MAPK8/SHC1/PIK3R5 |
| hsa05222:Small cell lung cancer | 11 | 2.38E-02 | AKT1/TRAF2/RXRB/RXRA/PIK3CD/TP53/ITGA3/PIK3R5/RB1/BIRC2/CHUK |
| hsa04110:Cell cycle | 14 | 2.61E-02 | FZR1/E2F4/E2F5/ANAPC5/CREBBP/TP53/CDC16/RB1/MCM5/RBX1/CDC25B/HDAC2/ORC4/ORC3 |
| hsa04120:Ubiquitin mediated proteolysis | 15 | 2.61E-02 | CUL3/FZR1/CUL5/SYVN1/ANAPC5/CUL7/PIAS4/HUWE1/WWP2/UBA1/UBE2G1/UBA3/CDC16/BIRC2/RBX1 |
| hsa04261:Adrenergic signaling in cardiomyocytes | 15 | 2.76E-02 | PPP2R1A/ATP1B3/GNAI2/ADCY7/PPP2R5D/CACNB1/ATP1A1/AKT1/MAPK14/PPP2CB/ATF6B/PLCB2/PPP2R3C/SLC9A1/PPP2R2A |
| hsa05212:Pancreatic cancer | 9 | 3.24E-02 | AKT1/RAC2/PIK3CD/ARAF/TP53/MAPK8/PIK3R5/RB1/CHUK |
| hsa05010:Alzheimer's disease | 17 | 3.26E-02 | NDUFA4/NDUFB3/NDUFB4/NDUFB5/COX7A2/NDUFB6/NDUFC2/UQCRQ/COX6C/NAE1/CAPN1/UQCRHL/TNFRSF1A/LRP1/ATP2A3/ATP5C1/PLCB2 |
| hsa04810:Regulation of actin cytoskeleton | 20 | 3.37E-02 | GIT1/ARHGEF1/ACTN4/LIMK1/MAP2K2/DIAPH1/PPP1R12C/WASF2/PIK3CD/PIP5K1C/ARHGAP35/ITGB2/ITGA3/MYL12A/RAC2/ITGA5/ARAF/PIK3R5/FGD3/SLC9A1 |
| hsa04611:Platelet activation | 14 | 3.65E-02 | ORAI1/ARHGEF1/GNAI2/ADCY7/PIK3CD/ARHGAP35/MYL12A/AKT1/MAPK14/RASGRP2/RAP1A/PIK3R5/RAP1B/PLCB2 |
| hsa04062:Chemokine signaling pathway | 18 | 3.96E-02 | ADCY7/GNAI2/PREX1/STAT5B/PIK3CD/AKT1/RAC2/GNB1/PTK2B/RASGRP2/GRK6/RAP1A/PIK3R5/SHC1/RAP1B/JAK3/PLCB2/CHUK |
| hsa03040:Spliceosome | 14 | 4.27E-02 | CHERP/SRSF10/SNRPB2/SF3A2/SF3A1/SMNDC1/SRSF3/DDX46/PLRG1/PRPF8/LSM5/SNRNP27/SNRPG/RBM17 |
| hsa05162:Measles | 14 | 4.27E-02 | TYK2/AKT1/IRAK1/TBK1/EIF3H/CD46/STAT5A/PIK3CD/STAT5B/TP53/PIK3R5/JAK3/CHUK/IFNAR1 |
| hsa04722:Neurotrophin signaling pathway | 13 | 4.34E-02 | AKT1/IRAK1/MAP2K2/MAPK14/PIK3CD/TP53/RAP1A/RAP1B/MAPK8/SHC1/PIK3R5/SH2B1/ARHGDIA |
| hsa04668:TNF signaling pathway | 12 | 4.38E-02 | AKT1/TRAF2/TNFRSF1A/RPS6KA4/MAPK14/PIK3CD/ATF6B/MAPK8/PIK3R5/MAP3K14/BIRC2/CHUK |

Supplementary Table 8 KEGG pathway and DEGs at 6 months after STEMI

| KEGG PATHWAY | Count | PValue | Gene |
| --- | --- | --- | --- |
| hsa05221:Acute myeloid leukemia | 12 | 9.21E-06 | PPARD/KRAS/MAP2K2/PIK3CD/ARAF/SOS2/PIM1/RAF1/PIK3R5/RUNX1/CHUK/PIK3R2 |
| hsa04144:Endocytosis | 25 | 2.50E-05 | ARFGAP1/VPS29/USP8/RAB5B/SNX6/KIAA0196/PIP5K1C/SNX4/EPS15L1/CHMP2B/SH3GLB2/GBF1/RAB11B/IL2RG/EHD1/VPS36/AGAP2/GIT1/RAB4A/SMAD3/ACAP3/RAB35/GRK6/HGS/SH3GL1 |
| hsa05213:Endometrial cancer | 11 | 2.87E-05 | KRAS/MAP2K2/PIK3CD/ARAF/SOS2/TP53/RAF1/MLH1/ELK1/PIK3R5/PIK3R2 |
| hsa05220:Chronic myeloid leukemia | 12 | 1.06E-04 | KRAS/HDAC2/MAP2K2/PIK3CD/ARAF/SOS2/TP53/RAF1/PIK3R5/RUNX1/CHUK/PIK3R2 |
| hsa03040:Spliceosome | 16 | 2.24E-04 | HNRNPA1L2/CHERP/LSM8/LSM6/SF3A2/SF3A1/RBMX/HNRNPA1/HNRNPK/DDX46/ISY1/DHX15/SLU7/SNRPE/SNRPG/RBM17 |
| hsa05211:Renal cell carcinoma | 11 | 2.35E-04 | KRAS/MAP2K2/PAK4/PIK3CD/ARAF/SOS2/RAP1A/RAF1/RAP1B/PIK3R5/PIK3R2 |
| hsa05203:Viral carcinogenesis | 20 | 4.52E-04 | TRAF2/HIST1H2BC/HIST1H2BF/PIK3CD/TP53/HDAC10/NFKB2/SRF/GTF2B/PXN/CCR8/HDAC2/HNRNPK/KRAS/GTF2A2/PSMC1/PIK3R5/JAK3/HDAC7/PIK3R2 |
| hsa04810:Regulation of actin cytoskeleton | 20 | 6.07E-04 | GIT1/MYL5/ARHGEF1/VAV3/MAP2K2/PIK3CD/RAF1/PIP5K1C/ITGA3/MYL12A/PXN/PFN1/KRAS/RAC2/PAK4/SOS2/ARAF/BRK1/PIK3R5/PIK3R2 |
| hsa05210:Colorectal cancer | 10 | 6.66E-04 | KRAS/RAC2/PIK3CD/ARAF/TP53/RAF1/SMAD3/MLH1/PIK3R5/PIK3R2 |
| hsa05212:Pancreatic cancer | 10 | 9.46E-04 | KRAS/RAC2/PIK3CD/ARAF/TP53/RAF1/SMAD3/PIK3R5/CHUK/PIK3R2 |
| hsa05214:Glioma | 10 | 9.46E-04 | KRAS/CALML3/MAP2K2/PIK3CD/ARAF/SOS2/TP53/RAF1/PIK3R5/PIK3R2 |
| hsa04662:B cell receptor signaling pathway | 10 | 1.46E-03 | VAV3/KRAS/RAC2/MAP2K2/PIK3CD/SOS2/RAF1/PIK3R5/CHUK/PIK3R2 |
| hsa05223:Non-small cell lung cancer | 9 | 1.47E-03 | KRAS/MAP2K2/PIK3CD/ARAF/SOS2/TP53/RAF1/PIK3R5/PIK3R2 |
| hsa04919:Thyroid hormone signaling pathway | 13 | 1.87E-03 | MAP2K2/PIK3CD/TP53/MED12/MED24/RAF1/KRAS/HDAC2/MED17/TSC2/RHEB/PIK3R5/PIK3R2 |
| hsa05231:Choline metabolism in cancer | 12 | 2.03E-03 | KRAS/RAC2/MAP2K2/PIK3CD/TSC2/SOS2/RAF1/RHEB/PIP5K1C/LYPLA1/PIK3R5/PIK3R2 |
| hsa04010:MAPK signaling pathway | 21 | 2.33E-03 | TRAF2/MAP2K2/TP53/CACNB1/PPM1A/MAP4K2/RAF1/ELK1/NFKB2/DAXX/SRF/CDC25B/KRAS/RAC2/MAP3K2/JUND/SOS2/RAP1A/RAP1B/CHUK/MAP3K12 |
| hsa05166:HTLV-I infection | 21 | 2.44E-03 | ADCY3/DVL3/WNT10A/SLC25A6/PIK3CD/TP53/SMAD3/ELK1/NFKB2/TRRAP/CANX/SRF/DVL1/KRAS/RANBP3/PIK3R5/IL2RG/JAK3/TCF3/CHUK/PIK3R2 |
| hsa04370:VEGF signaling pathway | 9 | 2.57E-03 | KRAS/RAC2/SPHK2/MAP2K2/PIK3CD/RAF1/PIK3R5/PXN/PIK3R2 |
| hsa04071:Sphingolipid signaling pathway | 13 | 2.68E-03 | TRAF2/SPHK2/MAP2K2/PPP2R5D/PIK3CD/TP53/RAF1/KRAS/RAC2/PPP2CB/PIK3R5/PIK3R2/DEGS1 |
| hsa04062:Chemokine signaling pathway | 17 | 2.73E-03 | ADCY3/VAV3/PIK3CD/RAF1/CXCR3/PXN/CCR8/KRAS/RAC2/SOS2/GRK6/RAP1A/PIK3R5/RAP1B/JAK3/CHUK/PIK3R2 |
| hsa04664:Fc epsilon RI signaling pathway | 9 | 5.09E-03 | VAV3/KRAS/RAC2/MAP2K2/PIK3CD/SOS2/RAF1/PIK3R5/PIK3R2 |
| hsa04068:FoxO signaling pathway | 13 | 6.56E-03 | SGK2/MAP2K2/PIK3CD/RAF1/SMAD3/KRAS/ARAF/SOS2/PIK3R5/KLF2/AGAP2/CHUK/PIK3R2 |
| hsa04012:ErbB signaling pathway | 10 | 7.14E-03 | KRAS/MAP2K2/PAK4/PIK3CD/ARAF/SOS2/RAF1/ELK1/PIK3R5/PIK3R2 |
| hsa04722:Neurotrophin signaling pathway | 12 | 7.66E-03 | KRAS/CALML3/MAP2K2/PIK3CD/SOS2/TP53/RAP1A/RAF1/RAP1B/PIK3R5/ARHGDIA/PIK3R2 |
| hsa05215:Prostate cancer | 10 | 7.68E-03 | KRAS/MAP2K2/PIK3CD/ARAF/SOS2/TP53/RAF1/PIK3R5/CHUK/PIK3R2 |
| hsa05169:Epstein-Barr virus infection | 12 | 8.65E-03 | TRAF2/PSMC6/HDAC2/PIK3CD/PSMC1/SHFM1/TP53/PIK3R5/NFKB2/JAK3/CHUK/PIK3R2 |
| hsa04015:Rap1 signaling pathway | 17 | 8.79E-03 | ADCY3/ADORA2A/MAP2K2/CSF1/PIK3CD/RAF1/LPAR2/RGS14/PFN1/KRAS/RAC2/CALML3/RAP1A/PIK3R5/RAP1B/PRKD3/PIK3R2 |
| hsa05200:Pathways in cancer | 26 | 1.21E-02 | ADCY3/DVL3/WNT10A/TRAF2/PPARD/ARHGEF1/MAP2K2/PIK3CD/TP53/MLH1/SMAD3/RAF1/LPAR2/ITGA3/NFKB2/DVL1/FLT3LG/KRAS/HDAC2/RAC2/ARAF/SOS2/PIK3R5/RUNX1/CHUK/PIK3R2 |
| hsa05230:Central carbon metabolism in cancer | 8 | 1.27E-02 | KRAS/MAP2K2/PIK3CD/TP53/RAF1/SIRT6/PIK3R5/PIK3R2 |
| hsa00190:Oxidative phosphorylation | 12 | 1.59E-02 | NDUFA4/NDUFB3/COX7A2/NDUFB6/NDUFA8/COX7B/ATP5C1/NDUFAB1/ATP5L/NDUFA1/PPA2/UQCRHL |
| hsa05160:Hepatitis C | 12 | 1.59E-02 | TRAF2/KRAS/PIK3CD/PPP2CB/ARAF/SOS2/TP53/RAF1/PIK3R5/CHUK/IFNAR1/PIK3R2 |
| hsa04932:Non-alcoholic fatty liver disease (NAFLD) | 13 | 1.62E-02 | NDUFB3/NDUFA4/TRAF2/NDUFB6/COX7A2/NDUFA8/PIK3CD/COX7B/NDUFAB1/NDUFA1/UQCRHL/PIK3R5/PIK3R2 |
| hsa04660:T cell receptor signaling pathway | 10 | 1.70E-02 | VAV3/KRAS/MAP2K2/PAK4/PIK3CD/SOS2/RAF1/PIK3R5/CHUK/PIK3R2 |
| hsa04910:Insulin signaling pathway | 12 | 2.04E-02 | KRAS/CALML3/MAP2K2/PIK3CD/TSC2/ARAF/SOS2/RAF1/RHEB/ELK1/PIK3R5/PIK3R2 |
| hsa05218:Melanoma | 8 | 2.15E-02 | KRAS/MAP2K2/PIK3CD/ARAF/TP53/RAF1/PIK3R5/PIK3R2 |
| hsa04917:Prolactin signaling pathway | 8 | 2.15E-02 | KRAS/MAP2K2/PIK3CD/SOS2/RAF1/PIK3R5/CISH/PIK3R2 |
| hsa04650:Natural killer cell mediated cytotoxicity | 11 | 2.22E-02 | VAV3/KRAS/RAC2/MAP2K2/PIK3CD/ARAF/SOS2/RAF1/PIK3R5/IFNAR1/PIK3R2 |
| hsa04550:Signaling pathways regulating pluripotency of stem cells | 12 | 2.24E-02 | DVL3/WNT10A/KRAS/MAP2K2/PIK3CD/RAF1/SMAD3/PIK3R5/JAK3/TCF3/PIK3R2/DVL1 |
| hsa05034:Alcoholism | 14 | 2.28E-02 | HIST1H2BC/ADORA2A/HIST1H2BF/HDAC10/RAF1/HAT1/PDYN/KRAS/HDAC2/CALML3/ARAF/SOS2/H3F3A/HDAC7 |
| hsa05012:Parkinson's disease | 12 | 2.46E-02 | NDUFA4/NDUFB3/COX7A2/NDUFB6/NDUFA8/ADORA2A/SLC25A6/COX7B/ATP5C1/NDUFAB1/NDUFA1/UQCRHL |
| hsa03050:Proteasome | 6 | 2.87E-02 | PSMA1/PSMC6/PSMA6/PSMC1/SHFM1/PSME4 |
| hsa04962:Vasopressin-regulated water reabsorption | 6 | 2.87E-02 | ADCY3/RAB5B/RAB11B/VAMP2/AQP3/ARHGDIA |
| hsa04510:Focal adhesion | 15 | 3.33E-02 | MYL5/VAV3/PIK3CD/RAF1/ELK1/ITGA3/MYL12A/PXN/RAC2/PAK4/SOS2/RAP1A/PIK3R5/RAP1B/PIK3R2 |
| hsa04014:Ras signaling pathway | 16 | 3.38E-02 | RAB5B/MAP2K2/CSF1/PIK3CD/RAF1/ELK1/KRAS/RAC2/CALML3/PAK4/SOS2/RAP1A/PIK3R5/RAP1B/CHUK/PIK3R2 |
| hsa04670:Leukocyte transendothelial migration | 10 | 3.78E-02 | VAV3/MYL5/RAC2/PIK3CD/RAP1A/RAP1B/PIK3R5/MYL12A/PXN/PIK3R2 |
| hsa04330:Notch signaling pathway | 6 | 3.99E-02 | DVL3/MFNG/CIR1/HDAC2/DTX3/DVL1 |
| hsa05016:Huntington's disease | 14 | 4.04E-02 | NDUFB3/NDUFA4/COX7A2/NDUFB6/NDUFA8/SLC25A6/COX7B/NDUFAB1/TP53/DNAH1/NDUFA1/UQCRHL/HDAC2/ATP5C1 |
| hsa04915:Estrogen signaling pathway | 9 | 4.16E-02 | ADCY3/KRAS/CALML3/MAP2K2/PIK3CD/SOS2/RAF1/PIK3R5/PIK3R2 |
| hsa04720:Long-term potentiation | 7 | 4.54E-02 | KRAS/CALML3/MAP2K2/ARAF/RAP1A/RAF1/RAP1B |
| hsa04666:Fc gamma R-mediated phagocytosis | 8 | 4.77E-02 | VAV3/RAC2/SPHK2/PIK3CD/RAF1/PIP5K1C/PIK3R5/PIK3R2 |
